# A safe and efficient synthesis of N-Boc-β^3^-amino acid methyl esters from α-amino acids: applications in the formal synthesis of sedum alkaloids†

**DOI:** 10.1039/d4ra07506d

**Published:** 2024-11-11

**Authors:** Bohua Long, Lijie Ren, Mengmeng Jiang, Shengquan Hu, Qianqian Jiang, Limin Li, Xuanluan Chen, Zhengzhi Wu

**Affiliations:** a The First Affiliated Hospital of Shenzhen University, Shenzhen Second People's Hospital Shenzhen 518035 China bhlong121@163.com renlijie72@126.com szwzz001@163.com; b Wu Zhengzhi Academician Workstation, Ningbo College of Health Sciences Ningbo 315800 People's Republic of China; c Shenzhen Institute of Geriatric Medicine Shenzhen 518035 China

## Abstract

β^3^-Amino acids are essential components in the synthesis of biologically active compounds. However, obtaining them in enantiomerically pure forms remains challenging. This study investigates a safe and efficient method for synthesizing enantiopure N-Boc-β^3^-amino acid methyl esters, incorporating both natural and unnatural side chains. The procedure avoids the use of expensive and toxic reagents, providing a safer alternative to the hazardous Arndt–Eistert homologation and cyanation reactions, which typically begin with enantiopure α-amino acids. The practical value of this transformation was demonstrated in the formal synthesis of sedum alkaloids.

## Abstract 2

β^3^-Amino acids are essential components in the synthesis of biologically active compounds.

## Graphical abstract

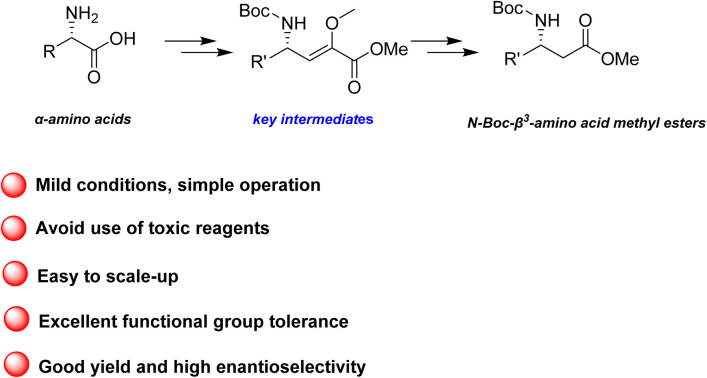

## Introduction

**Scheme 1:**
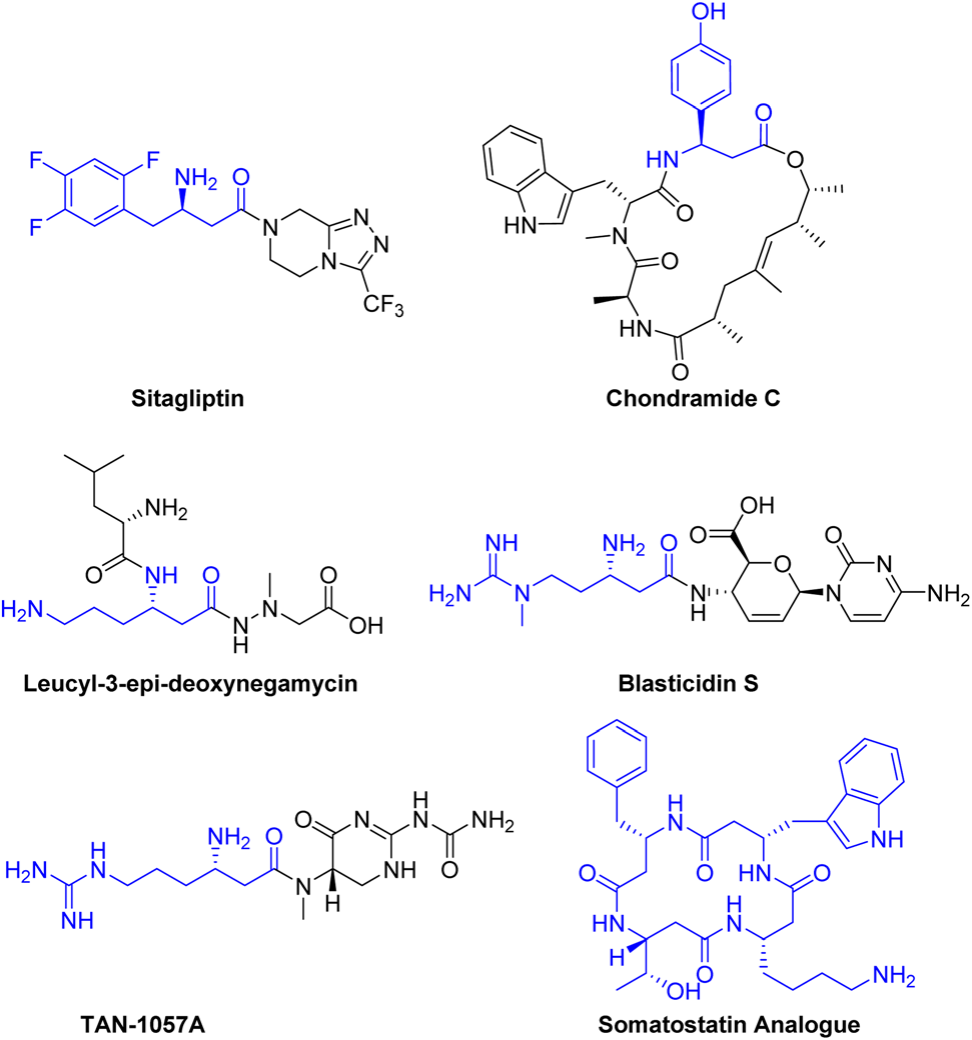
Representative bioactive molecules containing β^3^-amino acid moiety.

Many biologically active molecules, including drugs and natural products containing β^3^-amino acid fragments, exhibit a wide range of biological activities.^1^ For instance, sitagliptin, a potent and orally active dipeptidyl peptidase IV inhibitor, is used in the treatment of type 2 diabetes.^2^ Naturally occurring active peptides, including chondramide C,^3^ leucyl-3-epi-deoxynegamycin,^4^ blasticidin S^5^ and TAN-1057A^6^ have demonstrated significant therapeutic potential as anti-cancer agents, antimicrobials, and antibiotics (Scheme 1).

β^3^-Peptides can be regarded as peptidomimetics due to their remarkable stability against peptidases, which may enable oral bioavailability.^7^ For example, a somatostatin analogue composed of only four β^3^-amino acids is capable of mimicking the natural peptide hormone, exhibiting excellent biological activity and micromolar affinity for human receptors^8^ (Scheme 1).

β-Alanine, the only naturally occurring β^3^-amino acid, serves as a key precursor for the biosynthesis of vitamin B_5_ and coenzyme A. However, obtaining other β^3^-amino acids in enantiomerically pure form remains challenging. Therefore, the development of a method that can efficiently and quickly produce β^3^-amino acids from naturally derived α-amino acids would be highly valuable for exploratory research.

**Scheme 2:**
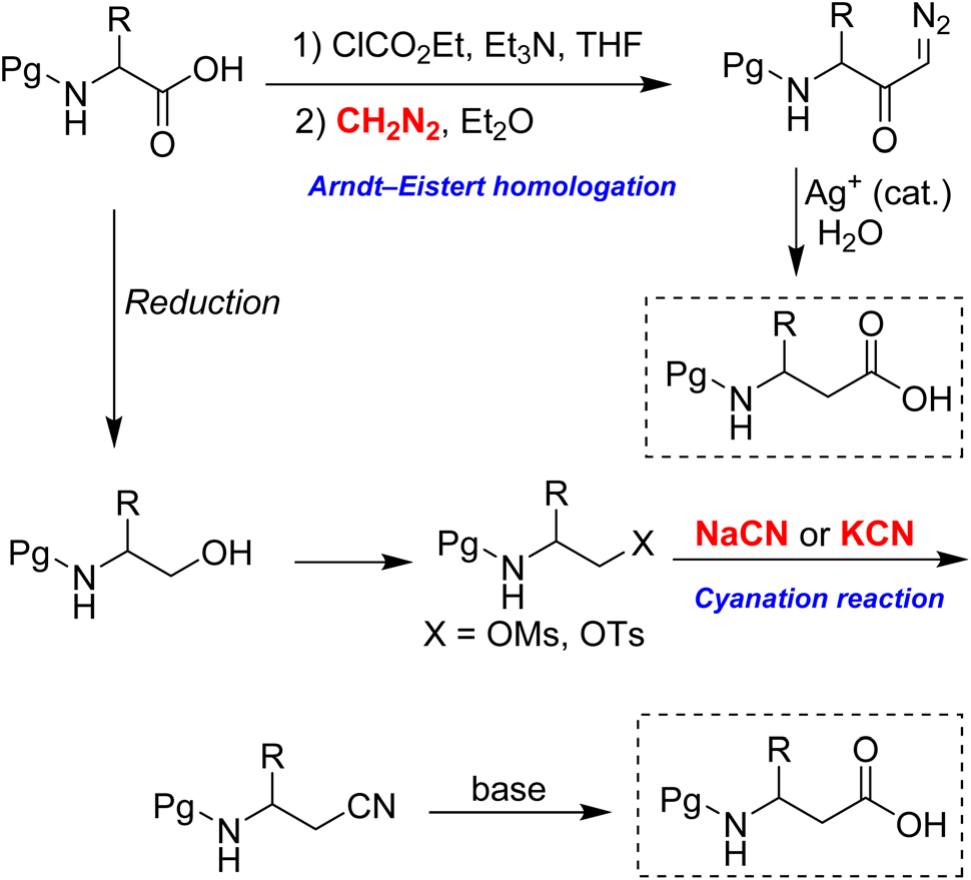
Two key synthetic pathways for the homologation of α-amino acids.

Current methods for homologating α-amino acids to β^3^-amino acids face several limitations. Among these, the Arndt–Eistert homologation is the most widely used and significant procedure for this conversion, as illustrated in Scheme 2.^9^ Although diazomethane is frequently employed in this reaction, it is highly hazardous due to its thermal instability, potential explosiveness, and extreme toxicity. Furthermore, it is typically prepared just before use, as it is unsuitable for long-term storage. Attempts to replace diazomethane with the safer TMS-diazomethane have proven unreliable, as this reagent cannot undergo acylation by mixed anhydrides.^10^

Another established method for performing the homologation is the cyanation reaction (Scheme 2).^11–14^ In this reaction, the alcohol group in N-protected α-amino alcohol is easily converted into β-amino cyanide through S_N_2 displacement of its mesylate or tosylate derivative. Following this, the cyanide group is transformed into the corresponding carboxylic acid. However, the method necessitates the extensive use of sodium cyanide or potassium cyanide, both of which are highly toxic and hazardous to humans, with an oral LD_50_ of approximately 1–2 mg kg^−1^.

In our efforts to develop a safe and efficient method for synthesising chiral β^3^-amino acids suitable for multigram production, we hypothesized, based on previous experience,^15^ that 2-methoxy-2-alkenoate generated through Wittig-type olefination could function as a key intermediate. The subsequent enol–keto isomerization, followed by reduction and oxidative one-carbon cleavage, makes this approach both feasible and appealing.

## Results and discussion

In our preliminary investigation, we explored the reaction of phosphonium salt 2^16^ with N-Boc-α-amino aldehydes, a combination that, to our knowledge, has not been previously studied. N-Boc-l-phenylalaninal 1a, synthesized using established literature procedures,^17^ was used as the model substrate.

As shown in Table 1, the use of aprotic solvents (THF, CH_2_Cl_2_, and MeCN) resulted in slow reactions with low yields. After thorough optimization, the highest yield was achieved using K_2_CO_3_ in i-PrOH at room temperature for 5 h (entry 9). When the amount of 1a was increased to 20 g, the product yield slightly decreased to 72% (entry 11).

**Table 1:** Optimization of Wittig-type reaction^a^

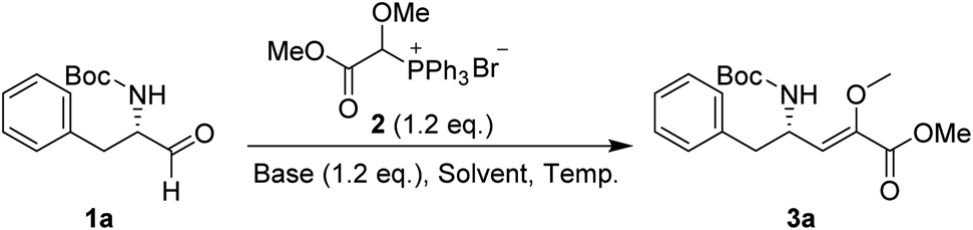					
Entry	Base	Solvent	Temp.	Time (h)	Yield^b^ (%)
1	DBU	THF	Reflux	24	30
3	DBU	CH_2_Cl_2_	Reflux	24	50
4	DBU/LiBr	THF	Reflux	24	30
5	TMG	THF	Reflux	24	32
4	TMG	MeCN	Reflux	24	35
5	TMG	CH_2_Cl_2_	Reflux	24	60
7	NaOMe	MeOH	RT	5	55
8	K_2_CO_3_	MeOH	RT	5	60
10	K_2_CO_3_	*t*-BuOH	RT	5	60
**9**	**K**_**2**_ **CO**_**3**_	**i-PrOH**	**RT**	5	**75**
**11** c	**K**_**2**_ **CO**_**3**_	**i-PrOH**	**RT**	**15**	**72**

a All reactions were performed with 1 mmol of 1a in 10 mL of solvent.

b Isolated yield.

c The reactions were performed with 20 g 1a in 300 mL of solvent.

**Scheme 3:**
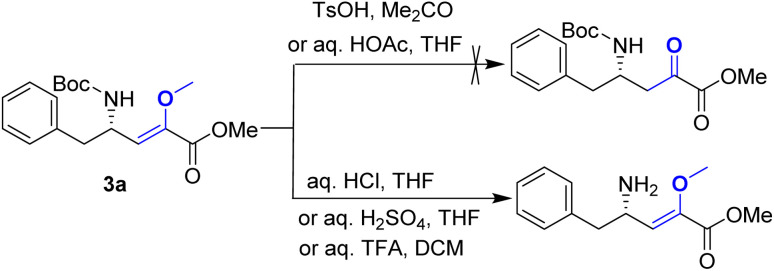
Attempt of the acid-promoted isomerization of 3a.

Upon obtaining the enol ether 3a, the subsequent acid-promoted isomerization reaction under standard conditions was carried out, utilizing TsOH, aqueous HOAc, aqueous HCl, aqueous H_2_SO_4_, and aqueous TFA. Unexpectedly, the reaction proved to be more challenging than anticipated, as the enol ether could not be transformed into the corresponding α-keto ester under acidic conditions (Scheme 3).

An alternative route was adopted, as detailed in Table 2. Initially, the reduction of the ester 3a using diisobutylaluminium hydride (DIBAL-H) yielded the allylic alcohol 4a in good yield. The conversion of 4a to 5a was then investigated to demonstrate the synthetic utility of the enol–keto isomerization process. After optimization, it has been noted that employing *p*-TsOH (100 mol%) in acetone at 0 °C for 1 h resulted in the best yield of 5a, achieving a 75% yield over the two-step process.

**Table 2:** Optimization of enol–keto isomerization^a^

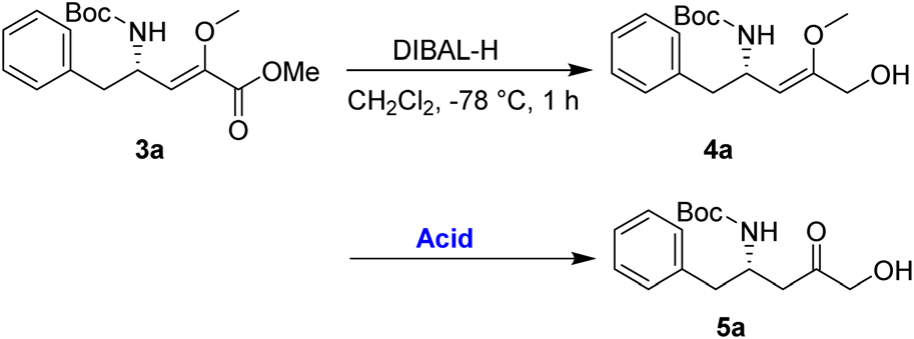			
Entry	Acid	Solvent	Yield^a^ (%)
1	aq. HOAc, rt, 2 h	THF	Trace
2	aq. HCl, rt, 2 h; then reprotected with Boc	THF	20
3	aq. TFA, rt, 2 h; then reprotected with Boc	CH_2_Cl_2_	25
4	TsOH (1.0 eq.), rt, 2 h	Me_2_CO	55
**5**	**TsOH (1.0 eq.), 0**°**C, 1 h**	**Me**_**2**_ **CO**	**75**

a Isolated yield over two steps.

**Scheme 4:**
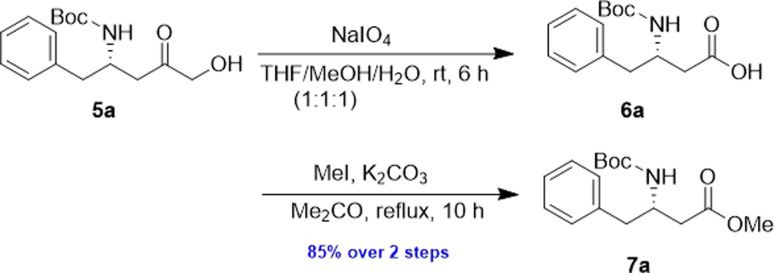
Preparation of 7a from 5a.

The α-keto compound 5a could be oxidized to the carboxylic acid 6a using periodic acid in aqueous THF; however, better results were achieved by treating the α-hydroxy ketone 5a with sodium periodate in a THF/MeOH/H_2_O mixture. This approach successfully yielded the N-Boc-β^3^-amino acid 6a, which was subsequently converted to the corresponding methyl ester 7a by reacting with MeI in refluxing acetone in the presence of anhydrous K_2_CO_3,_ achieving an overall yield of 85% over the two steps (Scheme 4).

To evaluate the generality of the optimal conditions outlined above, the preparation of other N-Boc-β^3^-amino acid methyl esters from α-amino acids was also investigated, with the results summarized in Table 3. All reactions proceeded successfully, yielding the corresponding products in high overall yields. The detailed procedures can be found in the ESI.†

**Table 3:** Synthesis of N-Boc-β^3^-amino acid methyl esters (7a–7k) from chiral α-amino acids

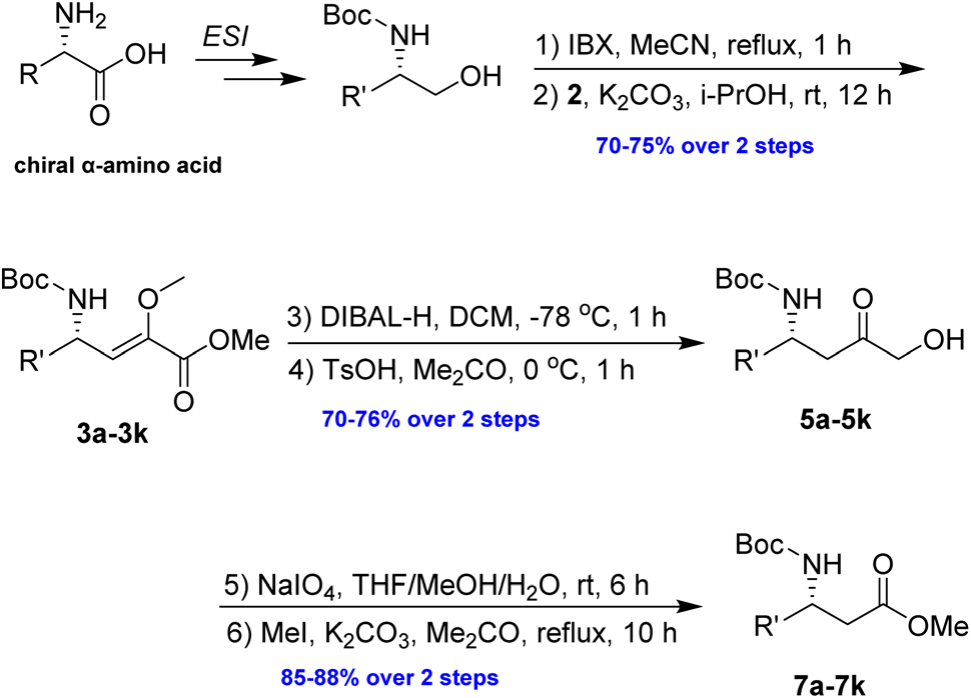		
Entry	Starting material (chiral α-amino acid)	Product (7a–7k) (N-Boc-β^3^-amino acid methyl ester)
1	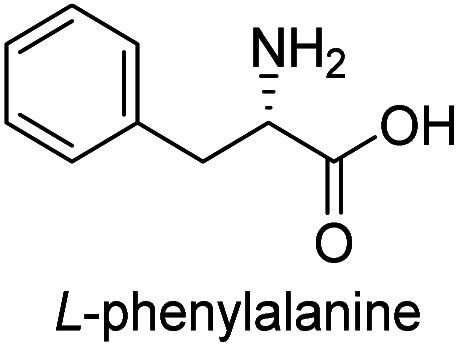	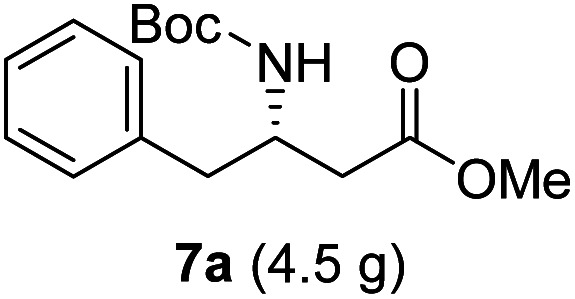
2	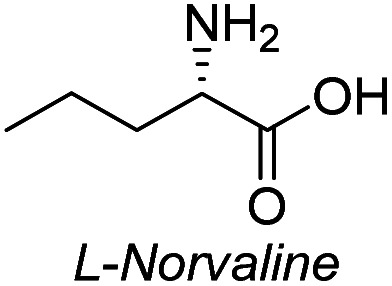	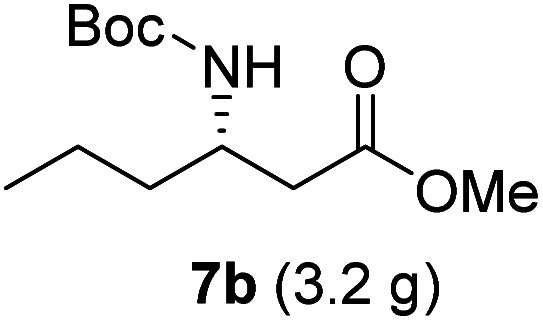
3	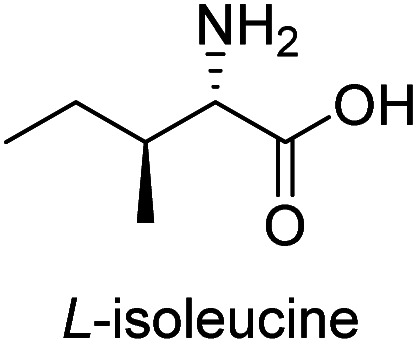	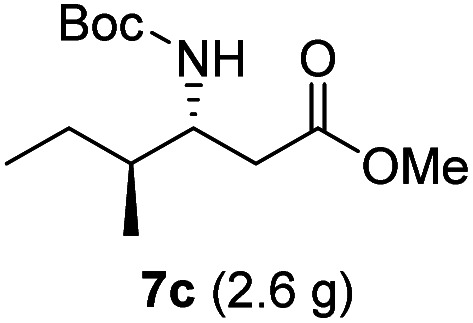
4	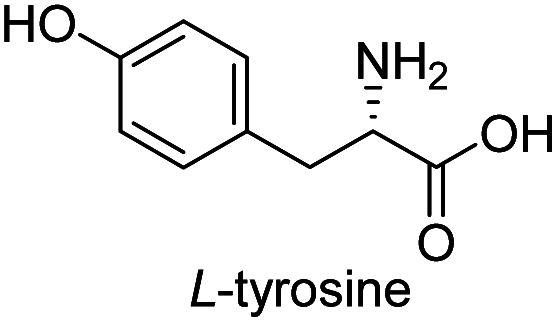	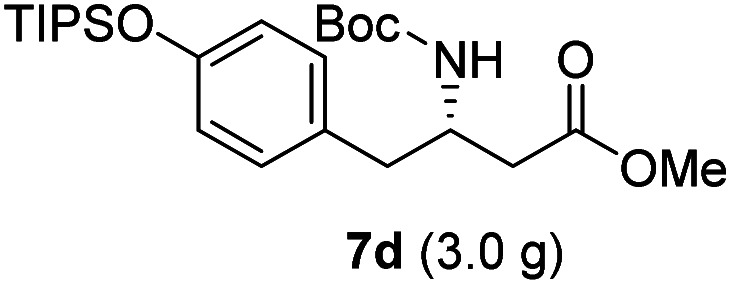
5	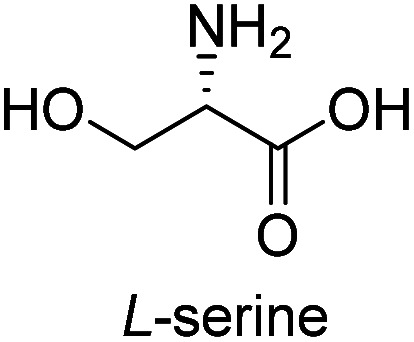	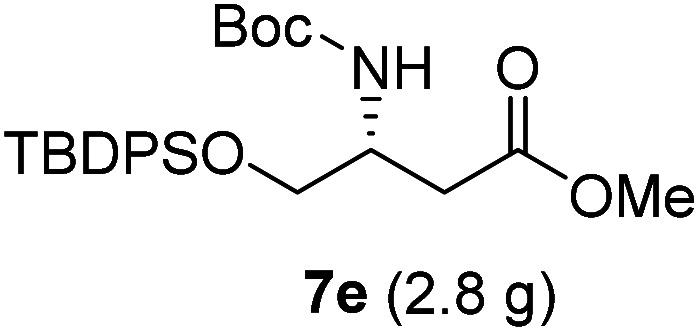
6	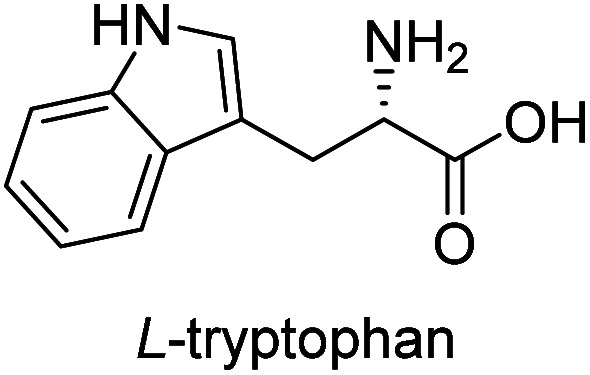	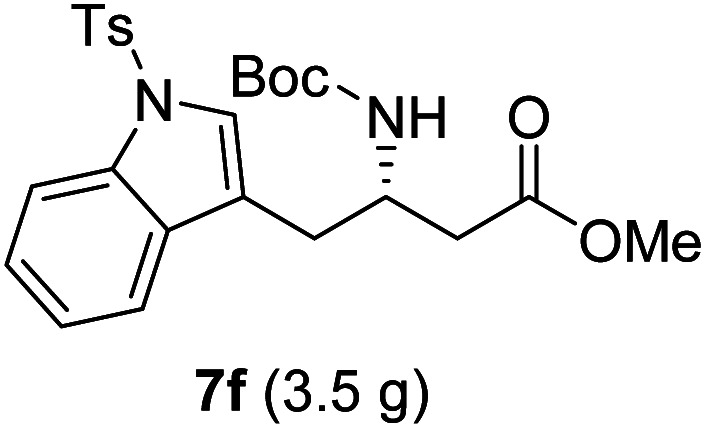
7	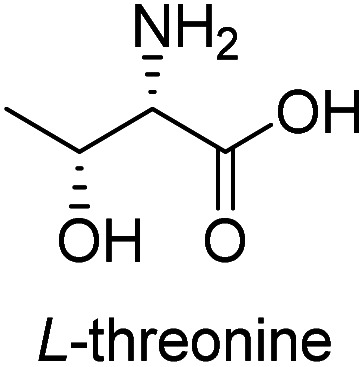	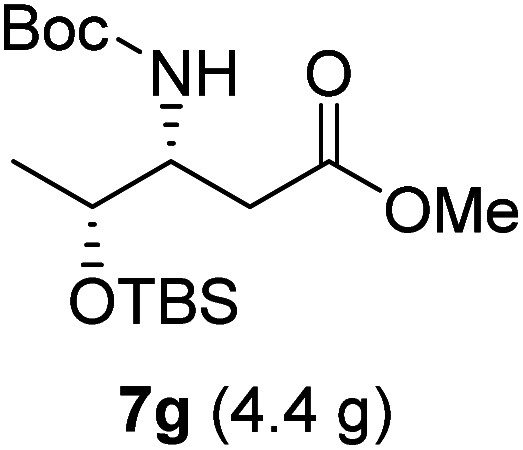
8	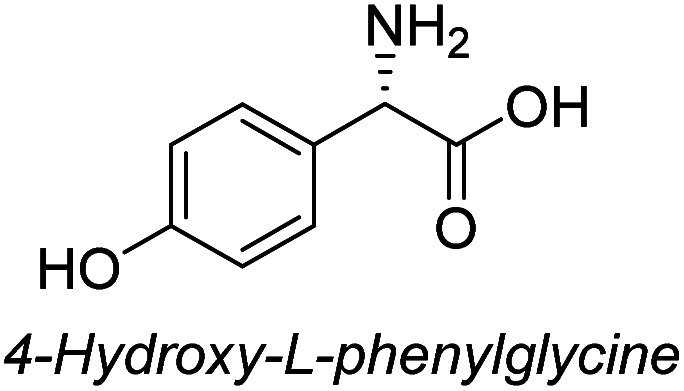	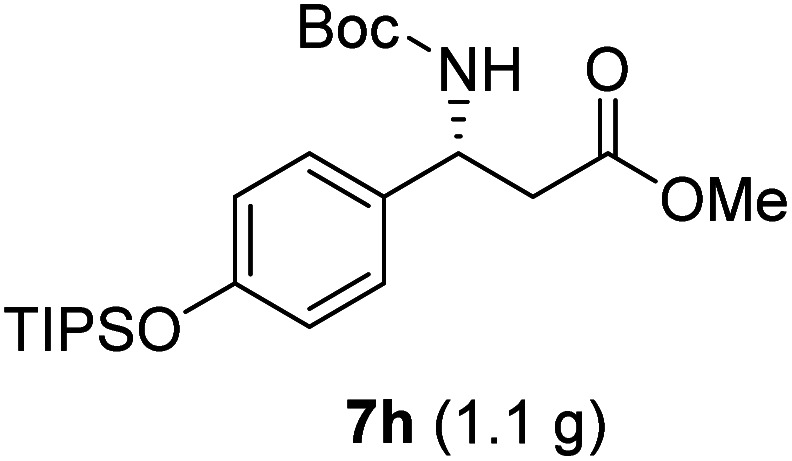
9	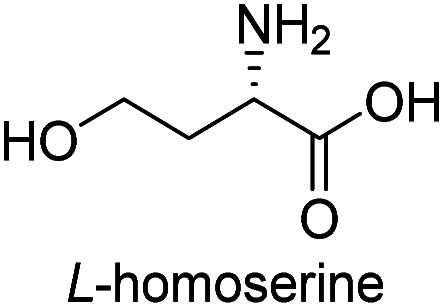	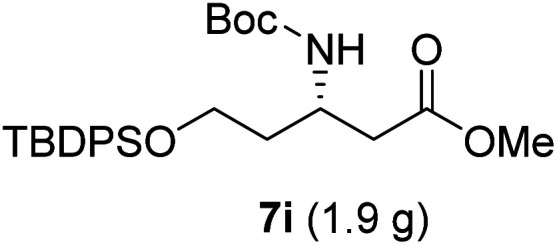
10	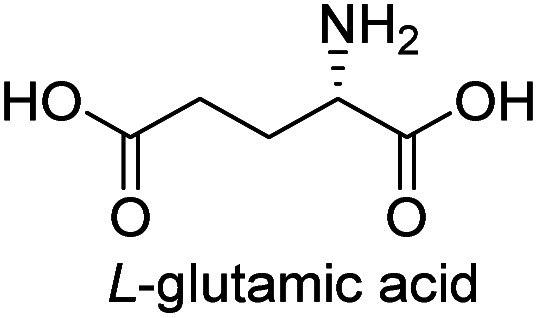	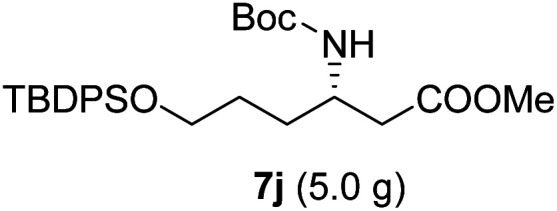
11	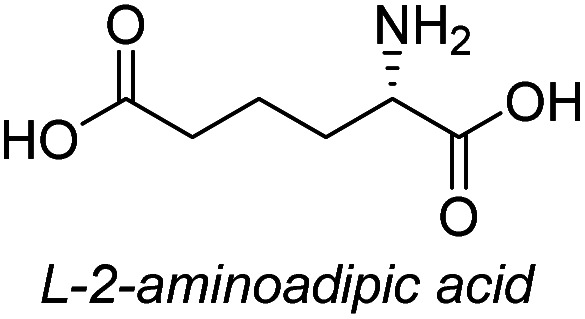	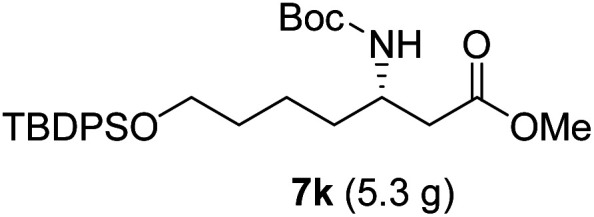

**Scheme 5:**
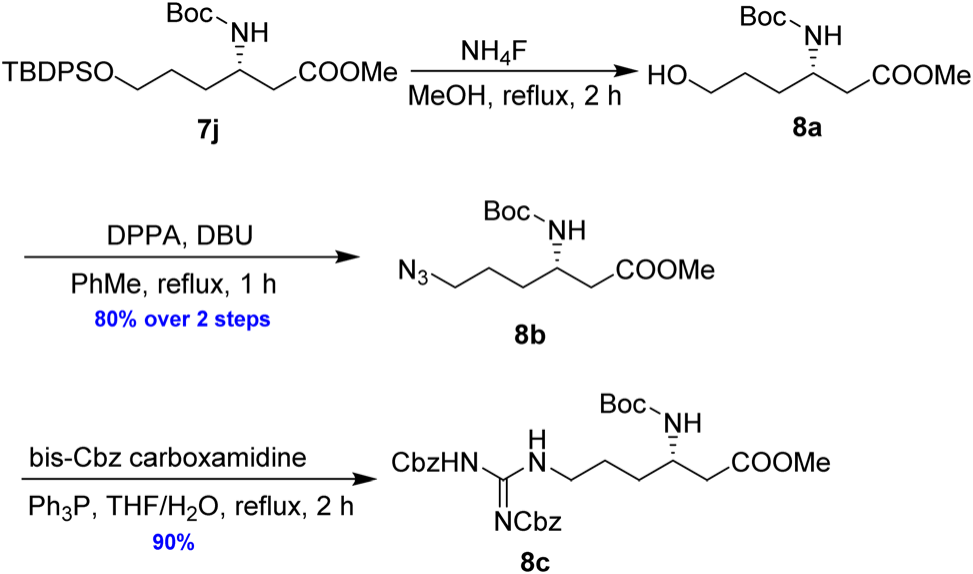
Preparation of 8b and 8c from 7j.

As illustrated in Scheme 5, the deprotection of the TBS ether in compound 7j using NH_4_F under reflux in MeOH yielded alcohol 8a. Subsequent reaction of 8a with DPPA and DBU in toluene led to its direct conversion into azide 8b in 80% yield over two steps, a key intermediate in the synthesis of the natural antibiotic leucyl-3-*epi*-deoxynegamycin and 3-*epi*-deoxynegamycin.^4^ Azide 8b was subsequently subjected to a one-pot reduction/guanidinylation, yielding the bis-Cbz-protected guanidine 8c in 90% yield. This compound served as a crucial intermediate in the synthesis of the peptide antibiotics TAN-1057A/B.^6^

**Scheme 6:**
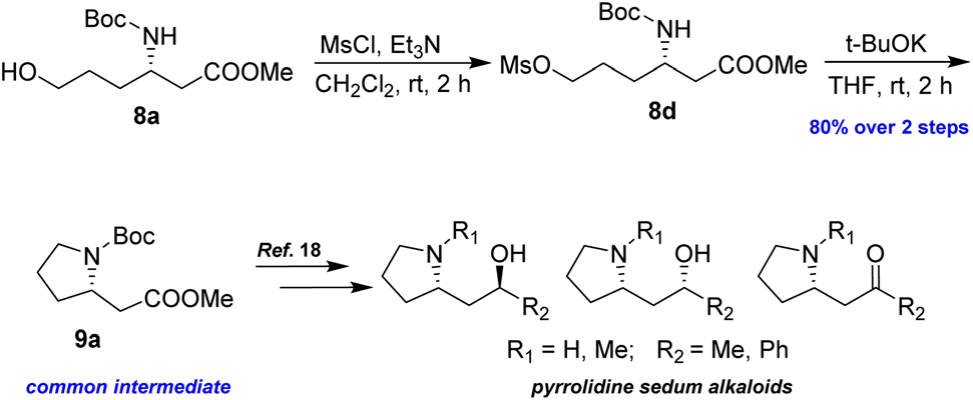
Preparation of 9a from 8a.

To demonstrate the efficacy of the new method in total synthesis, the formal total syntheses of the corresponding natural products were chosen. Compound 8a was subjected to primary alcohol activation using MsCl to facilitate cyclization, as illustrated in Scheme 6. Treatment with a strong base, NaH, in a THF/DMF mixture led to pyrrolidine 9a in 70% yield. A higher yield of 80% was achieved by using *t*-BuOK in THF at room temperature for 2 h. The formation of 9a represents the formal synthesis of pyrrolidine sedum alkaloids, as the enantiomer of 9a had previously been converted into these alkaloids by Davies and Fletcher.^18^

**Scheme 7:**
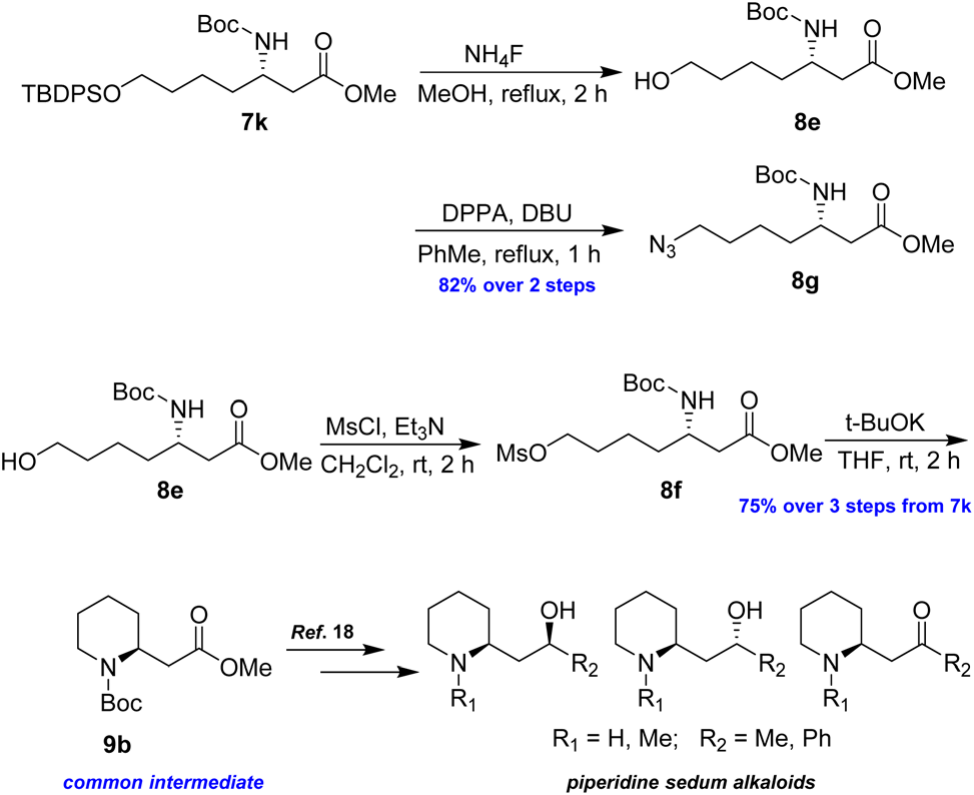
Preparation of 8g and 9b from 7k.

Following a similar synthetic route as described above, azide 8g and piperidine 9b were successfully prepared in good yields, representing formal syntheses of piperidine sedum alkaloids (Scheme 7).^18^

**Scheme 8:**
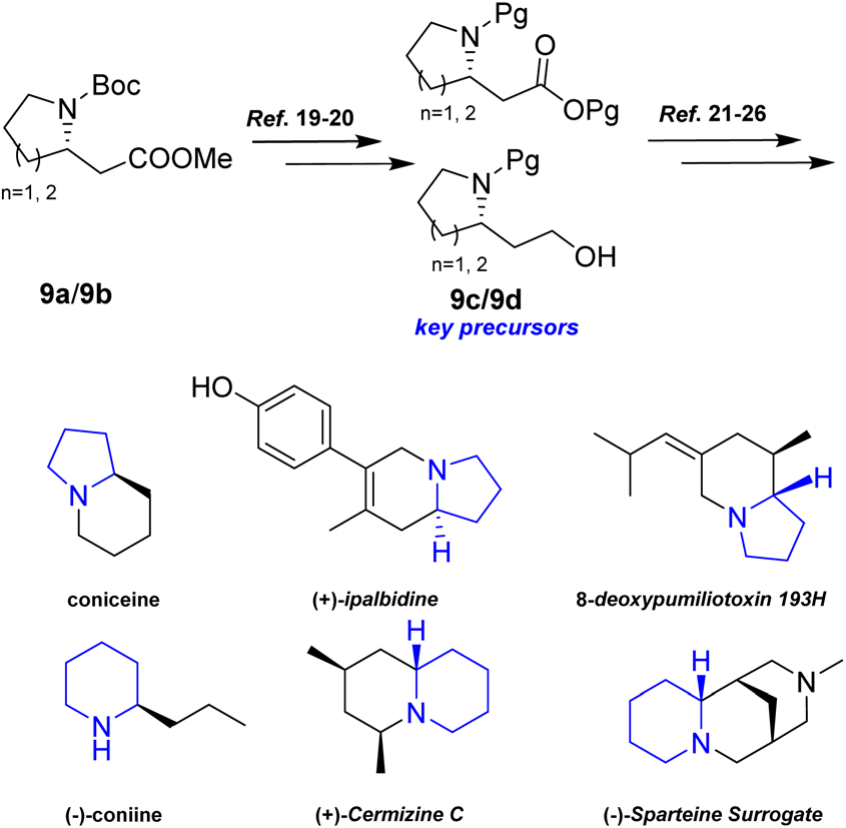
9c/9b served as key precursors of various alkaloids.

Additionally, intermediates 9a and 9b were readily converted into 9c/9b,^19,20^ which serve as key precursors for the stereoselective total synthesis of various alkaloids, including coniceine,^21^ (−)-coniine,^22^ 8-deoxypumiliotoxin 193*H*,^23^ (+)-ipalbidine,^24^ (+)-cermizine C^25^, and (−)-sparteine surrogate,^26^ (Scheme 8).

## Conclusions

In conclusion, the proposed method offers a safe, efficient, and scalable approach for the preparation of chiral N-Boc-β^3^-amino acid methyl esters from α-amino acids. The two-carbon elongation was achieved through a Wittig-type reaction, utilizing a methoxyphosphonium ylide generated from the phosphonium salt and potassium carbonate in isopropanol. The key intermediate, methyl 2-methoxy-2-alkenoate, was subsequently subjected to DIBAL-H reduction, followed by enol–keto isomerization, oxidative cleavage, and final methylation. Key features of the synthesis included the following: (1) it avoids the use of expensive and hazardous reagents, such as diazomethane and cyanide; (2) it has the potential for multigram scale-up following optimization. The laboratory scale production (up to 20 g) of the key intermediate, methyl 2-methoxy-2-alkenoate, was completed with good yield; (3) it is highly suitable for synthesizing a wide range of β^3^-amino acids with unnatural side chains; (4) the method operates under mild reaction conditions and achieves good overall yields, making it economical, practical, and reliable; (5) in terms of time efficiency, over 1 g of chiral N-Boc-β^3^-amino acid methyl ester was prepared from α-amino acid in a single batch within 3 days. The synthesis of homoprolinol and homopipecolinol represents a formal approach to the total synthesis of sedum alkaloids. This approach provides a viable alternative to the hazardous Arndt–Eistert homologation and cyanation reaction. Further work is in progress.

## Data availability

The authors declare that the data supporting the findings of this study are available within the paper and its ESI.† Should any raw data files be needed in another format they are available from the corresponding author upon reasonable request. Source data are provided with this paper.

## Conflicts of interest

There are no conflicts to declare.

## Supplementary Material

RA-014-D4RA07506D-s001
