# Correction: A safe and efficient synthesis of N-Boc-β^3^-amino acid methyl esters from α-amino acids: applications in the formal synthesis of sedum alkaloids

**DOI:** 10.1039/d4ra90138j

**Published:** 2024-11-20

**Authors:** Bohua Long, Lijie Ren, Mengmeng Jiang, Shengquan Hu, Qianqian Jiang, Limin Li, Xuanluan Chen, Zhengzhi Wu

**Affiliations:** a Department of Neurology, Shenzhen Second People's Hospital, The First Affiliated Hospital of Shenzhen University Health Science Center Shenzhen 518035 China bhlong121@163.com renlijie72@126.com szwzz001@163.com; b Wu Zhengzhi Academician Workstation, Ningbo College of Health Sciences Ningbo 315800 People's Republic of China; c Shenzhen Institute of Geriatric Medicine Shenzhen 518035 China

## Abstract

Correction for ‘A safe and efficient synthesis of N-Boc-β^3^-amino acid methyl esters from α-amino acids: applications in the formal synthesis of sedum alkaloids’ by Bohua Long *et al.*, *RSC Adv.*, 2024, **14**, 36016–36021, https://doi.org/10.1039/D4RA07506D.

The authors regret that the one of the affiliations (affiliation *a*) was incorrectly shown in the original manuscript. The corrected list of affiliations is as shown above.

The Royal Society of Chemistry apologises for these errors and any consequent inconvenience to authors and readers.

