# Supplementary material for: A safe and efficient synthesis of N-Boc-β3-amino acid methyl esters from α-amino acids: applications in the formal synthesis of sedum alkaloids
Source: RSC Adv. 2024 Nov 11;14(48):36016–21. doi: 10.1039/d4ra07506d (PMC11552086; doi:10.1039/d4ra07506d)
Supplement: RA-014-D4RA07506D-s001 [file RA-014-D4RA07506D-s001.pdf]

Supplementary Information (SI) for RSC Advances.
This journal is © The Royal Society of Chemistry 2024

A safe and efficient route for preparation of *N*-Boc-β^3-amino acid methyl esters from α-amino acids and application to the formal syntheses of sedum alkaloids

Content

General Experimental page 2

Experimental Procedures page 3

NMR Spectra of New Compounds and Selected Known Compounds page 23

General Experimental

Commercially available reagents were used without further purification unless otherwise stated. All solvents were distilled prior to use: toluene, benzene, diethyl ether and tetrahydrofuran were distilled from Na/benzophenone; while dichloromethane, dimethylformamide, acetonitrile, triethylamine and diisopropylethylamine were distilled from CaH_2. Methanol was distilled under a N_2 atmosphere from Mg/I_2. All reactions were conducted in oven-dried (120 °C) or flame-dried glasswares under a N_2 atmosphere, and at ambient temperature (20 to 25 °C) unless otherwise stated. All non-aqueous reactions were performed by standard syringe in septa techniques. Evaporation and concentration under reduced pressure was performed at 50-500 mbar. ^{1}H NMR spectra were recorded in $CDCl_3$ (unless stated otherwise) on a Bruker Avance AV600 or 400 at 600 MHz (150 MHz) or 400 MHz (100 MHz), respectively. Chemical shifts are reported as δ values (ppm) referenced to either a tetramethylsilane (TMS) internal standard or the signals due to the solvent residual. Data for ^{1}H NMR are reported as follows: chemical shift (δ ppm), multiplicity (s = singlet, brs = broad singlet, d = doublet, t = triplet, q = quartet, m = multiplet), coupling constant (Hz), integration. Some peptide intermediates exist as rotational conformers, the chemical shift for the minor isomers were indicated using parentheses next to the peak for their major isomers. Mass spectra were measured on ABI Q-star Elite. Optical rotations were measured on a Perkin-Elmer 351 polarimeter at 589 nm with a 100 mm path length cell at 20 °C (reported as follows: concentration (*c* in g/100 mL), solvent). The reaction progresses were checked on pre-coated thin layer chromatography (TLC) plates. TLC was carried out using pre-coated sheets (Qingdao silica gel 60-F250, 0.2 mm) which, after development, were visualized under UV light at 254nm. Flash column chromatography was performed using the indicated solvents on E. Qingdao silica gel 60 (230-400 mesh ASTM). Yields refer to chromatographically purified compounds, unless otherwise stated.

Experimental procedures

General Procedure A (GPA):

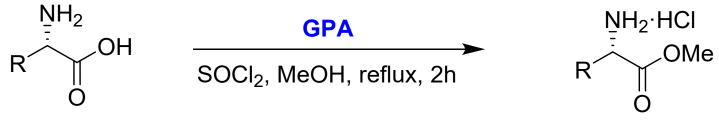

α-amino acid **α-amino acid methyl ester hydrochloride**

To a solution of **α-amino acid** (1.0 *eq.*) in MeOH was dropwise added $SOCl_2$ (3.0 *eq.*) at 0 °C. After 30 min, the resultant mixture was heated to reflux and stirred for 2h. The solution was cooled to room temperature and volatiles of the reaction mixture were removed in vacuo to obtain the **α-amino acid methyl ester hydrochloride**, which was used for next step directly.

General Procedure B (GPB):

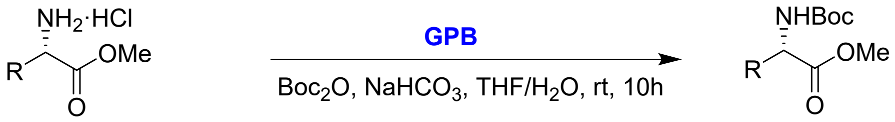

α-amino acid methyl ester hydrochloride **N-Boc-α-amino acid methyl ester**

To a solution of the above-obtained **α-amino acid methyl ester hydrochloride** (1.0 *eq.*) in THF/H_2O (1:1) was added $NaHCO_3$ (3.0 *eq.*) and Boc_2O (1.0 *eq.*) at 0 °C. After being stirred at room temperature for 10 h, volatiles of the reaction mixture were removed in vacuo. The solution was then diluted with water. The aqueous phase was extracted with ethyl acetate for 3 times. The combined organic phase was washed by brine, dried over sodium sulfate (anhydrous) and concentrated in *vacuo* to give the **N-Boc-α-amino acid methyl ester**, which was used for next step directly.

General Procedure C (GPC):

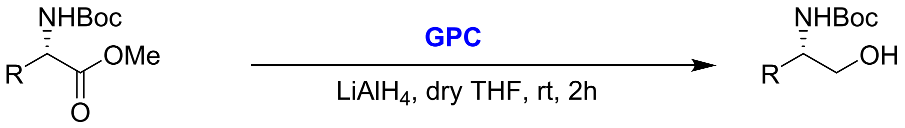

N-Boc-α-amino acid methyl ester **N-Boc-α-amino alcohol**

To a solution of the above-obtained **N-Boc-α-amino acid methyl ester** (1.0 *eq.*) in dry THF was added $LiAlH_4$ (1.5 *eq.*) at temperatures below -50 °C. After 30 min, the resultant mixture was allowed to warm to room temperature slowly and stirred for 2h. The reaction was then quenched with saturated aqueous Na_2SO_4 solution at temperatures below -50 °C. Volatiles of the reaction

mixture were removed in vacuo. The solution was then diluted with water. The aqueous phase was extracted with ethyl acetate for 3 times. The combined organic phase was washed by brine, dried over sodium sulfate (anhydrous) and concentrated in *vacuo* to give the **N-Boc-α-amino alcohol**, which was used for next step directly.

General Procedure D (GPD):

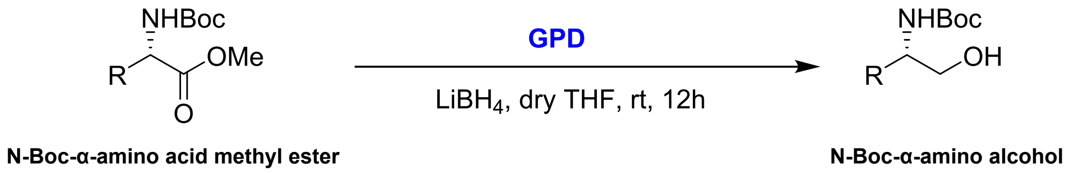

To a solution of the above-obtained **N-Boc-α-amino acid methyl ester** (1.0 *eq.*) in dry THF was added $LiBH_4$ (2.0 *eq.* — 5.0 *eq.*) at 0 °C. After 30 min, the resultant mixture was allowed to warm to room temperature slowly and stirred for 12h. The reaction was then quenched with saturated aqueous Na_2SO_4 solution at 0 °C. Volatiles of the reaction mixture were removed in vacuo. The solution was then diluted with water. The aqueous phase was extracted with ethyl acetate for 3 times. The combined organic phase was washed by brine, dried over sodium sulfate (anhydrous) and concentrated in *vacuo* to give the **N-Boc-α-amino alcohol**, which was used for next step directly.

General Procedure E (GPE):

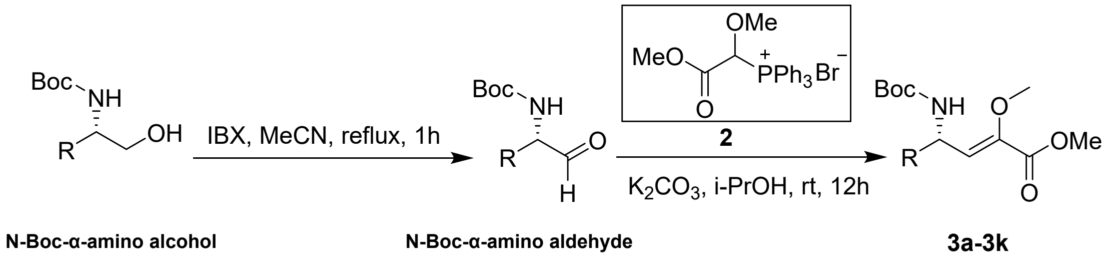

To a solution of the above-obtained **N-Boc-α-amino alcohol** (1.0 *eq.*) in dry MeCN was added IBX (1.2 *eq.*) at room temperature. After 15 min, the resultant mixture was heated to reflux and stirred for 1h. The solution was cooled to room temperature and the solid was removed by filtration through a pad of celite and washed with MeCN. The total filtrate was concentrated in vacuo to afford the **N-Boc-α-amino aldehyde**, which was used immediately for next step directly.

To a solution of the above-obtained **N-Boc-α-amino aldehyde** in i-PrOH was added

phosphonium reagent **2** [1] (1.2 *eq.*) and K_2CO_3 (1.2 *eq.*) at room temperature. The resultant mixture was stirred at room temperature for 12h. The solid was removed by filtration through a pad of celite and washed with EtOAc. The total filtrate was concentrated in *vacuo,* then purified by silica gel column chromatography (EA/PE, 1:10) to afford the methyl 2-methoxy-2-alkenoates **3a-3k**.

General Procedure F (GPF):

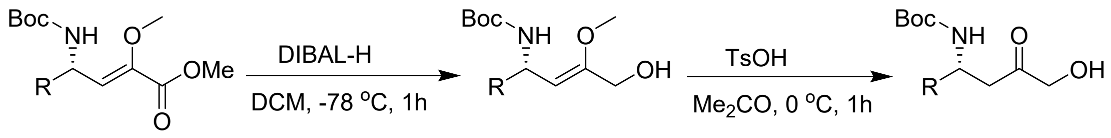

3a-3k **4a-4k** **5a-5k**

To a solution of the above-obtained **3a-3k** (1.0 *eq.*) in dry DCM was added DIBAL-H (2.0 *eq.*) at -78 °C. The resultant mixture was stirred at -78 °C for 1h. The reaction was then quenched with saturated aqueous Na_2SO_4 solution at -78 °C. Volatiles of the reaction mixture were removed in vacuo. The solution was then diluted with water. The aqueous phase was extracted with ethyl acetate for 3 times. The combined organic phase was washed by brine, dried over sodium sulfate (anhydrous) and concentrated in *vacuo* to give **4a-4k**, which was used immediately for next step directly.

To a solution of the above-obtained **4a-4k** in acetone was added TsOH (1.0 *eq.*) at 0 °C. The resultant mixture was stirred at 0 °C for 1h. The reaction was then quenched with Et_3N (2.0 eq.), then concentrated in *vacuo,* then purified by silica gel column chromatography (EA/PE, 1:2) to afford **5a-5k**.

General Procedure G (GPG):

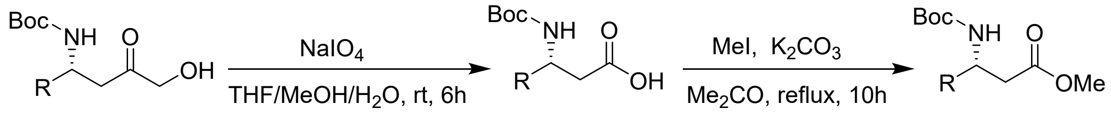

5a-5k **6a-6k** **7a-7k**

To a solution of the above-obtained **5a-5k** (1.0 *eq.*) in THF/MeOH/H_2O (1:1:1) was added $NaIO_4$ (4.0 *eq.*) at room temperature. The resultant mixture was stirred at room temperature for 6h. The reaction was then quenched with saturated aqueous Na_2SO_3 solution. Volatiles of the reaction mixture were removed in vacuo. The solution was then diluted with water and adjusted to pH = 4

by addition of solid $KHSO_4$. The aqueous phase was extracted with ethyl acetate for 3 times. The combined organic phase was washed by brine, dried over sodium sulfate (anhydrous) and concentrated in *vacuo* to give the acid **6a-6k**, which was used immediately for next step directly.

To a solution of the above-obtained acid **6a-6k** in dry acetone was added K_2CO_3 (3.0 *eq.*) and MeI (1.5 *eq.*). After 15 min, the resultant mixture was heated to reflux and stirred for 10h. The solution was cooled to room temperature and the solid was removed by filtration through a pad of celite and washed with EtOAc. The total filtrate was concentrated in vacuo, then purified by silica gel column chromatography (EA/PE, 1:4) to afford **7a-7k**.

Methyl (S)-3-((tert-butoxycarbonyl)amino)-4-phenylbutanoate (7a)

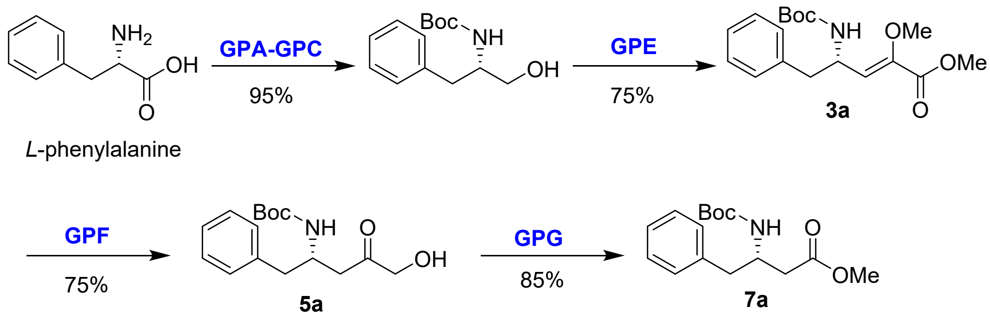

Compound **3a** :

$[\alpha]_D^{25}$ +27.1 (c 0.51, EtOAc); ^1H NMR (600 MHz, $CDCl_3$) δ 7.32 – 7.20 (m, 5H), 6.11 (d, *J* = 8.6 Hz, 1H), 4.80 (d, *J* = 14.6 Hz, 2H), 3.79 (s, 3H), 3.56 (s, 3H), 2.97 – 2.82 (m, 2H), 1.42 (s, 9H); ^{13}C NMR (151 MHz, $CDCl_3$) δ 199.38, 163.81, 154.92, 146.00, 137.02, 129.53, 129.31, 128.41, 126.80, 126.65, 79.51, 59.88, 52.04, 48.30, 41.04, 28.33; HR-ESIMS m/z: calculated for $C_{18}H_{25}NO_5Na^+$ $[M+Na]^+$: 358.1625, found 358.1630.

Compound **5a** :

$[\alpha]_D^{25}$ -20.0 (c 0.59 , EtOAc); ^1H NMR (600 MHz, $CDCl_3$) δ 7.27 (ddd, *J* = 58.0, 32.0, 6.4 Hz, 5H), 4.96 (d, *J* = 6.4 Hz, 1H), 4.27 – 4.12 (m, 3H), 3.14 (s, 1H), 2.98 (dd, *J* = 12.0, 5.7 Hz, 1H), 2.87 – 2.81 (m, 1H), 2.61 (d, *J* = 4.9 Hz, 2H), 1.42 (s, 9H); ^{13}C NMR (151 MHz, $CDCl_3$) δ 208.71, 155.20, 137.48, 129.18, 128.64, 126.77, 79.68, 68.59, 48.63, 41.63, 40.34, 28.27; HR-ESIMS m/z: calculated for $C_{16}H_{23}NO_4Na^+$ $[M+Na]^+$: 316.1519, found 316.1525.

Compound **7a** :

$[\alpha]_D^{25}$ -17.4 (c 0.46, EtOAc); ^1H NMR (600 MHz, $CDCl_3$) δ 7.33 – 7.19 (m, 5H), 5.07 (d, *J* = 6.6

Hz, 1H), 4.18 (s, 1H), 3.71 (s, 3H), 2.96 (dd, J = 12.8, 5.7 Hz, 1H), 2.83 (dd, J = 13.4, 7.8 Hz, 1H), 2.50 (ddd, J = 42.2, 15.9, 5.6 Hz, 2H), 1.43 (s, 9H); ^{13}C NMR (151 MHz, $CDCl_3$) δ 172.12, 155.11, 137.68, 129.35, 128.49, 126.57, 79.35, 77.21, 77.00, 76.79, 51.67, 48.75, 40.34, 37.50, 28.32; HR-ESIMS m/z: calculated for $C_{16}H_{23}NO_4Na^+$ [M+Na]$^+$: 316.1519, found 316.1524.

Methyl (S)-3-((tert-butoxycarbonyl)amino)hexanoate (7b)

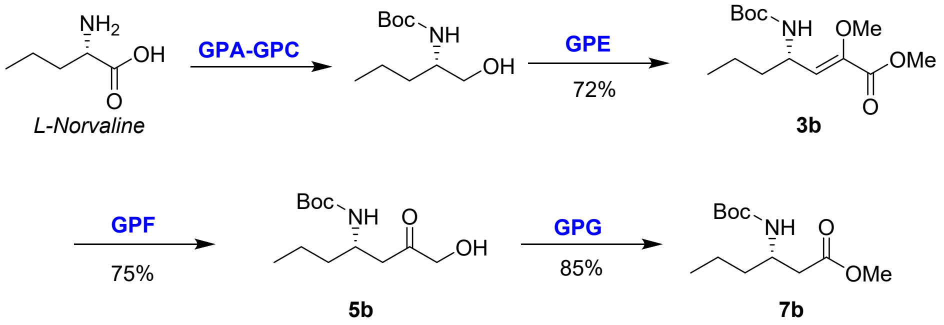

Compound **3b** :

$[\alpha]_D^{25}$ -1.3 (c 0.98, EtOAc); ^{1}H NMR (600 MHz, $CDCl_3$) δ 6.04 (d, J = 8.8 Hz, 1H), 4.66 (s, 1H), 4.53 (s, 1H), 3.77 (s, 3H), 3.72 (s, 3H), 1.59 – 1.47 (m, 2H), 1.42 (s, 9H), 1.37 – 1.31 (m, 2H), 0.91 (t, J = 7.3 Hz, 3H); ^{13}C NMR (151 MHz, $CDCl_3$) δ 164.04, 154.84, 145.96, 128.33, 79.31, 60.16, 52.00, 46.88, 37.36, 28.38, 18.90, 13.77; HR-ESIMS m/z: calculated for $C_{14}H_{25}NO_5Na^+$ [M+Na]$^+$: 310.1625, found 310.1629.

Compound **5b** :

$[\alpha]_D^{25}$ -20.2 (c 0.41 , EtOAc); ^{1}H NMR (600 MHz, $CDCl_3$) δ 4.74 (d, J = 6.7 Hz, 1H), 4.23 (q, J = 19.0 Hz, 2H), 4.01 – 3.88 (m, 1H), 3.13 (s, 1H), 2.62 (s, 2H), 1.72 (s, 1H), 1.55 – 1.46 (m, 2H), 1.41 (d, J = 11.7 Hz, 9H), 1.38 – 1.27 (m, 2H), 0.91 (t, J = 7.3 Hz, 3H); ^{13}C NMR (151 MHz, $CDCl_3$) δ 208.68, 155.43, 79.56, 68.70, 47.46, 43.60, 36.94, 28.31, 19.36, 13.71; HR-ESIMS m/z: calculated for $C_{12}H_{23}NO_4Na^+$ [M+Na]$^+$: 268.1519, found 268.1522.

Compound **7b** :

$[\alpha]_D^{25}$ -15.8 (c 0.53, EtOAc); ^{1}H NMR (600 MHz, $CDCl_3$) δ 4.90 (d, J = 7.7 Hz, 1H), 3.90 (d, J = 4.9 Hz, 1H), 3.67 (s, 3H), 2.50 (qd, J = 15.4, 5.3 Hz, 2H), 1.47 (dd, J = 12.6, 6.1 Hz, 2H), 1.42 (s, 9H), 1.38 – 1.30 (m, 2H), 0.90 (t, J = 7.3 Hz, 3H); ^{13}C NMR (151 MHz, $CDCl_3$) δ 172.18, 155.36, 79.14, 51.57, 47.34, 39.16, 36.74, 28.35, 19.33, 13.77; HR-ESIMS m/z: calculated for $C_{12}H_{23}NO_4Na^+$ [M+Na]$^+$: 268.1519, found 268.1524.

Methyl (3R,4S)-3-((tert-butoxycarbonyl)amino)-4-methylhexanoate (7c)

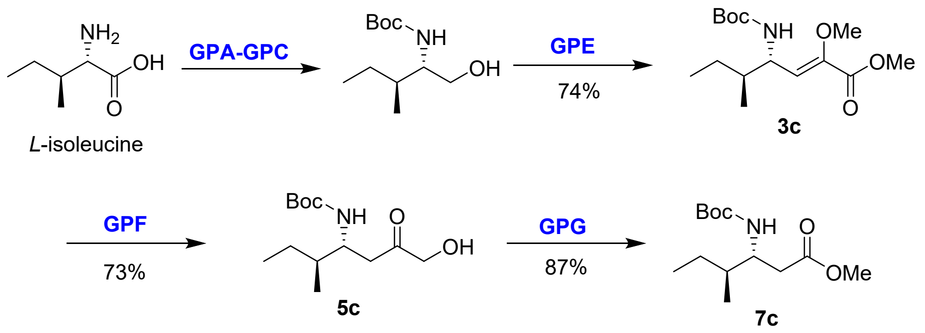

Compound **3c** :

$[\alpha]_D^{25}$ -2.1 (c 0.66 , EtOAc); ^{1}H NMR (600 MHz, $CDCl_3$) δ 6.04 (d, *J* = 9.1 Hz, 1H), 4.75 (s, 1H), 4.50 (d, *J* = 6.1 Hz, 1H), 3.78 (s, 3H), 3.72 (s, 3H), 1.57 (s, 1H), 1.50 – 1.46 (m, 1H), 1.43 (d, *J* = 9.7 Hz, 9H), 1.15 – 1.07 (m, 1H), 0.91 (t, *J* = 7.4 Hz, 3H), 0.87 (d, *J* = 6.8 Hz, 3H); ^{13}C NMR (151 MHz, $CDCl_3$) δ 164.03, 155.11, 146.51, 126.07, 79.28, 60.15, 52.03, 51.06, 39.44, 28.39, 25.56, 14.93, 11.54; HR-ESIMS m/z: calculated for $C_{15}H_{27}NO_5Na^+$ $[M+Na]^+$: 324.1781, found 324.1785.

Compound **5c** :

$[\alpha]_D^{25}$ -12.1 (c 0.57, EtOAc); ^{1}H NMR (600 MHz, $CDCl_3$) δ 4.78 (d, *J* = 8.3 Hz, 1H), 4.24 (dd, *J* = 61.5, 18.9 Hz, 2H), 3.89 – 3.81 (m, 1H), 2.63 – 2.49 (m, 2H), 1.57 (d, *J* = 9.3 Hz, 1H), 1.46 (s, 1H), 1.40 (s, 9H), 1.09 (dt, *J* = 14.6, 7.7 Hz, 1H), 0.89 (t, *J* = 7.4 Hz, 3H), 0.85 (d, *J* = 6.8 Hz, 3H); ^{13}C NMR (151 MHz, $CDCl_3$) δ 209.01, 155.52, 79.52, 77.21, 77.00, 76.79, 68.47, 51.73, 40.87, 38.59, 38.44, 28.26, 25.34, 15.33, 11.39; HR-ESIMS m/z: calculated for $C_{13}H_{25}NO_4Na^+$ $[M+Na]^+$: 282.1676, found 282.1679.

Compound **7c** :

$[\alpha]_D^{25}$ -10.6 (c 0.98 , EtOAc); ^{1}H NMR (600 MHz, $CDCl_3$) δ 4.88 (d, *J* = 8.6 Hz, 1H), 3.83 (dd, *J* = 12.2, 5.4 Hz, 1H), 3.67 (s, 3H), 2.47 (ddd, *J* = 22.4, 15.2, 5.8 Hz, 2H), 1.60 – 1.54 (m, 1H), 1.50 (dd, *J* = 15.7, 9.3 Hz, 1H), 1.46 – 1.40 (m, 9H), 1.15 – 1.07 (m, 1H), 0.90 (t, *J* = 7.4 Hz, 3H), 0.86 (d, *J* = 6.8 Hz, 3H); ^{13}C NMR (151 MHz, $CDCl_3$) δ 172.48, 155.42, 79.14, 77.21, 77.00, 76.79, 51.88, 51.68, 38.28, 36.59, 28.36, 25.43, 15.27, 11.44; HR-ESIMS m/z: calculated for $C_{13}H_{25}NO_4Na^+$ $[M+Na]^+$: 282.1676, found 282.1681.

Methyl (S)-3-((tert-butoxycarbonyl)amino)-4-(4-((triisopropylsilyl)oxy)phenyl)butanoate (7d)

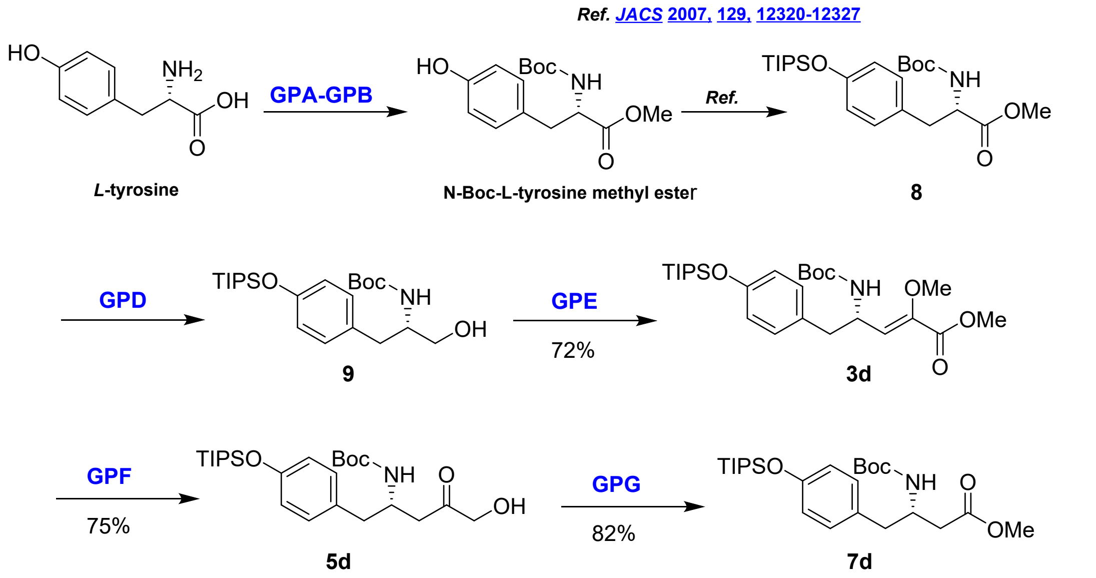

Compound **3d** :

$[\alpha]_D^{25}$ +23.2 (c 1.0, EtOAc); ^{1}H NMR (400 MHz, $CDCl_3$) δ 7.02 (d, *J* = 8.3 Hz, 2H), 6.80 (d, *J* = 8.3 Hz, 2H), 6.06 (d, *J* = 8.3 Hz, 1H), 4.71 (s, 2H), 3.76 (s, 3H), 3.55 (s, 3H), 2.80 (qd, *J* = 13.6, 6.3 Hz, 2H), 1.40 (s, 9H), 1.26 – 1.19 (m, 3H), 1.09 (m, 18H); ^{13}C NMR (101 MHz, $CDCl_3$) δ 163.85, 154.95, 154.84, 145.93, 130.38, 130.15, 129.35, 127.18, 119.82, 107.74, 79.81, 59.95, 51.98, 48.45, 40.27, 28.35, 17.88, 12.63; HR-ESIMS m/z: calculated for $C_{27}H_{45}NO_6SiNa^+$ $[M+Na]^+$: 530.2908, found 530.2915.

Compound **5d** :

$[\alpha]_D^{25}$ -8.0 (c 0.40 EtOAc); ^{1}H NMR (400 MHz, $CDCl_3$) δ 6.98 (d, *J* = 8.3 Hz, 2H), 6.80 (d, *J* = 8.4 Hz, 2H), 4.85 (d, *J* = 15.3 Hz, 2H), 4.14 (t, *J* = 12.2 Hz, 2H), 3.10 (s, 1H), 2.86 (dd, *J* = 13.3, 6.6 Hz, 1H), 2.73 (dd, *J* = 13.5, 7.8 Hz, 1H), 2.56 (d, *J* = 5.0 Hz, 2H), 1.40 (s, 9H), 1.27 – 1.20 (m, 3H), 1.10 – 1.05 (m, 18H); ^{13}C NMR (101 MHz, $CDCl_3$) δ 208.76, 155.25, 154.93, 130.05, 120.07, 103.90, 79.66, 68.56, 48.80, 41.71, 39.56, 28.30, 17.86, 12.61; HR-ESIMS m/z: calculated for $C_{25}H_{43}NO_5SiNa^+$ $[M+Na]^+$: 488.2803, found 488.2808.

Compound **7d** :

$[\alpha]_D^{25}$ -13.8 (c 0.81, EtOAc); ^{1}H NMR (400 MHz, $CDCl_3$) δ 7.01 (d, *J* = 8.3 Hz, 2H), 6.80 (d, *J* = 8.4 Hz, 2H), 4.97 (s, 1H), 4.09 (s, 1H), 3.68 (s, 3H), 2.84 (dd, *J* = 13.5, 6.2 Hz, 1H), 2.73 (dd, *J* = 13.6, 7.7 Hz, 1H), 2.45 (qd, *J* = 15.8, 5.6 Hz, 2H), 1.41 (s, 9H), 1.23 (dd, *J* = 14.9, 7.1 Hz, 3H), 1.09 (d, *J* = 7.2 Hz, 18H); ^{13}C NMR (101 MHz, $CDCl_3$) δ 171.83, 155.13, 154.76, 130.20, 119.89, 79.29,

51.62, 48.89, 39.53, 37.55, 28.35, 17.89, 12.64; HR-ESIMS m/z: calculated for $C_{25}H_{43}NO_5SiNa^+$ $[M+Na]^+$: 488.2803, found 488.2808.

Methyl (R)-3-((tert-butoxycarbonyl)amino)-4-((tert-butyldiphenylsilyl)oxy)butanoate (7e)

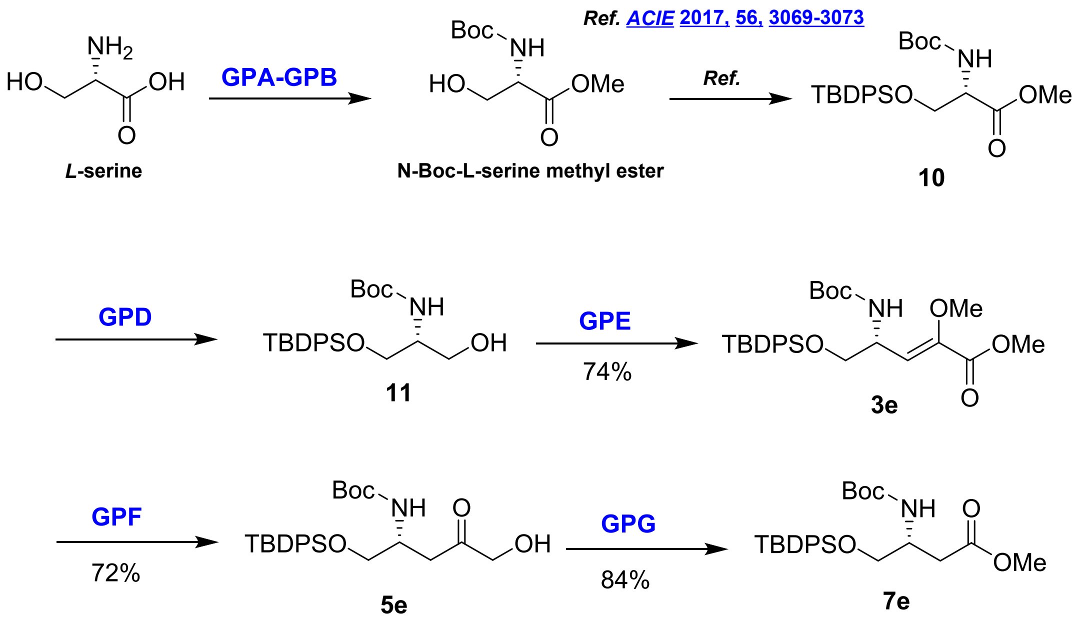

Compound **3e** :

$[\alpha]_D^{25}$ +1.4 (c 0.50 EtOAc); ^{1}H NMR (400 MHz, $CDCl_3$) δ 7.64 (dd, J = 6.2, 5.6 Hz, 4H), 7.44 – 7.35 (m, 6H), 6.21 (d, J = 8.4 Hz, 1H), 5.05 (s, 1H), 4.69 (s, 1H), 3.78 (s, 3H), 3.63 (s, 3H), 1.45 (s, 9H), 1.07 (s, 9H); ^{13}C NMR (101 MHz, $CDCl_3$) δ 163.68, 155.15, 146.20, 141.79, 135.57, 135.52, 129.80, 127.73, 127.70, 107.34, 94.18, 79.45, 65.84, 60.01, 51.97, 48.87, 28.36, 26.81, 26.53, 19.25; HR-ESIMS m/z: calculated for $C_{28}H_{39}NO_6SiNa^+$ $[M+Na]^+$: 536.2439, found 436.2445.

Compound **5e** :

$[\alpha]_D^{25}$ +14.6 (c 0.26, EtOAc); ^{1}H NMR (400 MHz, $CDCl_3$) δ 7.69 – 7.50 (m, 4H), 7.44 – 7.37 (m, 6H), 4.97 (s, 1H), 4.19 (s, 2H), 4.11 (s, 1H), 3.71 (d, J = 4.1 Hz, 2H), 2.68 (dt, J = 15.9, 8.1 Hz, 2H), 1.42 (s, 9H), 1.06 (s, 9H); ^{13}C NMR (101 MHz, $CDCl_3$) δ 208.30, 155.07, 135.50, 132.81, 129.96, 127.85, 79.80, 68.44, 65.04, 48.61, 40.01, 28.31, 26.88, 19.26; HR-ESIMS m/z: calculated for $C_{26}H_{37}NO_5SiNa^+$ $[M+Na]^+$: 494.2333, found 494.2339.

Compound **7e** :

$[\alpha]_D^{25}$ +15.7 (c 0.97, EtOAc); ^{1}H NMR (400 MHz, $CDCl_3$) δ 7.62 (d, J = 6.9 Hz, 4H), 7.45 – 7.35 (m, 6H), 5.05 (s, 1H), 4.11 (s, 1H), 3.70 (s, 2H), 3.64 (s, 3H), 2.64 (d, J = 4.8 Hz, 2H), 1.43 (s, 9H),

1.06 (s, 9H); ^{13}C NMR (101 MHz, $CDCl_3$) δ 171.90, 154.83, 135.53, 133.01, 129.80, 127.75, 79.29, 77.32, 77.00, 76.68, 64.93, 51.63, 48.85, 35.91, 28.35, 26.83, 19.27; HR-ESIMS m/z: calculated for $C_{26}H_{37}NO_5SiNa^+$ [M+Na]$^+$: 494.2333, found 494.2338.

Methyl (S)-3-((tert-butoxycarbonyl)amino)-4-(1-tosyl-1H-indol-3-yl)butanoate (7f)

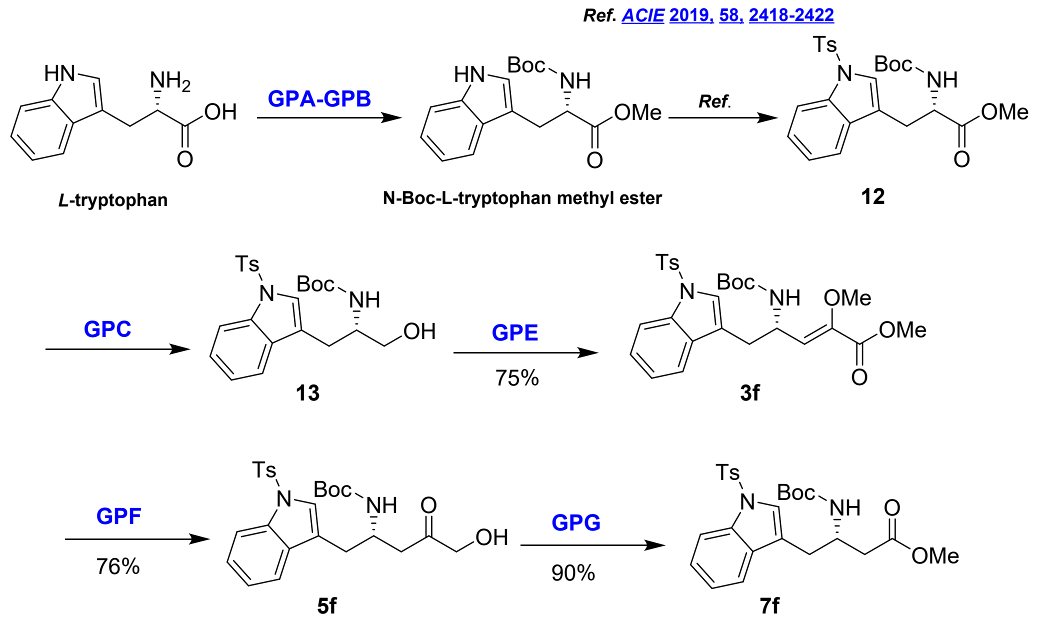

Compound **3f** :

$[\alpha]_D^{25}$ -9.2 (c 1.15, EtOAc); ^{1}H NMR (400 MHz, $CDCl_3$) δ 7.97 (d, *J* = 8.2 Hz, 1H), 7.75 (d, *J* = 8.3 Hz, 2H), 7.56 (d, *J* = 7.7 Hz, 1H), 7.43 (s, 1H), 7.27 (ddd, *J* = 24.8, 16.1, 7.9 Hz, 4H), 6.09 (d, *J* = 8.4 Hz, 1H), 4.87 (d, *J* = 7.4 Hz, 2H), 3.79 (s, 3H), 3.46 (s, 3H), 3.09 – 2.88 (m, 2H), 2.34 (s, 3H), 1.44 (s, 9H); ^{13}C NMR (101 MHz, $CDCl_3$) δ 163.52, 154.89, 144.76, 135.13, 135.06, 130.88, 129.77, 126.73, 126.29, 124.70, 124.22, 123.12, 119.69, 118.12, 113.56, 79.66, 59.69, 52.00, 46.94, 30.49, 28.28, 21.46; HR-ESIMS m/z: calculated for $C_{27}H_{32}N_2O_7SNa^+$ [M+Na]$^+$: 551.1822, found 5510.1829.

Compound **5f** :

$[\alpha]_D^{25}$ -15.2 (c 0.87, EtOAc); ^{1}H NMR (400 MHz, $CDCl_3$) δ 8.01 (d, *J* = 8.2 Hz, 1H), 7.77 (d, *J* = 8.3 Hz, 2H), 7.56 (d, *J* = 7.8 Hz, 1H), 7.42 – 7.23 (m, 5H), 4.99 (d, *J* = 6.4 Hz, 1H), 4.22 – 4.11 (m, 2H), 3.09 – 2.92 (m, 2H), 2.64 (d, *J* = 2.5 Hz, 2H), 2.36 (s, 3H), 1.44 (s, 9H); ^{13}C NMR (101 MHz, $CDCl_3$) δ 208.55, 155.17, 144.92, 135.21, 135.15, 130.64, 129.83, 126.74, 124.93, 124.17, 123.32, 119.51, 118.61, 113.74, 79.86, 68.55, 47.18, 41.74, 29.69, 28.25, 21.50; HR-ESIMS m/z:

calculated for $C_{25}H_{30}N_2O_6SNa^+$ $[M+Na]^+$: 509.1717, found 509.1721.

Compound **7f** :

$[\alpha]_D^{25}$ -18.9 (c 0.97, EtOAc); ^{1}H NMR (400 MHz, $CDCl_3$) δ 7.97 (d, *J* = 8.2 Hz, 1H), 7.75 (d, *J* = 8.3 Hz, 2H), 7.56 (d, *J* = 7.7 Hz, 1H), 7.43 (s, 1H), 7.27 (ddd, *J* = 24.8, 16.1, 7.9 Hz, 4H), 6.09 (d, *J* = 8.4 Hz, 1H), 4.87 (d, *J* = 7.4 Hz, 2H), 3.79 (s, 3H), 3.46 (s, 3H), 3.09 – 2.88 (m, 2H), 2.34 (s, 3H), 1.44 (s, 9H); ^{13}C NMR (101 MHz, $CDCl_3$) δ 171.95, 155.11, 144.78, 135.23, 135.18, 130.83, 129.79, 126.74, 124.78, 124.27, 123.24, 119.68, 118.82, 113.69, 79.53, 51.69, 47.25, 37.47, 29.64, 28.29, 21.48; HR-ESIMS m/z: calculated for $C_{25}H_{30}N_2O_6SNa^+$ $[M+Na]^+$: 509.1717, found 509.1721.

Methyl (3R,4R)-3-((tert-butoxycarbonyl)amino)-4-((tert-butyldimethylsilyl)oxy)pentanoate (7g)

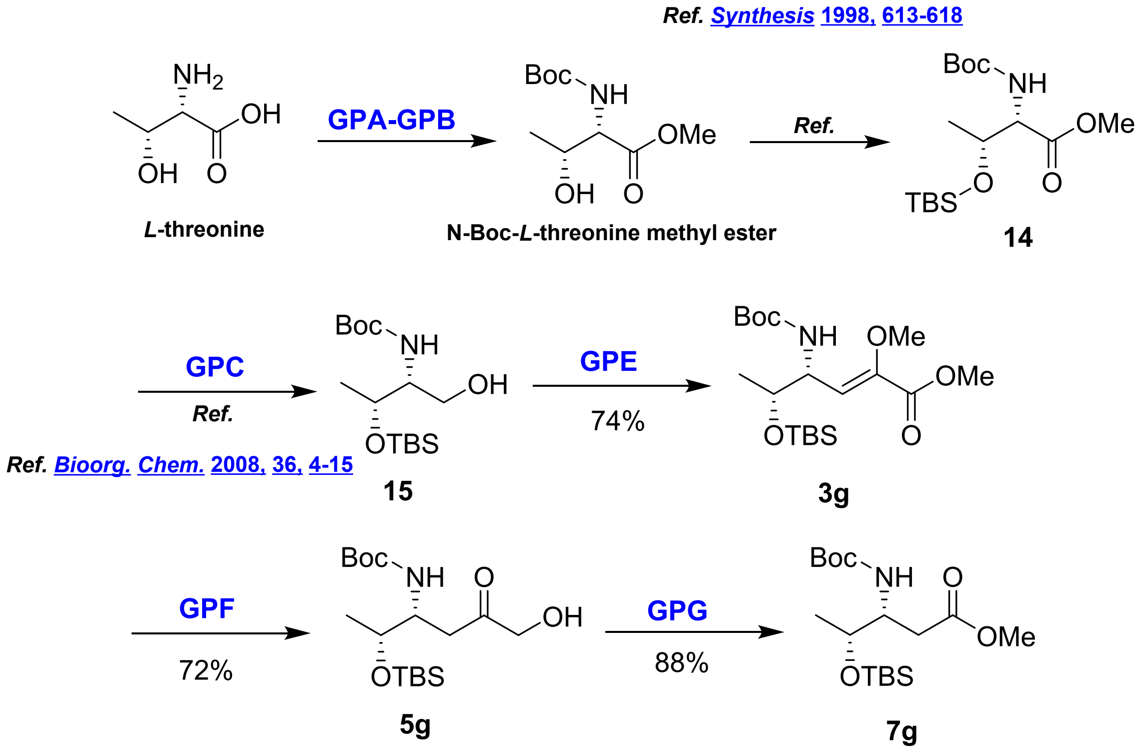

Compound **3g** :

$[\alpha]_D^{25}$ -7.40 (c 0.73, EtOAc); ^{1}H NMR (400 MHz, $CDCl_3$) δ 6.15 (d, *J* = 8.9 Hz, 1H), 4.98 (d, *J* = 7.0 Hz, 1H), 4.48 (s, 1H), 3.89 (d, *J* = 4.7 Hz, 1H), 3.77 (s, 3H), 3.73 (s, 3H), 1.44 (s, 9H), 1.17 (d, *J* = 6.2 Hz, 3H), 0.88 (s, 9H), 0.02 (d, *J* = 9.2 Hz, 6H); ^{13}C NMR (101 MHz, $CDCl_3$) δ 163.91, 155.56, 145.48, 127.92, 79.37, 70.61, 60.27, 52.39, 51.96, 28.39, 25.76, 20.78, 17.97, -4.50, -5.04; HR-ESIMS m/z: calculated for $C_{19}H_{37}NO_6SiNa^+$ $[M+Na]^+$: 426.2282, found 426.2286.

Compound **5g** :

$[\alpha]_D^{25}$ +0.42 (c 0.24, EtOAc); ^{1}H NMR (600 MHz, $CDCl_3$) δ 4.82 (d, *J* = 8.8 Hz, 1H), 4.25 (dd, *J* = 11.3, 4.3 Hz, 2H), 3.95 (dd, *J* = 13.9, 6.8 Hz, 2H), 3.13 (s, 1H), 2.61 (qd, *J* = 15.4, 6.7 Hz, 2H), 1.43 (s, 9H), 1.15 (d, *J* = 6.1 Hz, 3H), 0.89 (s, 9H), 0.05 (d, *J* = 18.9 Hz, 6H); ^{13}C NMR (151 MHz, $CDCl_3$) δ 208.15, 155.78, 79.73, 69.19, 68.42, 52.46, 41.86, 28.32, 25.82, 20.59, 17.98, -4.24, -4.93; HR-ESIMS m/z: calculated for $C_{17}H_{35}NO_5SiNa^+$ $[M+Na]^+$: 384.2177, found 384.2180.

Compound **7g** :

$[\alpha]_D^{25}$ +3.1 (c 0.29, EtOAc); ^{1}H NMR (600 MHz, $CDCl_3$) δ 4.81 (d, *J* = 9.1 Hz, 1H), 3.92 (dd, *J* = 14.6, 6.7 Hz, 2H), 3.66 (s, 3H), 2.50 (d, *J* = 7.0 Hz, 2H), 1.44 (s, 9H), 1.14 (d, *J* = 6.1 Hz, 3H), 0.89 (s, 9H), 0.05 (d, *J* = 10.6 Hz, 6H); ^{13}C NMR (151 MHz, $CDCl_3$) δ 171.93, 155.64, 79.34, 68.89, 52.83, 51.65, 37.51, 28.35, 25.82, 20.67, 17.97, -4.31, -5.07; HR-ESIMS m/z: calculated for $C_{17}H_{35}NO_5SiNa^+$ $[M+Na]^+$: 384.2177, found 384.2180.

Methyl (R)-3-((tert-butoxycarbonyl)amino)-3-(4-((triisopropylsilyl)oxy)phenyl)propanoate (7h)

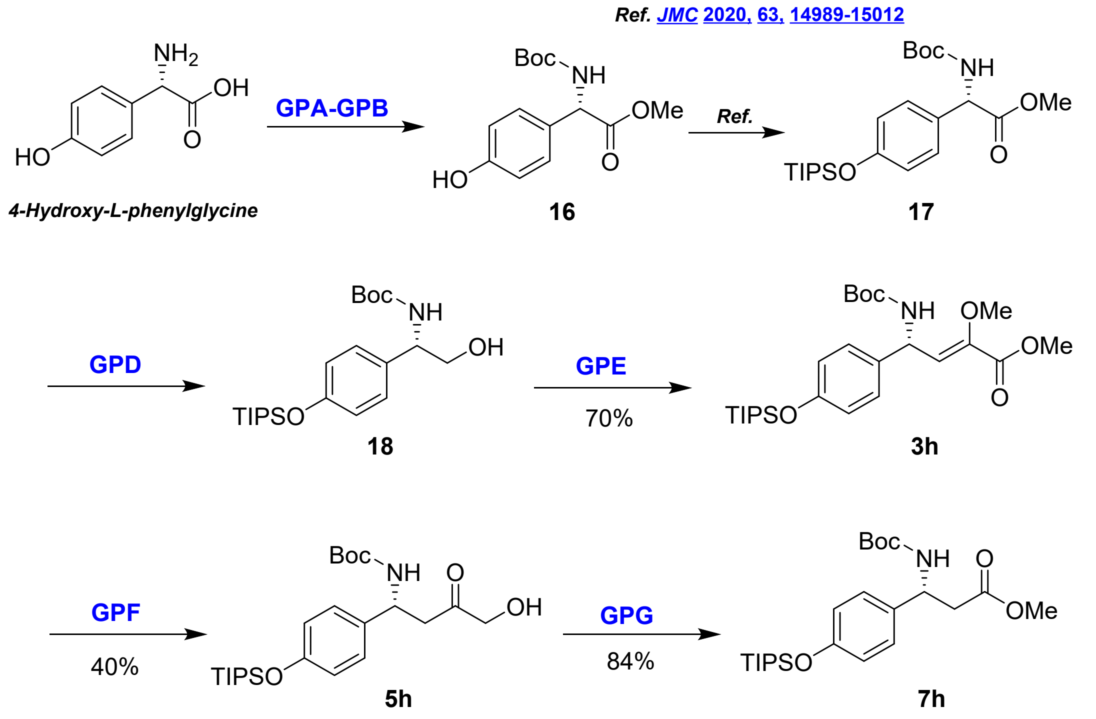

Compound **3h** :

$[\alpha]_D^{25}$ -18.1 (c 0.47, EtOAc); ^{1}H NMR (600 MHz, $CDCl_3$) δ 7.15 (d, *J* = 8.5 Hz, 2H), 6.85 – 6.80

(m, 2H), 6.32 (d, J = 8.5 Hz, 1H), 5.65 (s, 1H), 5.04 (s, 1H), 3.77 (s, 3H), 3.67 (s, 3H), 1.43 (s, 9H), 1.23 (dt, J = 14.9, 7.5 Hz, 3H), 1.08 (d, J = 7.4 Hz, 18H); ^{13}C NMR (151 MHz, $CDCl_3$) δ 163.95, 155.58, 154.86, 145.72, 132.87, 127.66, 127.42, 120.02, 79.69, 60.04, 52.05, 50.09, 28.35, 17.87, 12.61; HR-ESIMS m/z: calculated for $C_{26}H_{43}NO_6SiNa^+$ $[M+Na]^+$: 516.2752, found 516.2754.

Compound **5h** :

$[\alpha]_D^{25}$ +16.3 (c 0.46, EtOAc); ^{1}H NMR (600 MHz, $CDCl_3$) δ 7.15 (d, J = 8.5 Hz, 2H), 6.85 – 6.80 (m, 2H), 6.32 (d, J = 8.5 Hz, 1H), 5.65 (s, 1H), 5.04 (s, 1H), 3.77 (s, 3H), 3.67 (s, 3H), 1.43 (s, 9H), 1.23 (dt, J = 14.9, 7.5 Hz, 3H), 1.08 (d, J = 7.4 Hz, 18H); ^{13}C NMR (151 MHz, $CDCl_3$) δ 207.91, 155.64, 155.04, 132.94, 127.25, 120.13, 79.92, 68.81, 50.78, 44.92, 28.27, 17.84, 12.58; HR-ESIMS m/z: calculated for $C_{24}H_{41}NO_5SiNa^+$ $[M+Na]^+$: 474.2646, found 474.2649.

Compound **7h** :

$[\alpha]_D^{25}$ +20.3 (c 0.62, EtOAc); ^{1}H NMR (600 MHz, $CDCl_3$) δ 7.15 (d, J = 8.5 Hz, 2H), 6.85 – 6.80 (m, 2H), 6.32 (d, J = 8.5 Hz, 1H), 5.65 (s, 1H), 5.04 (s, 1H), 3.77 (s, 3H), 3.67 (s, 3H), 1.43 (s, 9H), 1.23 (dt, J = 14.9, 7.5 Hz, 3H), 1.08 (d, J = 7.4 Hz, 18H); ^{13}C NMR (151 MHz, $CDCl_3$) δ 171.43, 155.36, 154.99, 133.10, 127.19, 119.91, 79.57, 51.65, 50.72, 40.91, 28.29, 17.84, 12.58; HR-ESIMS m/z: calculated for $C_{24}H_{41}NO_5SiNa^+$ $[M+Na]^+$: 474.2646, found 474.2649.

Methyl (S)-3-((tert-butoxycarbonyl)amino)-5-((tert-butyldiphenylsilyl)oxy)pentanoate (7i)

Ref. ***Synthesis*** **1995, 810-814** ***Similar Ref.*** ***Tetrahedron*** **2005, 61, 10277-10284**

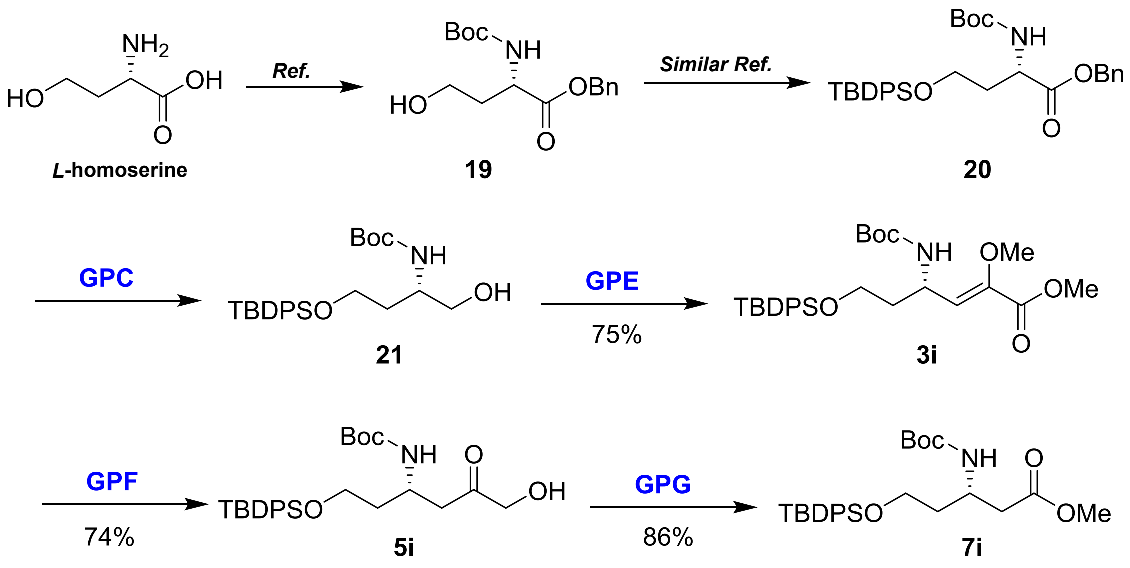

Compound **3i** :

$[\alpha]_D^{25}$ +3.5 (c 0.51, EtOAc); ^{1}H NMR (600 MHz, $CDCl_3$) δ 7.70 – 7.64 (m, 4H), 7.45 – 7.38 (m, 6H), 6.22 (d, *J* = 7.9 Hz, 1H), 5.72 (s, 1H), 4.74 (dd, *J* = 7.7, 4.6 Hz, 1H), 3.78 (s, 3H), 3.76 (s, 1H), 3.73 (s, 3H), 3.71 (s, 1H), 1.89 (s, 1H), 1.77 (s, 1H), 1.43 (s, 9H), 1.07 (s, 9H); ^{13}C NMR (151 MHz, $CDCl_3$) δ 163.88, 155.22, 145.40, 135.54, 133.03, 129.86, 129.77, 129.12, 127.79, 127.74, 79.06, 61.49, 59.99, 51.95, 46.25, 36.05, 28.42, 26.77, 19.03; HR-ESIMS m/z: calculated for $C_{29}H_{41}NO_6SiNa^+$ $[M+Na]^+$: 550.2595, found 550.2598.

Compound **5i** :

$[\alpha]_D^{25}$ -6.4 (c 0.33, EtOAc); ^{1}H NMR (600 MHz, $CDCl_3$) δ 7.69 – 7.58 (m, 4H), 7.42 (dt, *J* = 28.1, 7.3 Hz, 6H), 5.49 (d, *J* = 6.1 Hz, 1H), 4.20 (t, *J* = 4.3 Hz, 2H), 4.16 – 4.06 (m, 1H), 3.78 – 3.69 (m, 2H), 3.11 (t, *J* = 4.2 Hz, 1H), 2.79 (dd, *J* = 15.6, 5.2 Hz, 1H), 2.59 (dd, *J* = 15.6, 6.3 Hz, 1H), 1.85 (d, *J* = 3.9 Hz, 1H), 1.76 (d, *J* = 5.0 Hz, 1H), 1.41 (s, 9H), 1.06 (s, 9H); ^{13}C NMR (151 MHz, $CDCl_3$) δ 208.43, 155.45, 135.53, 135.51, 133.01, 129.83, 127.78, 79.38, 68.62, 61.29, 46.42, 43.11, 35.76, 28.32, 26.81, 19.03; HR-ESIMS m/z: calculated for $C_{27}H_{39}NO_5SiNa^+$ $[M+Na]^+$: 508.2490, found 508.2493.

Compound **7i** :

$[\alpha]_D^{25}$ -4.8 (c 0.60, EtOAc); ^{1}H NMR (600 MHz, $CDCl_3$) δ 7.70 – 7.62 (m, 4H), 7.41 (dt, *J* = 26.8, 7.4 Hz, 6H), 5.44 (d, *J* = 7.3 Hz, 1H), 4.16 – 4.06 (m, 1H), 3.80 – 3.75 (m, 1H), 3.70 (dt, *J* = 10.6, 5.2 Hz, 1H), 3.67 (d, *J* = 16.7 Hz, 3H), 2.71 (dd, *J* = 15.4, 4.6 Hz, 1H), 2.53 (dd, *J* = 15.5, 6.7 Hz, 1H), 1.86 (d, *J* = 5.3 Hz, 1H), 1.76 (dd, *J* = 12.8, 5.3 Hz, 1H), 1.42 (s, 9H), 1.05 (s, 9H); ^{13}C NMR (151 MHz, $CDCl_3$) δ 172.06, 155.26, 135.54, 135.53, 133.24, 129.71, 127.71, 79.06, 61.30, 51.57, 46.30, 38.85, 35.80, 28.36, 26.79, 19.05; HR-ESIMS m/z: calculated for $C_{27}H_{39}NO_5SiNa^+$ $[M+Na]^+$: 508.2490, found 508.2493.

Methyl (S)-3-((tert-butoxycarbonyl)amino)-6-((tert-butyldiphenylsilyl)oxy)hexanoate (7j)

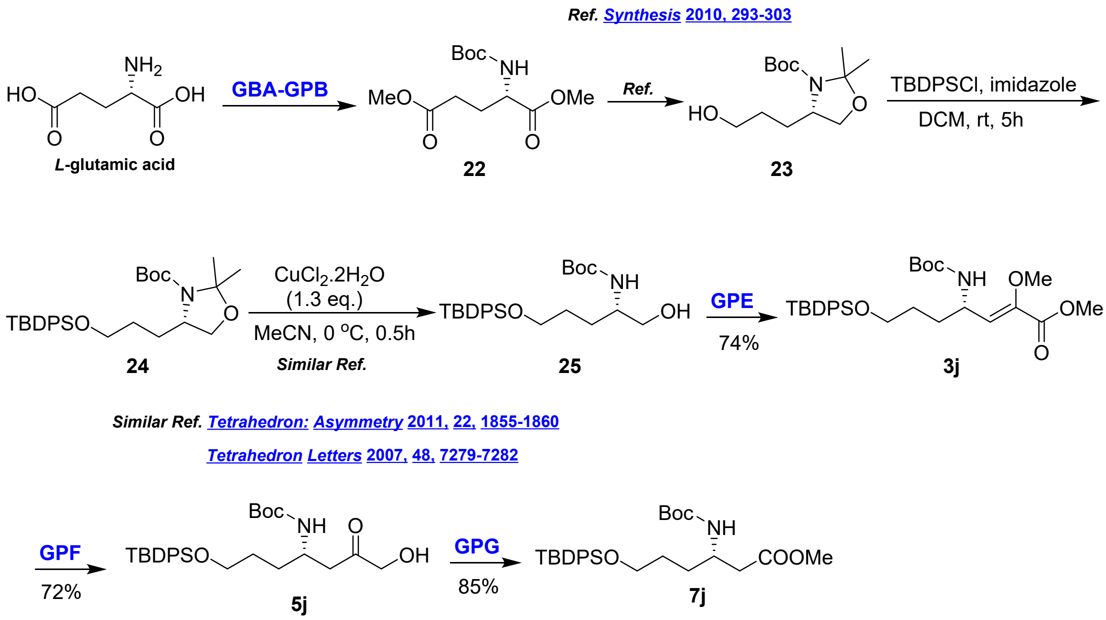

Compound **3j** :

$[\alpha]_D^{25}$ -0.91 (c 0.44, EtOAc); ^{1}H NMR (400 MHz, $CDCl_3$) δ 7.66 (d, *J* = 6.4 Hz, 4H), 7.42 – 7.38 (m, 6H), 6.08 (d, *J* = 8.7 Hz, 1H), 4.84 (s, 1H), 4.55 (s, 1H), 3.79 (s, 3H), 3.72 (s, 3H), 3.68 (d, *J* = 6.3 Hz, 2H), 1.68 – 1.57 (m, 4H), 1.44 (s, 9H), 1.06 (s, 9H); ^{13}C NMR (101 MHz, $CDCl_3$) δ 163.93, 155.05, 145.87, 135.47, 133.74, 129.53, 127.57, 79.26, 63.33, 60.06, 51.94, 46.98, 31.42, 28.59, 28.35, 26.81, 19.13; HR-ESIMS m/z: calculated for $C_{30}H_{43}NO_6SiNa^+$ $[M+Na]^+$: 564.2752, found 564.2759.

Compound **5j** :

$[\alpha]_D^{25}$ -10.3 (c 0.38, EtOAc); ^{1}H NMR (400 MHz, $CDCl_3$) δ 7.67 – 7.63 (m, 4H), 7.40 (ddd, *J* = 16.6, 9.6, 4.7 Hz, 6H), 4.85 (d, *J* = 7.6 Hz, 1H), 4.22 (q, *J* = 19.0 Hz, 2H), 3.91 (s, 1H), 3.67 (s, 2H), 3.15 (s, 1H), 2.61 (d, *J* = 5.4 Hz, 2H), 1.65-1.53 (m, 4H), 1.42 (s, 9H), 1.05 (s, 9H); ^{13}C NMR (101 MHz, $CDCl_3$) δ 208.53, 155.43, 135.50, 133.73, 129.61, 127.63, 79.55, 68.67, 63.46, 63.25, 47.59, 43.62, 31.04, 29.03, 28.30, 26.84, 19.15; HR-ESIMS m/z: calculated for $C_{28}H_{41}NO_5SiNa^+$ $[M+Na]^+$: 522.2646, found 522.2651.

Compound **7j** :

$[\alpha]_D^{25}$ -6.8 (c 0.41, EtOAc); ^{1}H NMR (400 MHz, $CDCl_3$) δ 7.65 (dd, *J* = 7.8, 1.5 Hz, 4H), 7.44 – 7.35 (m, 6H), 4.95 (d, *J* = 8.3 Hz, 1H), 3.92 (d, *J* = 17.4 Hz, 1H), 3.67 (s, 3H), 3.67 – 3.58 (m, 2H), 2.51 (d, *J* = 5.3 Hz, 2H), 1.64 – 1.53 (m, 4H), 1.43 (s, 9H), 1.04 (s, 9H); ^{13}C NMR (101 MHz, $CDCl_3$)

δ 172.06, 155.38, 135.52, 133.84, 129.56, 127.61, 79.20, 63.41, 51.60, 47.54, 39.26, 30.89, 29.13, 28.36, 26.84, 19.18; HR-ESIMS m/z: calculated for $C_{28}H_{41}NO_5SiNa^+$ $[M+Na]^+$: 522.2646, found 522.2651.

Methyl (S,E)-6-(2,3-bis((benzyloxy)carbonyl)guanidino)-3-((tert-butoxycarbonyl)amino) hexanoate (8c)

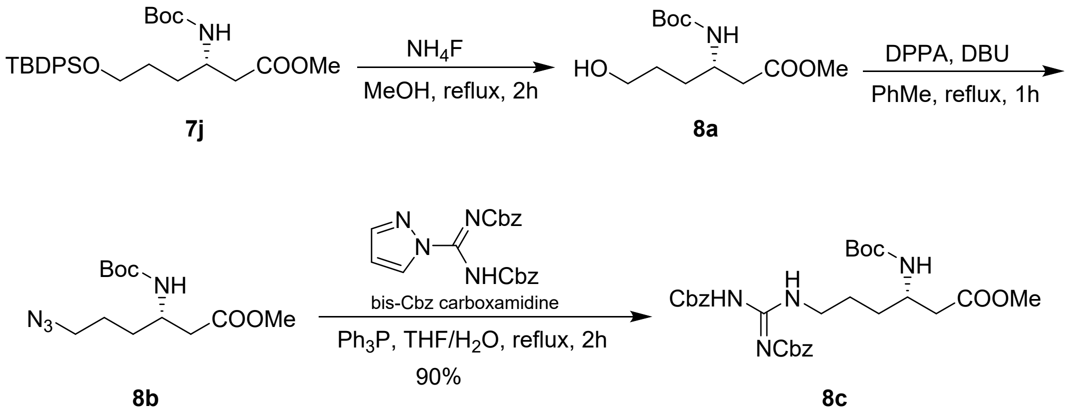

To a solution of **7j** (1.0 *eq.*) in MeOH was added NH_4F (30 *eq.*) at room temperature. Then the resultant mixture was stirred and heated to reflux for 2h. The solution was concentrated in vacuo and the residue was diluted with water. The aqueous phase was extracted with ethyl acetate for 3 times. The combined organic phase was washed by saturated aqueous solution of $NaHCO_3$, brine, dried over sodium sulfate (anhydrous) and concentrated in vacuo to give the corresponding primary alcohol **8a**, which was used immediately for next step directly.

To a solution of the above-obtained primary alcohol **8a** in toluene was added DPPA (2.0 *eq.*) and DBU (2.0 *eq.*). The reaction mixture was heated to reflux for 1h. After concentrated in vacuo, the residue was purified by silica gel column chromatography (EA/PE, 1:4) to afford the compound **8b** (80% over two steps) as an oil.

Compound **8b** :

$[\alpha]_D^{25}$ -3.2 (c 0.65, EtOAc); ^{1}H NMR (600 MHz, $CDCl_3$) δ 4.98 (d, J = 8.7 Hz, 1H), 3.92 (s, 1H), 3.69 (s, 3H), 3.30 (t, J = 6.6 Hz, 2H), 2.54 (qd, J = 15.8, 5.3 Hz, 2H), 1.70 – 1.65 (m, 1H), 1.63 – 1.54 (m, 3H), 1.43 (s, 9H); ^{13}C NMR (151 MHz, $CDCl_3$) δ 171.97, 155.38, 79.47, 51.73, 51.02, 46.98, 39.16, 31.74, 28.34, 25.67; HR-ESIMS m/z: calculated for $C_{12}H_{22}N_4O_4Na^+$ $[M+Na]^+$: 309.1533, found 309.1536.

To a solution of the above-obtained compound **8b** (1.0 *eq.*) in THF/H_2O (20:1) was added bis-Cbz carboxamidine (1.1 *eq.*) and Ph_3P (3.0 *eq.*). The reaction mixture was heated to reflux for 2h. After concentrated in vacuo, the residue was purified by silica gel column chromatography (EA/PE, 1:2) to afford the compound **8c** (90%) as an oil.

Compound **8c** :

$[\alpha]_D^{25}$ -2.1 (c 0.77, EtOAc); ^{1}H NMR (600 MHz, $CDCl_3$) δ 11.76 (s, 1H), 8.34 (s, 1H), 7.56 – 7.22 (m, 10H), 5.18 (t, *J* = 22.1 Hz, 4H), 5.05 (d, *J* = 8.6 Hz, 1H), 3.94 (s, 1H), 3.68 (s, 3H), 3.45 (dd, *J* = 12.5, 6.3 Hz, 2H), 2.53 (dd, *J* = 14.1, 8.3 Hz, 2H), 1.70 – 1.54 (m, 4H), 1.44 (s, 9H); ^{13}C NMR (151 MHz, $CDCl_3$) δ 171.86, 163.61, 155.93, 155.27, 153.76, 136.69, 134.52, 128.69, 128.60, 128.36, 128.30, 127.99, 127.80, 79.27, 77.21, 77.00, 76.79, 68.06, 67.03, 51.60, 47.21, 40.60, 39.11, 31.59, 28.27, 25.82; HR-ESIMS m/z: calculated for $C_{29}H_{38}N_4O_8Na^+$ $[M+Na]^+$: 593.2582, found 593.2585.

Methyl (S)-3-((tert-butoxycarbonyl)amino)-7-((tert-butyldiphenylsilyl)oxy)heptanoate (7k)

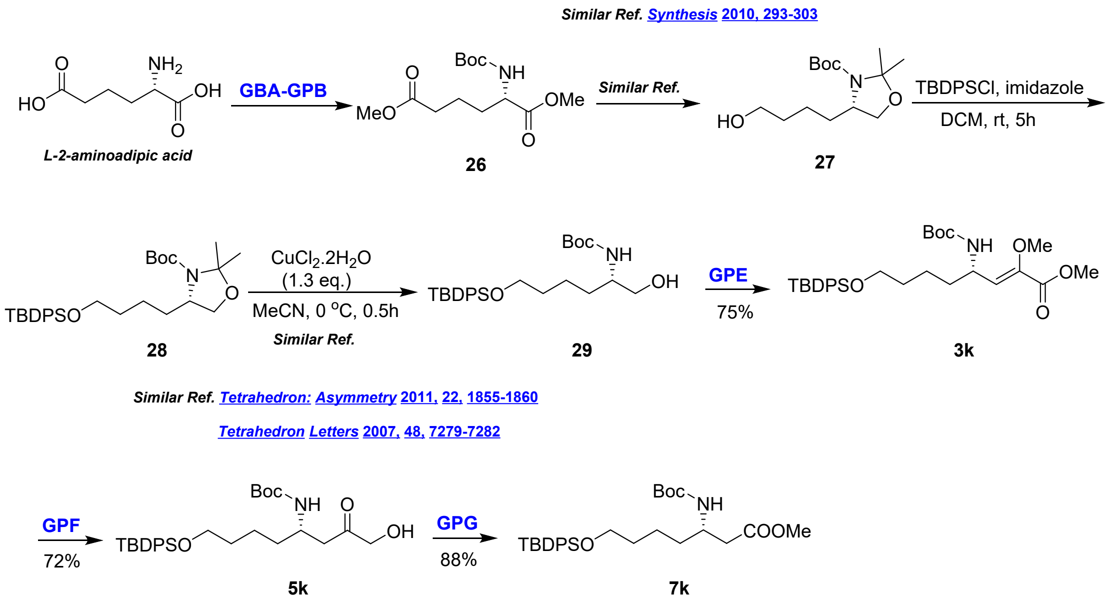

Compound **3k** :

$[\alpha]_D^{25}$ +2.5 (c 0.63, EtOAc); ^{1}H NMR (600 MHz, $CDCl_3$) δ 7.65 (d, *J* = 7.7 Hz, 4H), 7.42 – 7.36 (m, 6H), 6.04 (d, *J* = 8.7 Hz, 1H), 4.65 (s, 1H), 4.52 (s, 1H), 3.78 (s, 3H), 3.72 (s, 3H), 3.64 (t, *J* = 6.3 Hz, 2H), 1.63 – 1.45 (m, 6H), 1.43 (s, 9H), 1.04 (s, 9H); ^{13}C NMR (151 MHz, $CDCl_3$) δ 163.98, 155.02, 145.98, 135.54, 133.97, 129.51, 128.18, 127.58, 79.33, 63.49, 60.14, 51.99, 47.08,

34.89, 32.15, 28.38, 26.83, 21.95, 19.17; HR-ESIMS m/z: calculated for $C_{31}H_{45}NO_6SiNa^+$ $[M+Na]^+$: 578.2908, found 578.2912.

Compound **5k** :

$[\alpha]_D^{25}$ -1.3 (c 0.95, EtOAc); ^{1}H NMR (600 MHz, $CDCl_3$) δ 7.65 (dt, *J* = 8.0, 1.4 Hz, 4H), 7.47 – 7.34 (m, 6H), 4.72 (d, *J* = 8.1 Hz, 1H), 4.22 (dd, *J* = 44.0, 19.0 Hz, 2H), 3.90 (d, *J* = 5.8 Hz, 1H), 3.67 – 3.62 (m, 2H), 3.11 (s, 1H), 2.59 (d, *J* = 4.6 Hz, 2H), 1.55 (ddd, *J* = 8.6, 5.4, 2.5 Hz, 2H), 1.46 (dd, *J* = 13.7, 5.4 Hz, 4H), 1.43 (d, *J* = 13.9 Hz, 9H), 1.04 (s, 9H); ^{13}C NMR (151 MHz, $CDCl_3$) δ 208.60, 155.40, 135.54, 133.92, 129.55, 127.60, 79.56, 68.67, 63.48, 47.65, 43.51, 34.45, 32.05, 28.31, 26.85, 22.49, 19.18; HR-ESIMS m/z: calculated for $C_{29}H_{43}NO_5SiNa^+$ $[M+Na]^+$: 536.2803, found 536.2806.

Compound **7k** :

$[\alpha]_D^{25}$ -4.7 (c 0.57, EtOAc); ^{1}H NMR (600 MHz, $CDCl_3$) δ 7.65 (dt, *J* = 8.1, 1.4 Hz, 4H), 7.39 (dt, *J* = 14.3, 7.0 Hz, 6H), 4.88 (d, *J* = 8.8 Hz, 1H), 3.88 (s, 1H), 3.67 (s, 3H), 3.64 (t, *J* = 6.2 Hz, 2H), 2.49 (qd, *J* = 15.5, 5.2 Hz, 2H), 1.59 – 1.52 (m, 2H), 1.50 – 1.43 (m, 4H), 1.45 – 1.39 (m, 9H), 1.04 (s, 9H); ^{13}C NMR (151 MHz, $CDCl_3$) δ 172.16, 155.33, 135.55, 133.99, 129.51, 127.59, 79.17, 63.58, 51.60, 47.56, 39.10, 34.30, 32.19, 28.36, 26.85, 22.47, 19.18; HR-ESIMS m/z: calculated for $C_{29}H_{43}NO_5SiNa^+$ $[M+Na]^+$: 536.2803, found 536.2806.

Tert-butyl (S)-2-(2-methoxy-2-oxoethyl)pyrrolidine-1-carboxylate (9a)

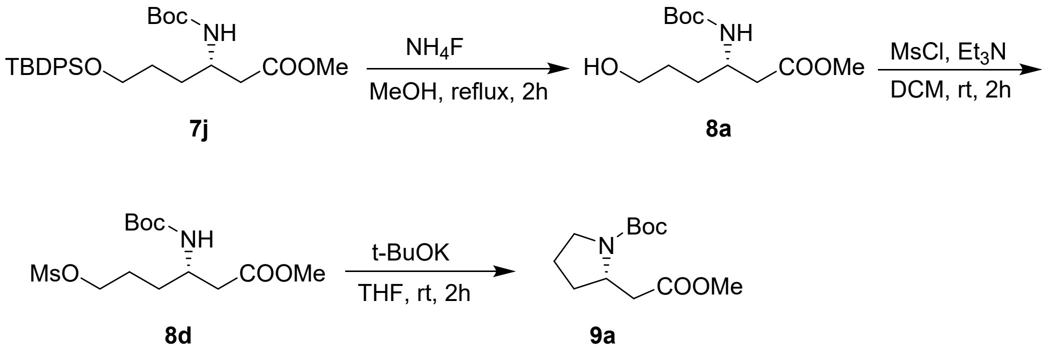

To a solution of **7j** (1.0 *eq.*) in MeOH was added NH_4F (30 *eq.*) at room temperature. Then the resultant mixture was stirred and heated to reflux for 2h. The solution was concentrated in vacuo and the residue was diluted with water. The aqueous phase was extracted with ethyl acetate for 3 times. The combined organic phase was washed by saturated aqueous solution of $NaHCO_3$,

brine, dried over sodium sulfate (anhydrous) and concentrated in vacuo to give the corresponding primary alcohol **8a**, which was used immediately for next step directly.

To a solution of the above-obtained primary alcohol **8a** in dry DCM was added Et_3N (3.0 *eq.*) and MsCl (2.0 *eq.*) at 0 °C. After 15 min, the resultant mixture was allowed to warm to room temperature and stirred for 2h at N_2 atmosphere. The reaction was then quenched with saturated aqueous $NaHCO_3$ solution and extracted with DCM for 3 times. The combined organic layer was washed successively with saturated aqueous $KHSO_4$ solution, saturated aqueous solution of $NaHCO_3$, brine, and dried over anhydrous Na_2SO_4, filtered, concentrated in vacuo to give the compound **8d** as an oil, which was pure enough and could be used for next step directly.

To a solution of the above-obtained compound **8d** in dry THF was added t-BuOK (2.0 *eq.*) at 0 °C. After 20 min, the resultant mixture was allowed to warm to room temperature and stirred for 2h at N_2 atmosphere. The reaction was then quenched with saturated aqueous NH_4Cl solution. Volatiles of the reaction mixture were removed in vacuo. The solution was then diluted with water. The aqueous phase was extracted with ethyl acetate for 3 times. The combined organic phase was washed by brine, dried over sodium sulfate (anhydrous) and concentrated in *vacuo*. The residue was purified by silica gel column chromatography (EA/PE, 1:4) to afford the compound **9a** (80% over 3 steps) as an oil.

$[\alpha]_D^{25}$ -18.5 (c 0.13, EtOAc); ^1H NMR (600 MHz, *CD_3CN*) (*exists as rotamers*) δ 4.03 (dd, *J* = 10.5, 6.4 Hz, 1H), 3.61 (s, 3H), 3.27 (t, *J* = 7.3 Hz, 2H), 2.75 (s, 1H), 2.38 – 2.28 (m, 1H), 2.01 (s, 1H), 1.86 – 1.74 (m, 2H), 1.69 (s, 1H), 1.42 (s, 9H); ^{13}C NMR (151 MHz, *CD_3CN*) (*exists as rotamers*) δ 172.85, 155.17, 154.94, 118.36, 79.84, 79.66, 55.11, 52.04, 47.36, 46.99, 39.72, 39.07, 31.90, 31.20, 28.64, 24.14, 23.36, 1.73, 1.60, 1.46, 1.32, 1.18, 1.04, 0.91; HR-ESIMS m/z: calculated for $C_{12}H_{21}NO_4Na^+$ $[M+Na]^+$: 266.1363, found 266.1365.

Methyl (S)-7-azido-3-((tert-butoxycarbonyl)amino)heptanoate (8g)

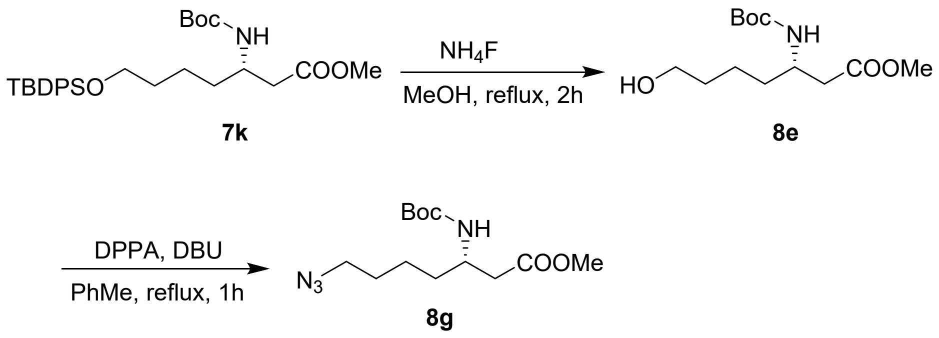

To a solution of **7k** (1.0 *eq.*) in MeOH was added NH_4F (30 *eq.*) at room temperature. Then the resultant mixture was stirred and heated to reflux for 2h. The solution was concentrated in vacuo and the residue was diluted with water. The aqueous phase was extracted with ethyl acetate for 3 times. The combined organic phase was washed by saturated aqueous solution of $NaHCO_3$, brine, dried over sodium sulfate (anhydrous) and concentrated in vacuo to give the corresponding primary alcohol **8e**, which was used immediately for next step directly.

To a solution of the above-obtained primary alcohol **8e** in toluene was added DPPA (2.0 *eq.*) and DBU (2.0 *eq.*). The reaction mixture was heated to reflux for 1h. After concentrated in vacuo, the residue was purified by silica gel column chromatography (EA/PE, 1:4) to afford the compound **8g** (82% over two steps) as an oil.

$[\alpha]_D^{25}$ -7.0 (c 0.87, EtOAc); ^{1}H NMR (600 MHz, $CDCl_3$) δ 4.94 (d, *J* = 8.7 Hz, 1H), 3.90 (d, *J* = 5.1 Hz, 1H), 3.67 (s, 3H), 3.33 – 3.18 (m, 2H), 2.51 (qd, *J* = 15.6, 5.3 Hz, 2H), 1.66 – 1.43 (m, 6H), 1.42 (s, 9H); ^{13}C NMR (151 MHz, $CDCl_3$) δ 172.03, 155.33, 79.30, 51.64, 51.22, 47.24, 39.03, 34.04, 28.48, 28.31, 23.30; HR-ESIMS m/z: calculated for $C_{13}H_{24}N_4O_4Na^+$ $[M+Na]^+$: 323.1690, found 323.1694.

Tert-butyl (S)-2-(2-methoxy-2-oxoethyl)piperidine-1-carboxylate (9b)

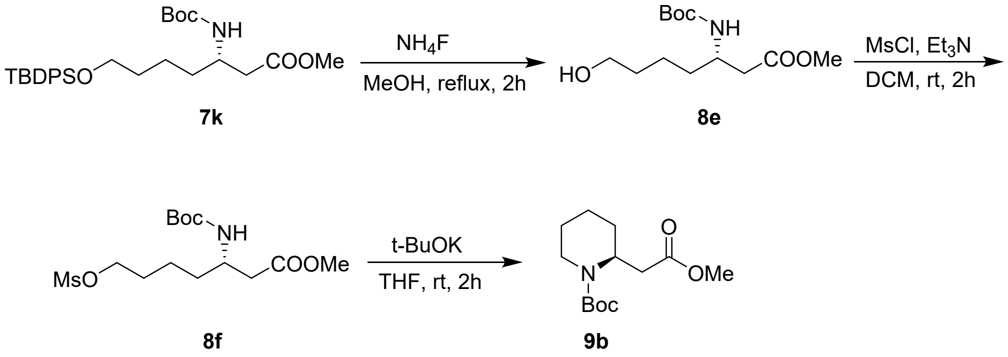

To a solution of **7k** (1.0 *eq.*) in MeOH was added NH_4F (30 *eq.*) at room temperature. Then the resultant mixture was stirred and heated to reflux for 2h. The solution was concentrated in vacuo and the residue was diluted with water. The aqueous phase was extracted with ethyl acetate for 3 times. The combined organic phase was washed by saturated aqueous solution of $NaHCO_3$, brine, dried over sodium sulfate (anhydrous) and concentrated in vacuo to give the corresponding primary alcohol **8e**, which was used immediately for next step directly.

To a solution of the above-obtained primary alcohol **8e** in dry DCM was added Et_3N (3.0 *eq.*) and MsCl (2.0 *eq.*) at 0 °C. After 15 min, the resultant mixture was allowed to warm to room temperature and stirred for 2h at N_2 atmosphere. The reaction was then quenched with saturated aqueous $NaHCO_3$ solution and extracted with DCM for 3 times. The combined organic layer was washed successively with saturated aqueous $KHSO_4$ solution, saturated aqueous solution of $NaHCO_3$, brine, and dried over anhydrous Na_2SO_4, filtered, concentrated in vacuo to give the compound **8f** as an oil, which was pure enough and could be used for next step directly.

To a solution of the above-obtained compound **8f** in dry THF was added t-BuOK (2.0 *eq.*) at 0 °C. After 20 min, the resultant mixture was allowed to warm to room temperature and stirred for 2h at N_2 atmosphere. The reaction was then quenched with saturated aqueous NH_4Cl solution. Volatiles of the reaction mixture were removed in vacuo. The solution was then diluted with water. The aqueous phase was extracted with ethyl acetate for 3 times. The combined organic phase was washed by brine, dried over sodium sulfate (anhydrous) and concentrated in *vacuo*. The residue was purified by silica gel column chromatography (EA/PE, 1:4) to afford the compound **9b** (75% over 3 steps) as an oil.

$[\alpha]_D^{25}$ +1.9 (c 0.10, MeOH); ^{1}H NMR (600 MHz, $CDCl_3$) δ 4.66 - 4.68 (m, 1H), 3.97 – 3.99 (m, 1H),

3.64 (s, 3H), 2.77 – 2.74 (m, 1H), 2.54 (ddd, *J* = 45.2, 14.1, 7.6 Hz, 2H), 1.66 – 1.56 (m, 4H), 1.53 – 1.46 (m, 2H), 1.43 (s, 9H); ^{13}C NMR (151 MHz, $CDCl_3$) δ 171.85, 154.68, 79.50, 51.63, 47.82, 39.06, 35.07, 28.35, 28.28, 25.24, 18.82; HR-ESIMS m/z: calculated for $C_{13}H_{23}NO_4Na^+$ $[M+Na]^+$: 280.1519, found 280.1523.

NMR Spectra of New Compounds and Selected Known Compounds

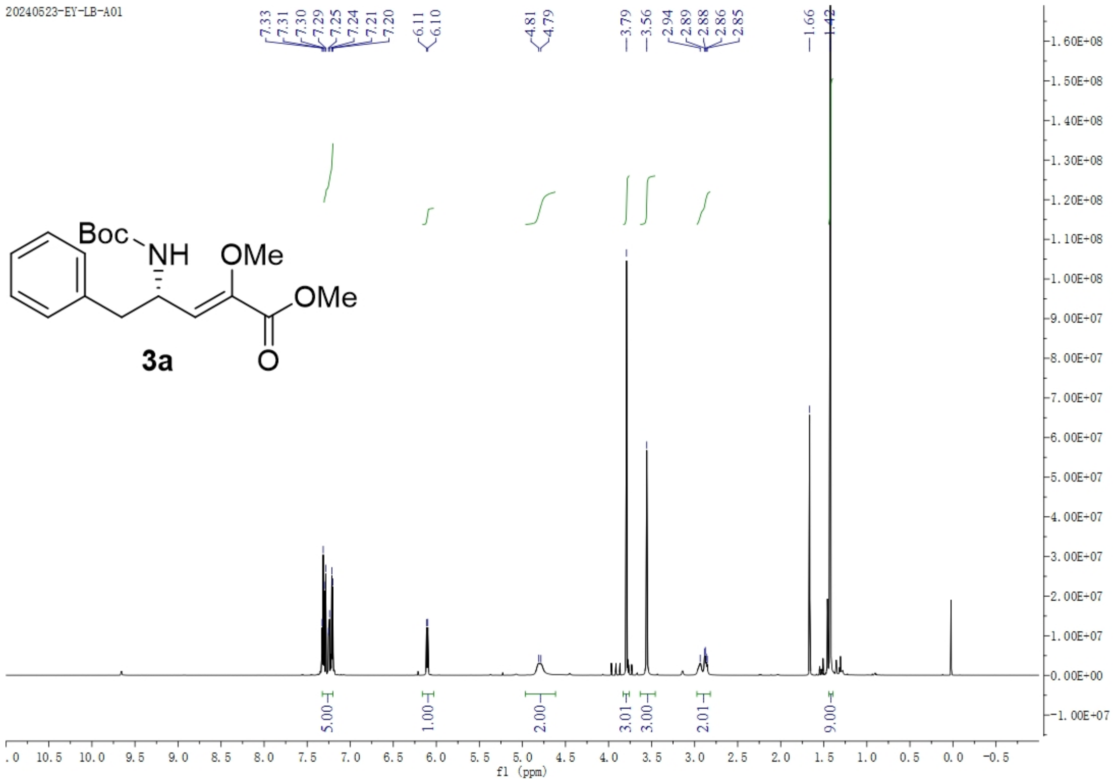

Figure S1. ^{1}H NMR of **3a** ($CDCl_3$, 600 MHz)

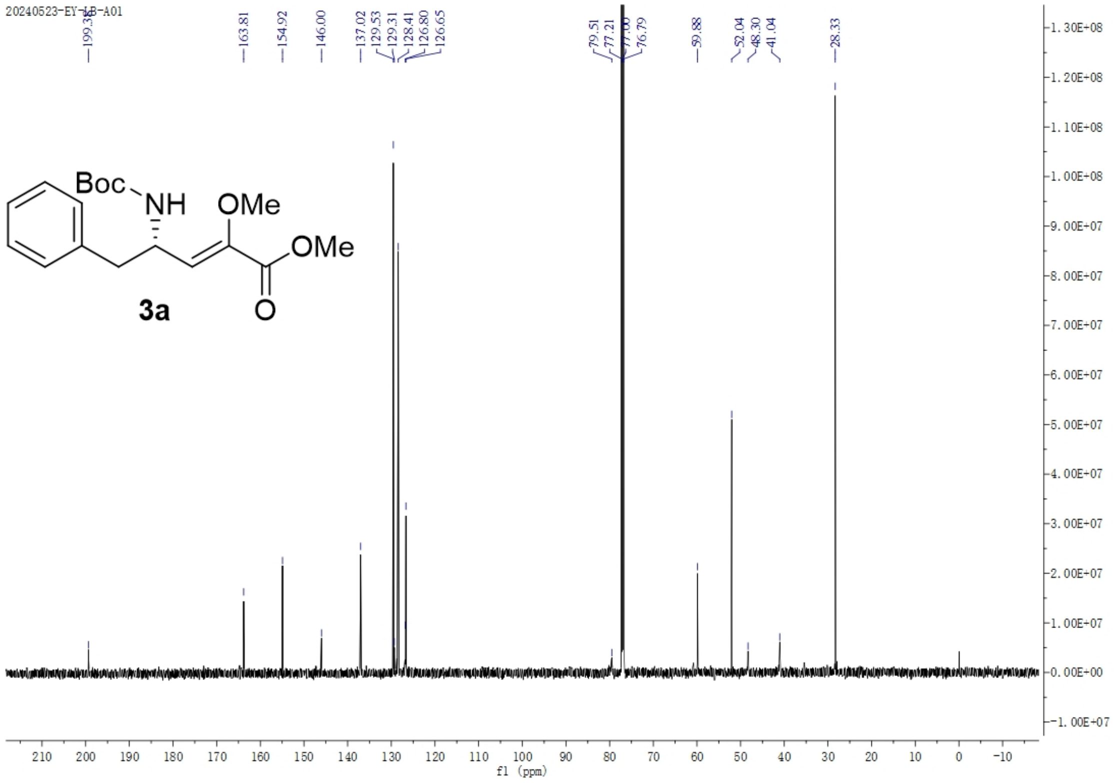

Figure S2. ^{13}C NMR of **3a** ($CDCl_3$, 150 MHz)

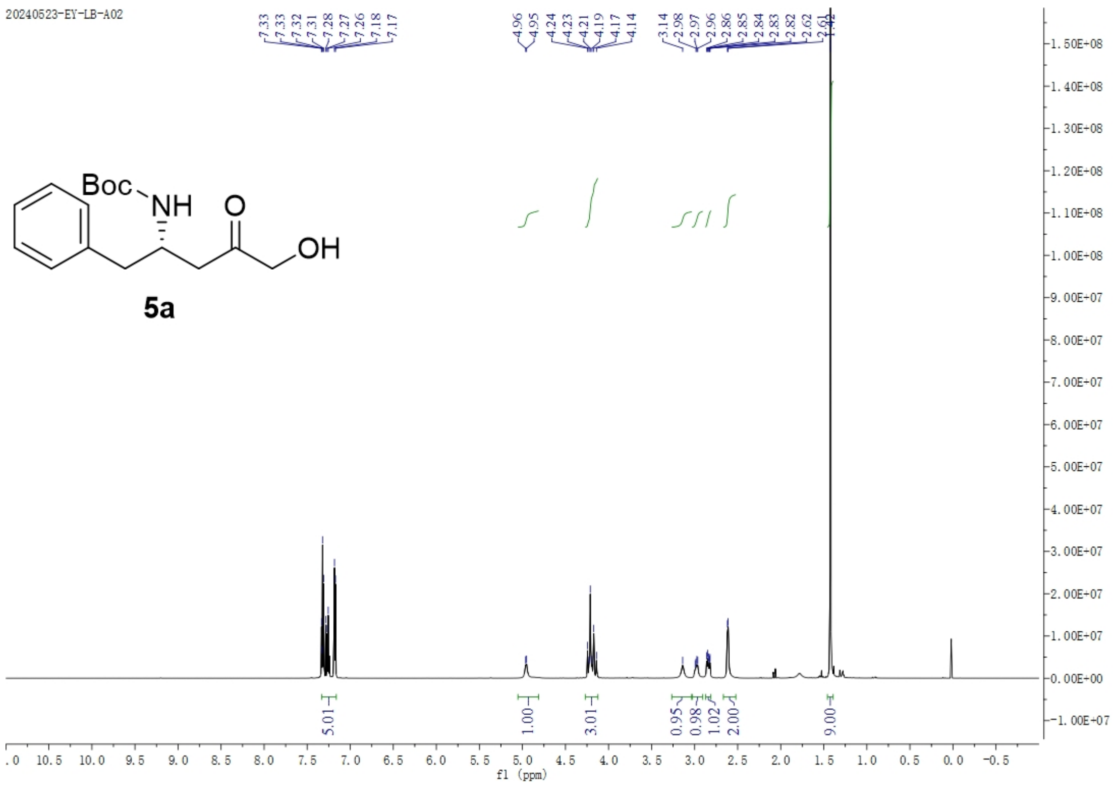

Figure S3. ^{1}H NMR of **5a** ($CDCl_3$, 600 MHz)

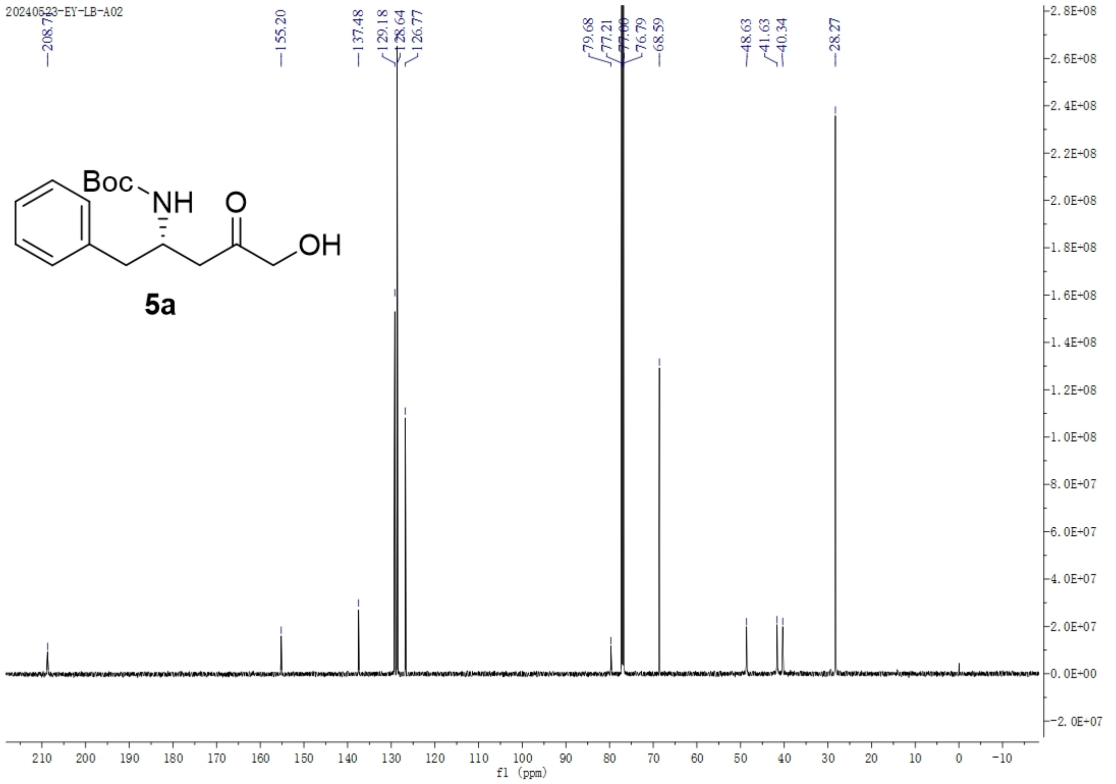

Figure S4. ^{13}C NMR of **5a** ($CDCl_3$, 150 MHz)

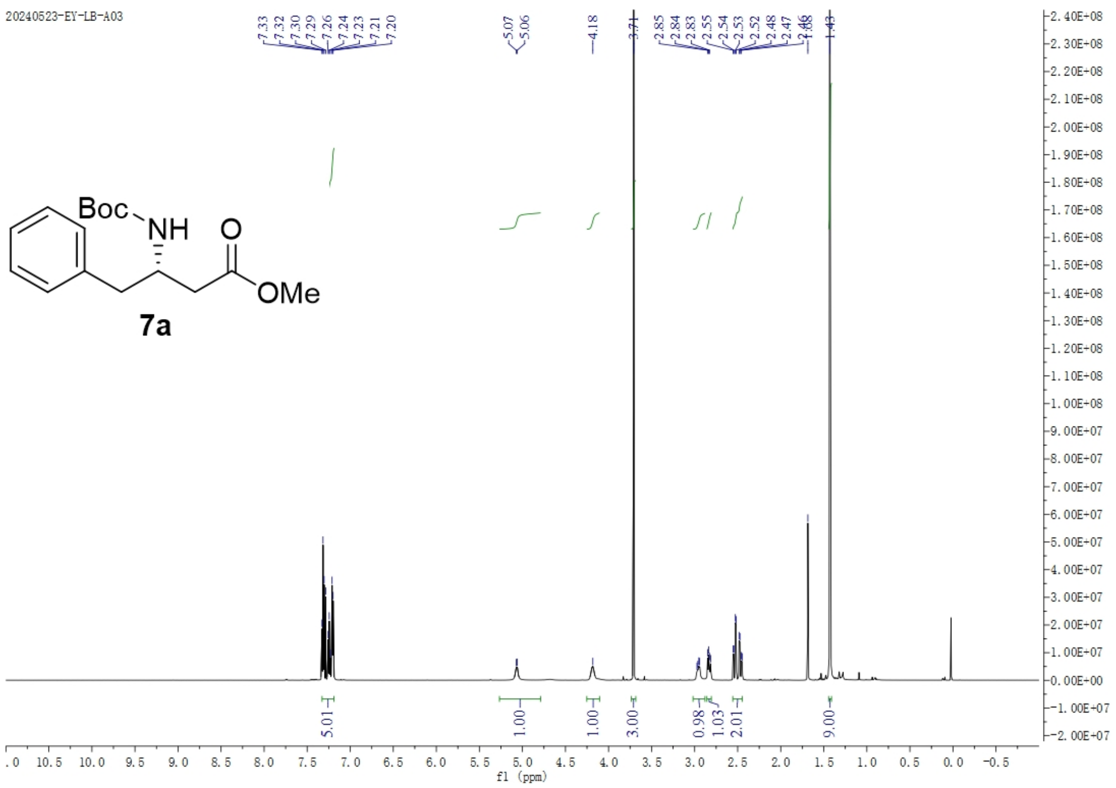

Figure S5. ^{1}H NMR of **7a** ($CDCl_3$, 600 MHz)

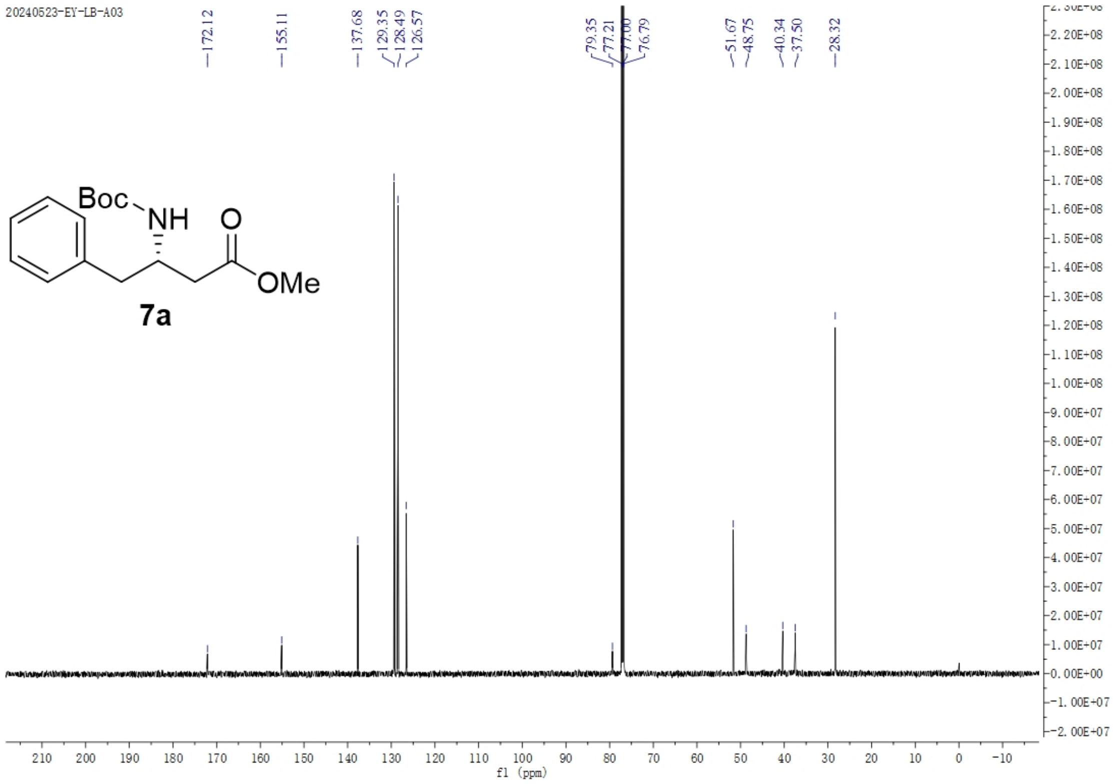

Figure S6. ^{13}C NMR of **7a** ($CDCl_3$, 150 MHz)

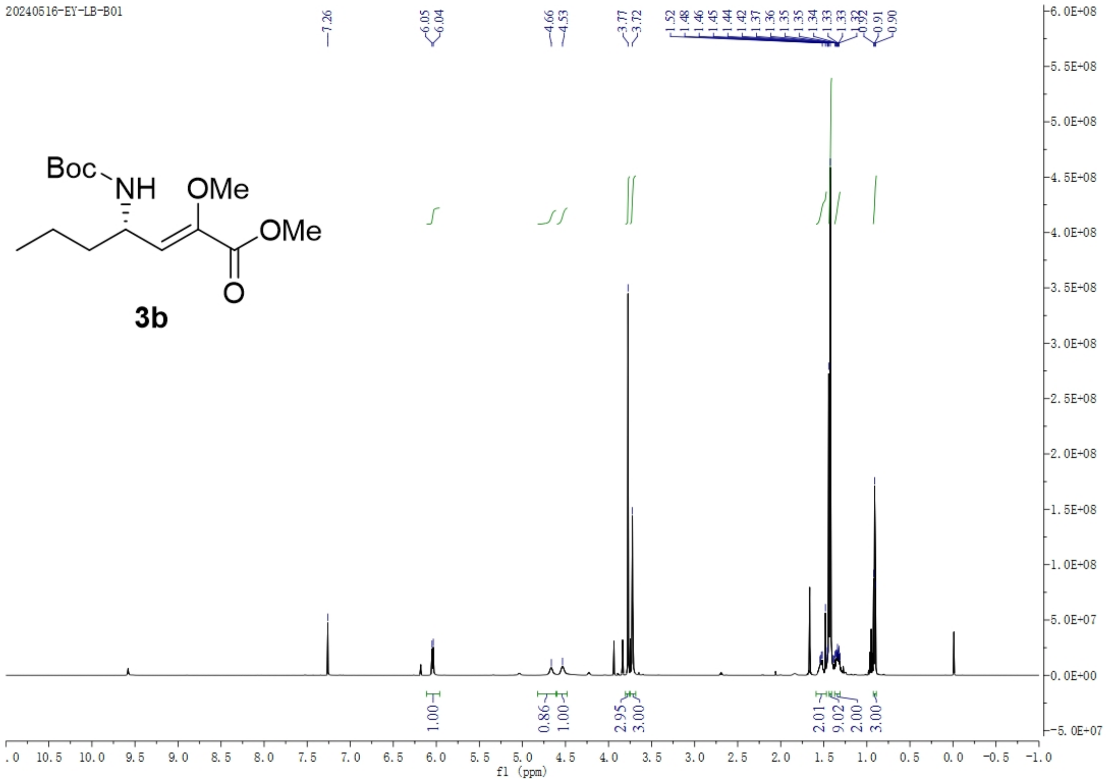

Figure S7. ^{1}H NMR of **3b** ($CDCl_3$, 600 MHz)

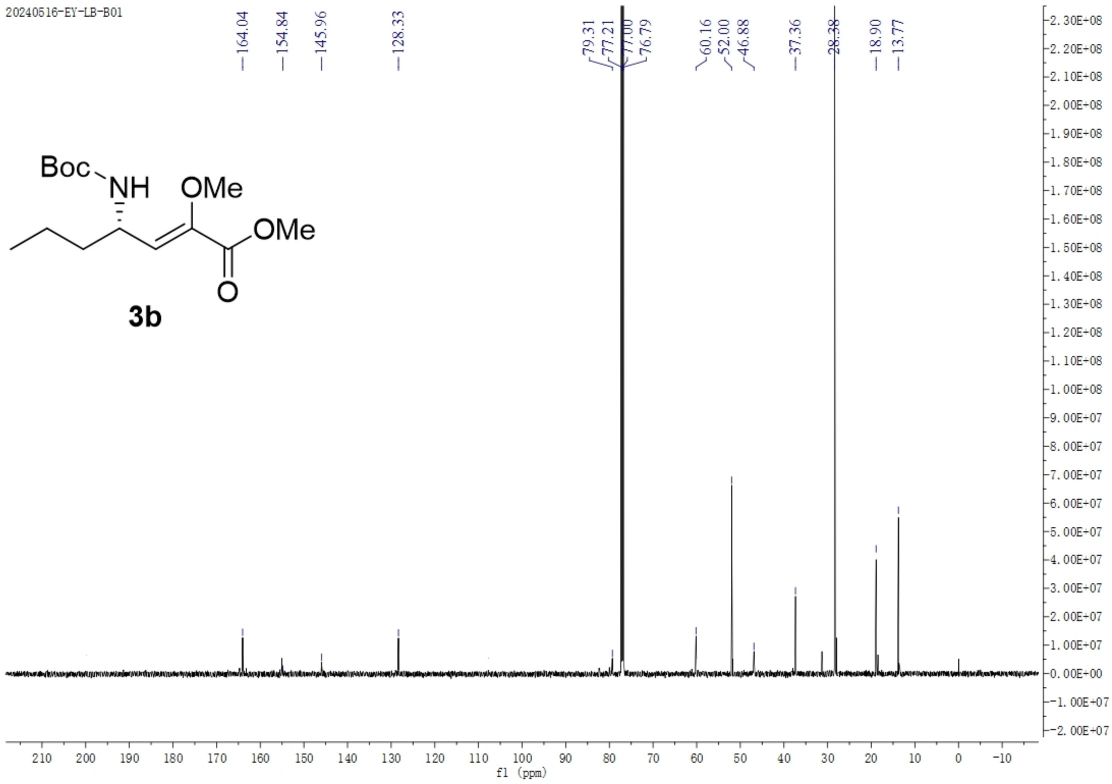

Figure S8. ^{13}C NMR of **3b** ($CDCl_3$, 150 MHz)

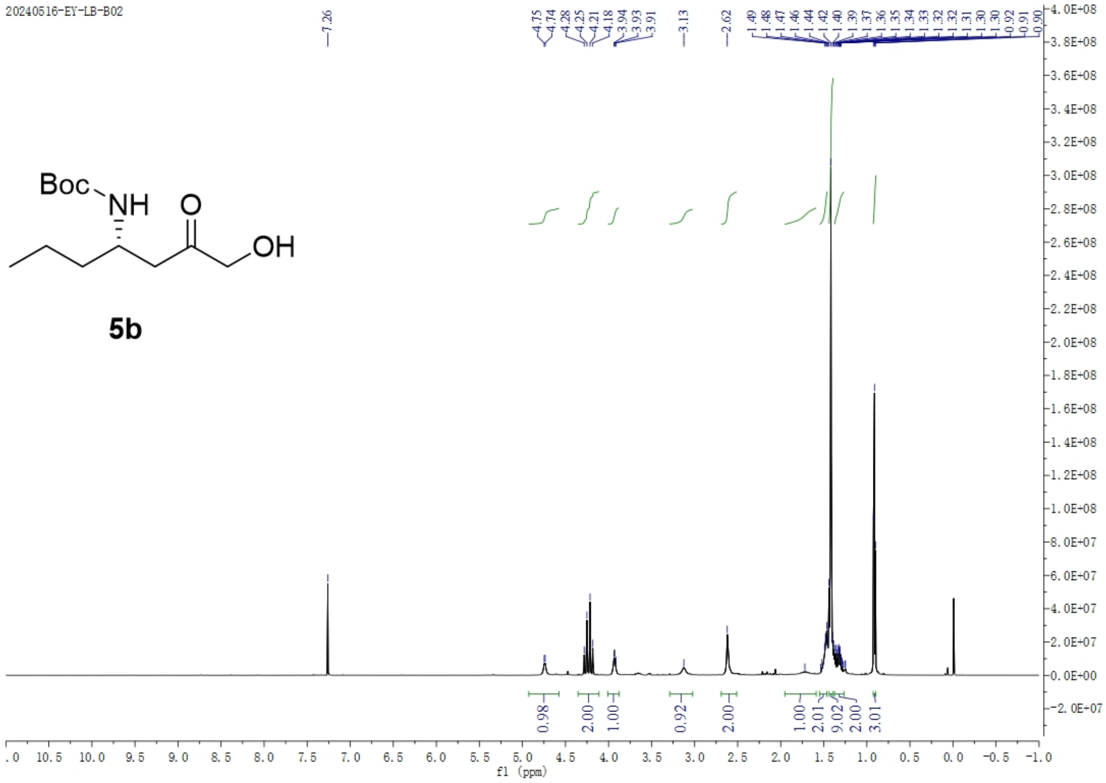

Figure S9. ^{1}H NMR of **5b** ($CDCl_3$, 600 MHz)

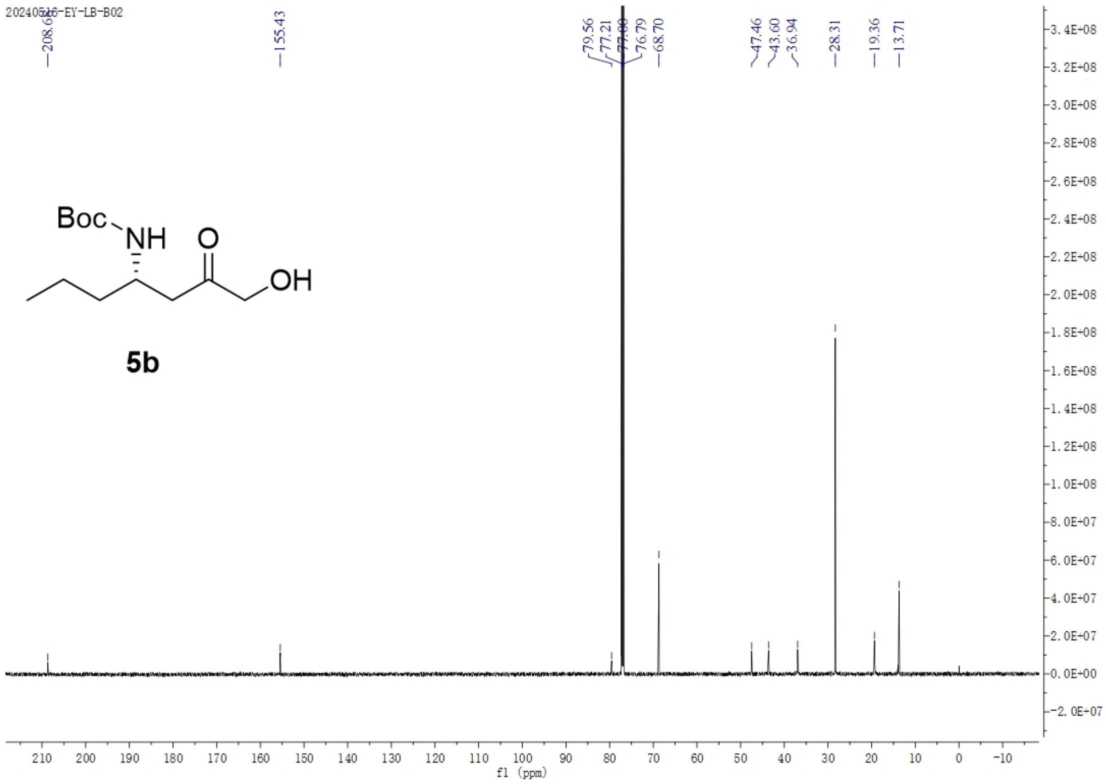

Figure S10. ^{13}C NMR of **5b** ($CDCl_3$, 150 MHz)

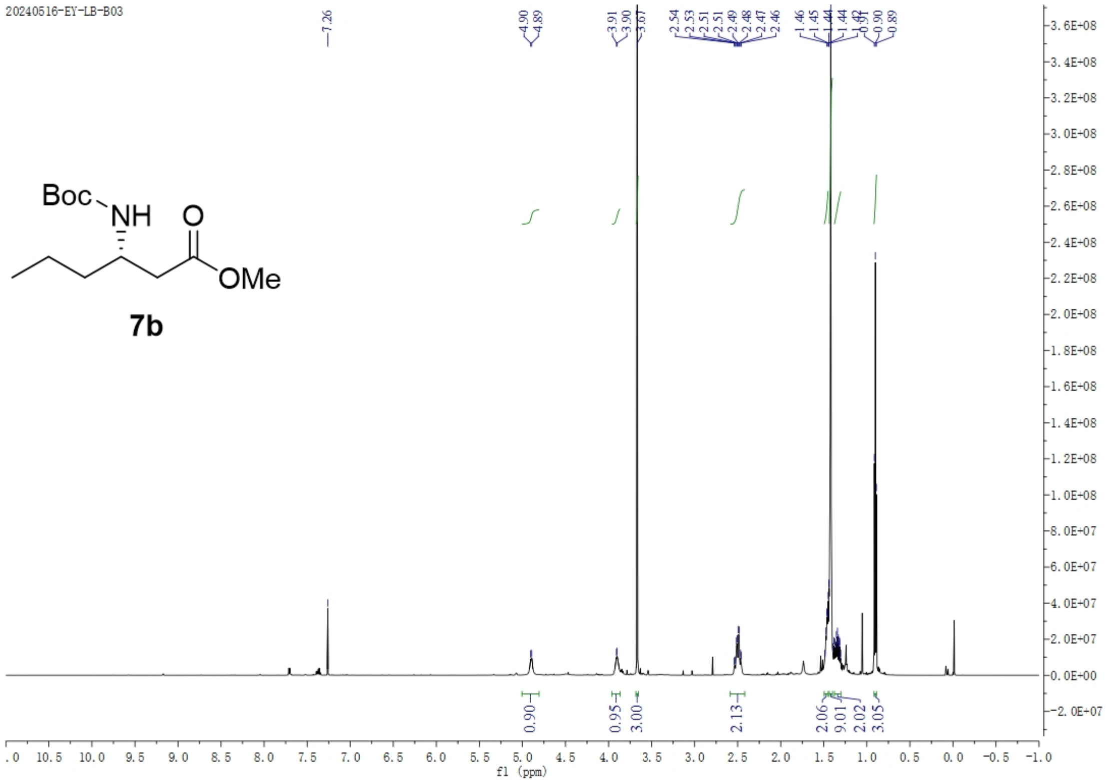

Figure S11. ^{1}H NMR of **7b** ($CDCl_3$, 600 MHz)

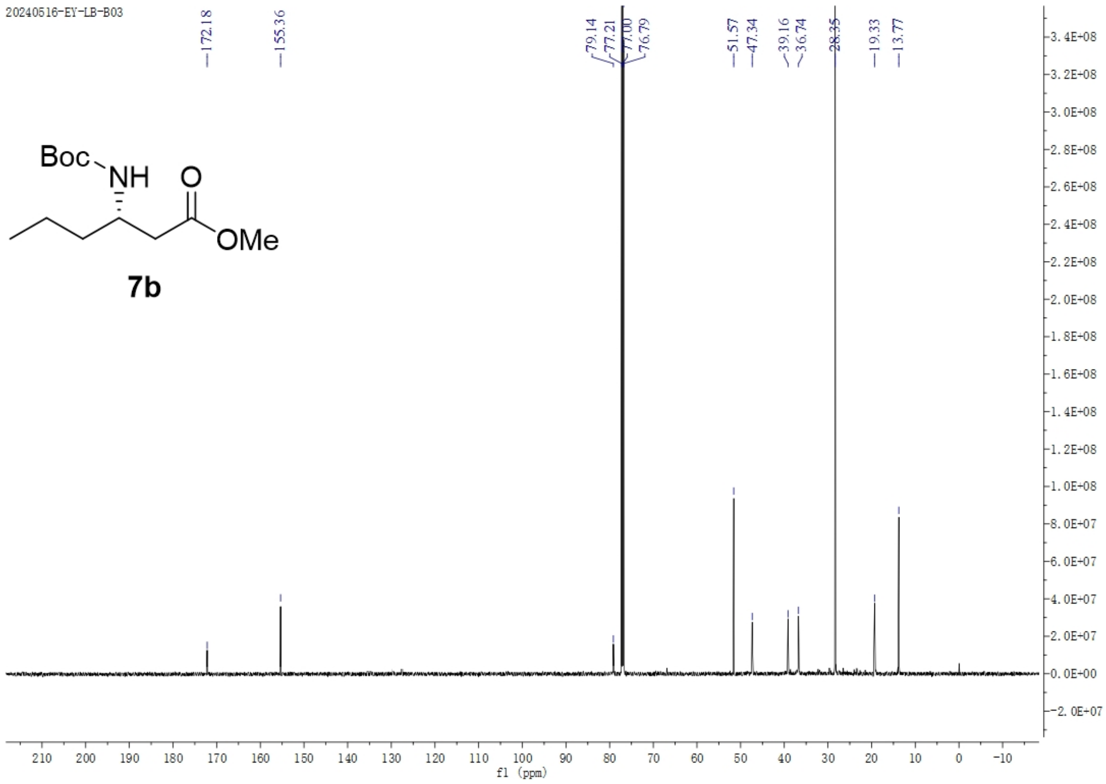

Figure S12. ^{13}C NMR of **7b** ($CDCl_3$, 150 MHz)

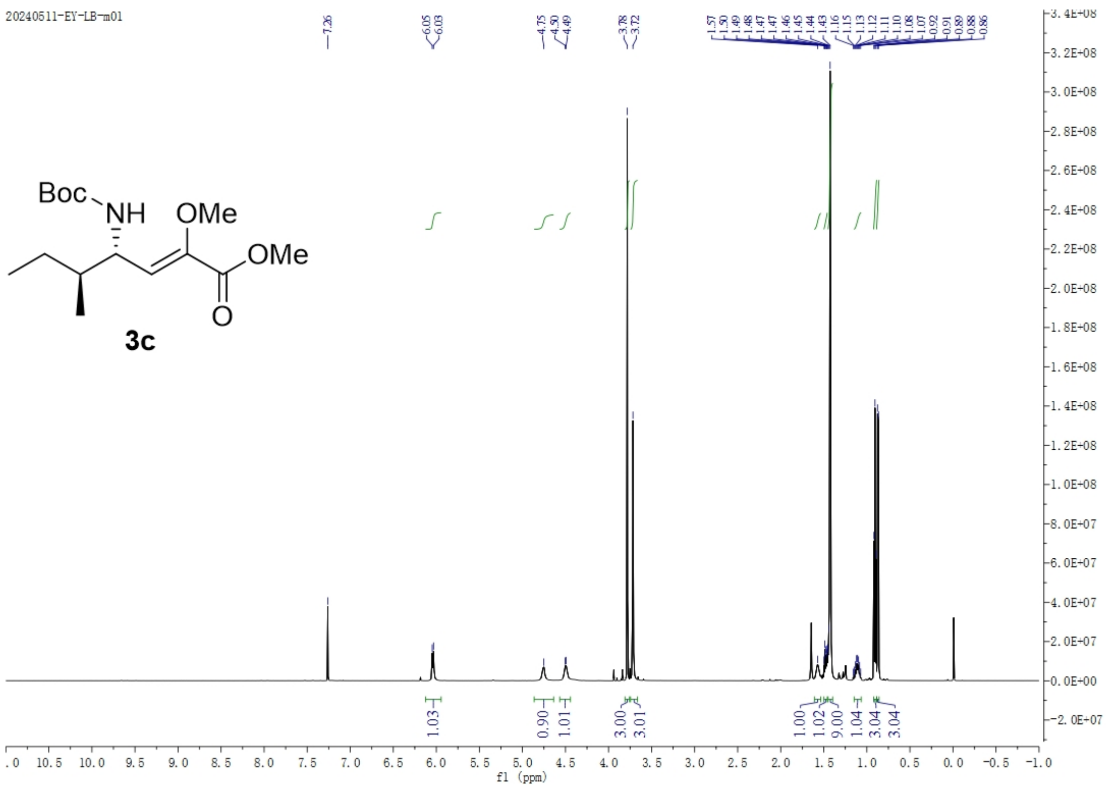

Figure S13. ^{1}H NMR of **3c** ($CDCl_3$, 600 MHz)

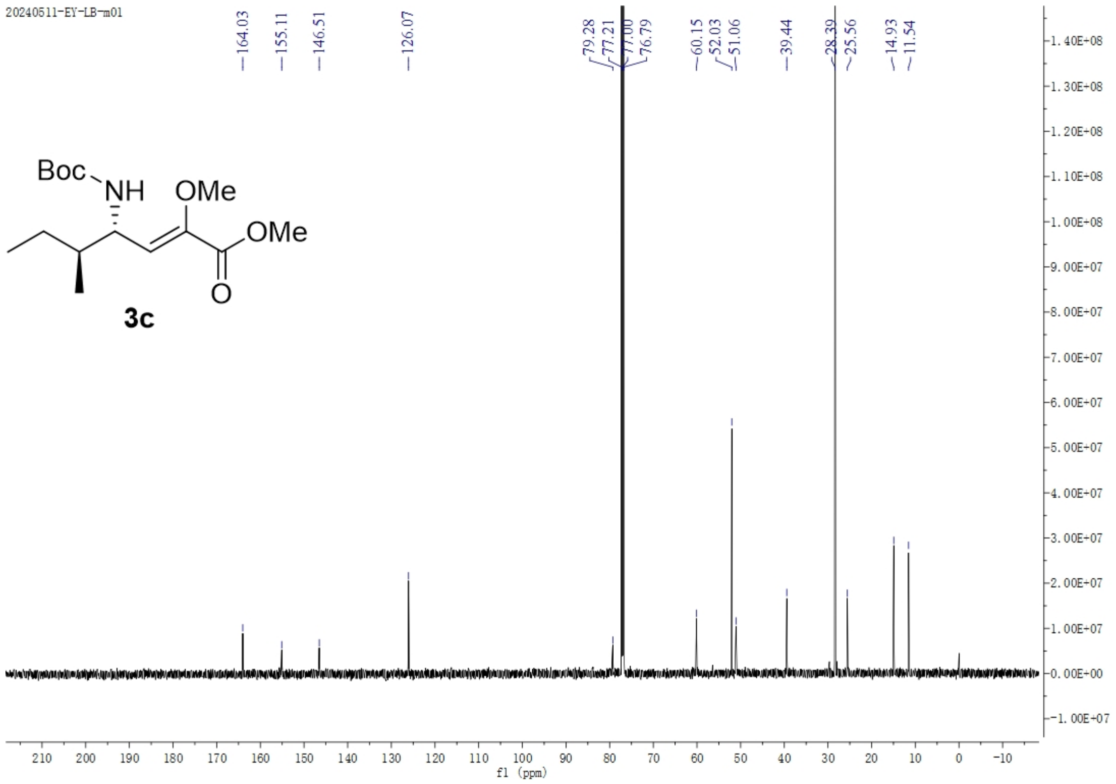

Figure S14. ^{13}C NMR of **3c** ($CDCl_3$, 150 MHz)

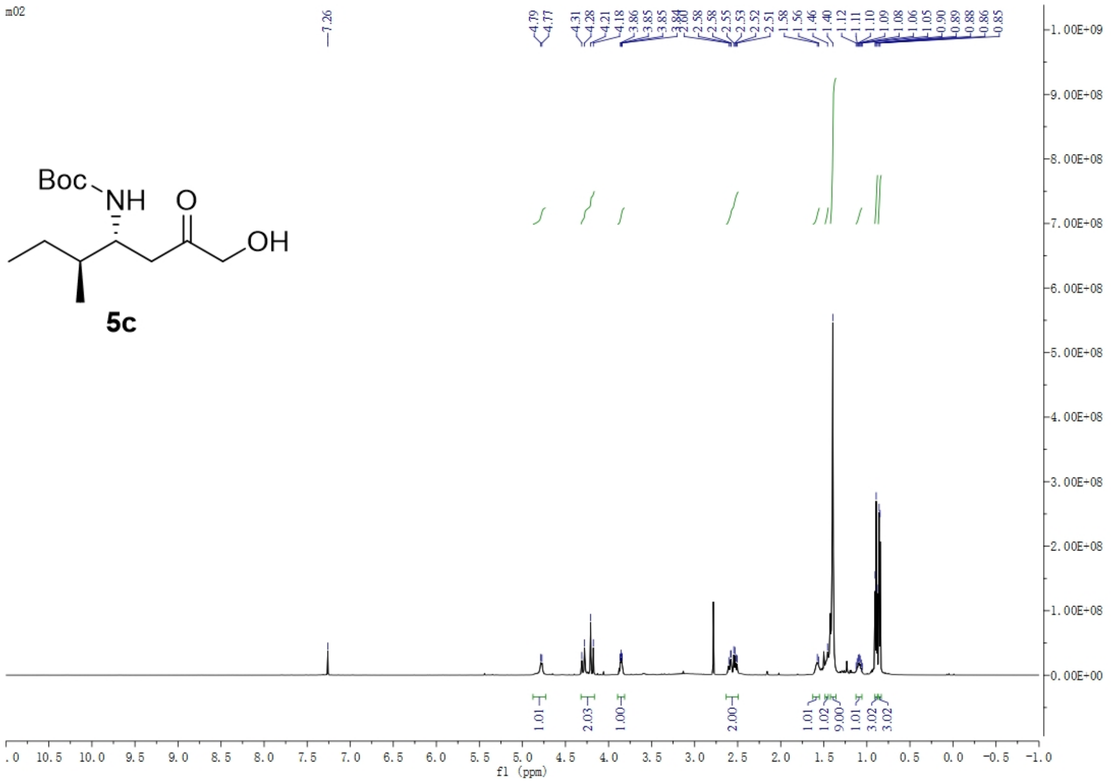

Figure S15. ^{1}H NMR of **5c** ($CDCl_3$, 600 MHz)

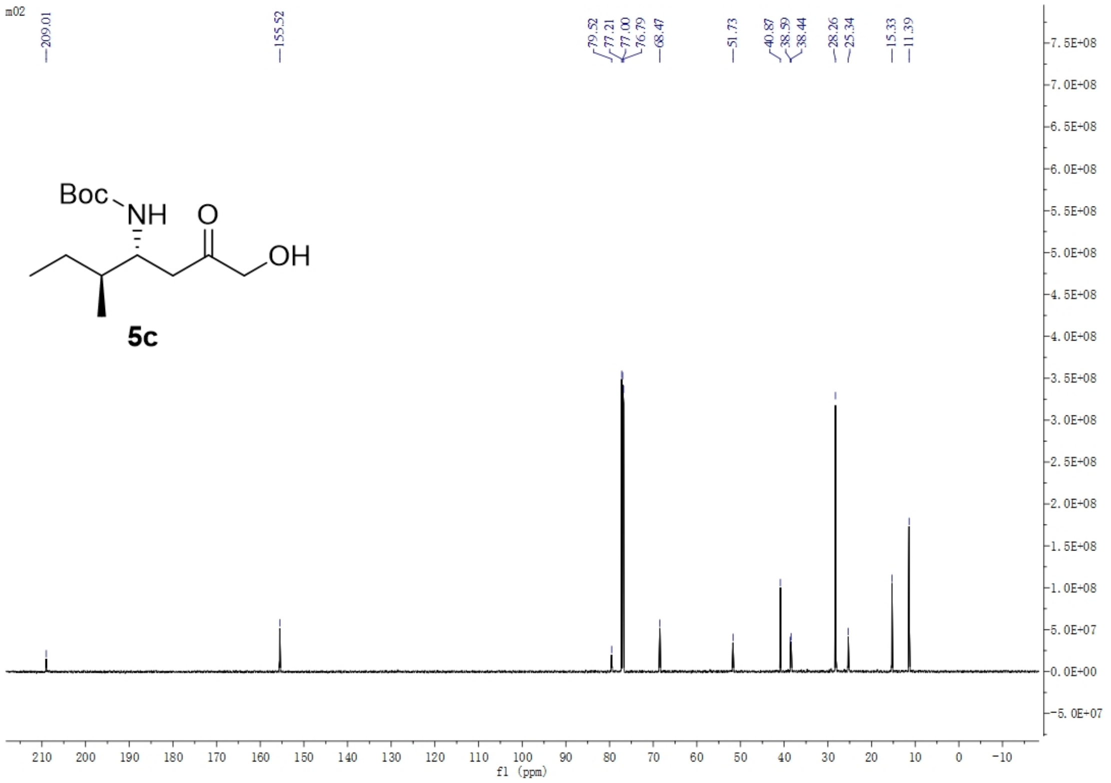

Figure S16. ^{13}C NMR of **5c** ($CDCl_3$, 150 MHz)

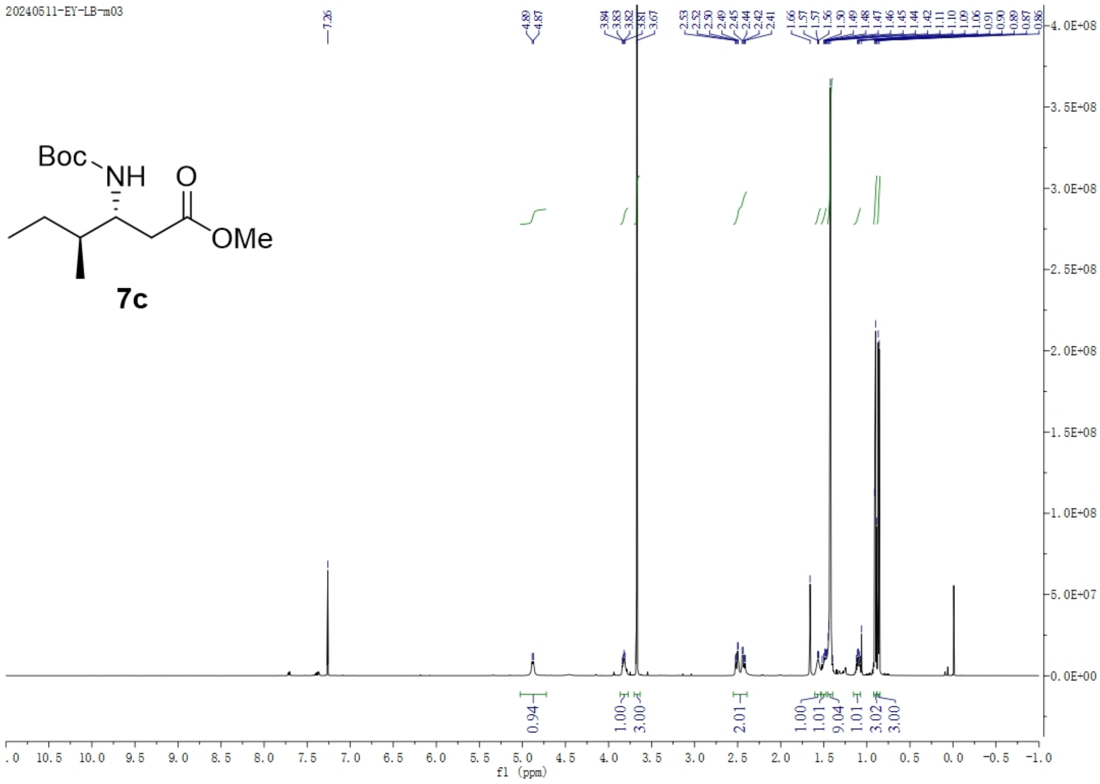

Figure S17. ^{1}H NMR of **7c** ($CDCl_3$, 600 MHz)

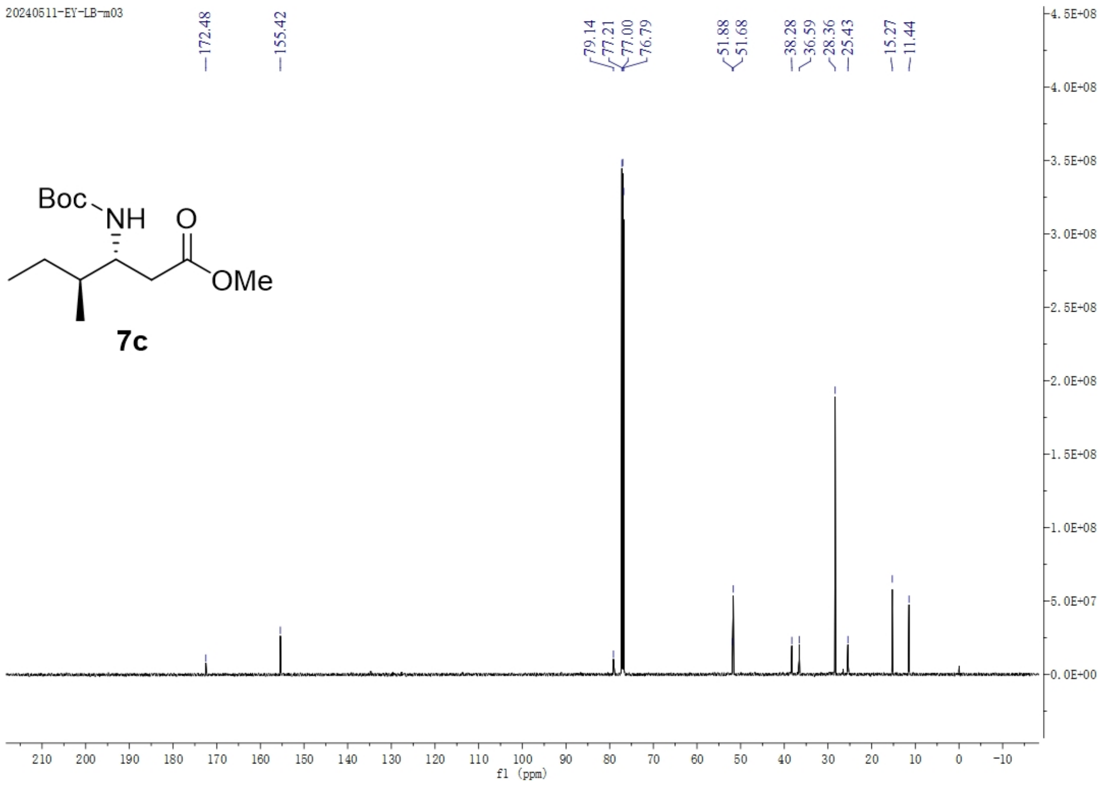

Figure S18. ^{13}C NMR of **7c** ($CDCl_3$, 150 MHz)

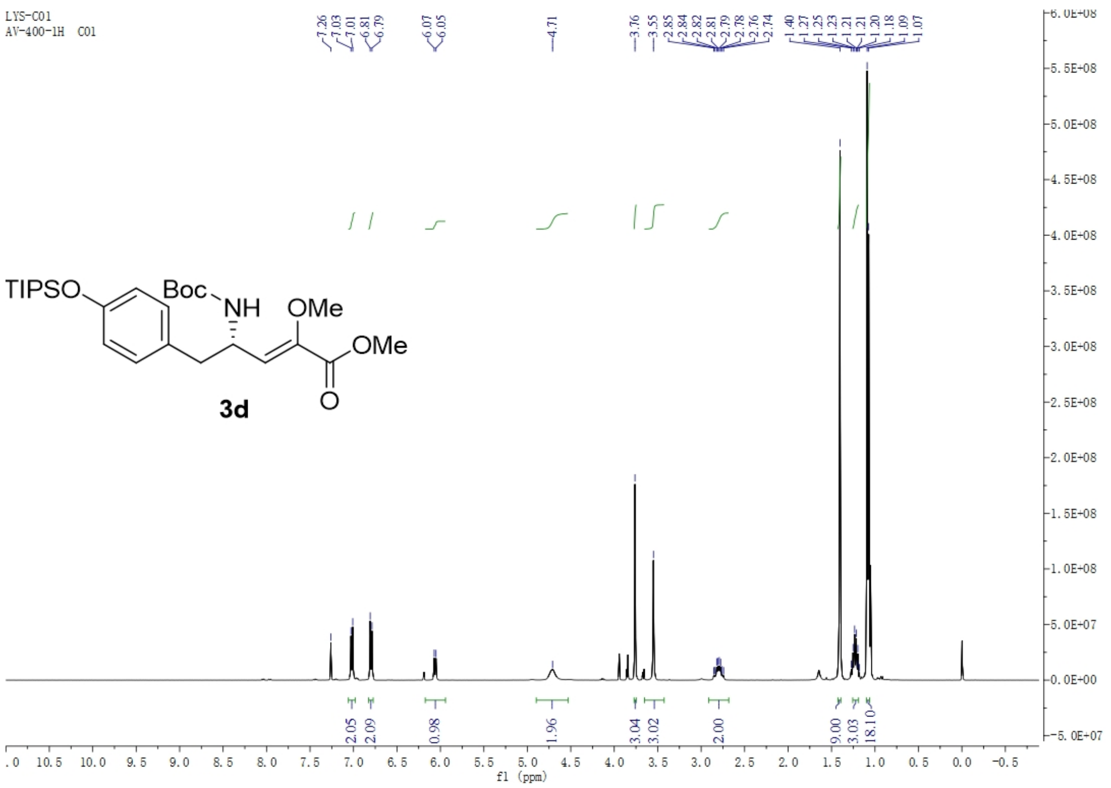

Figure S19. ^{1}H NMR of **3d** ($CDCl_3$, 400 MHz)

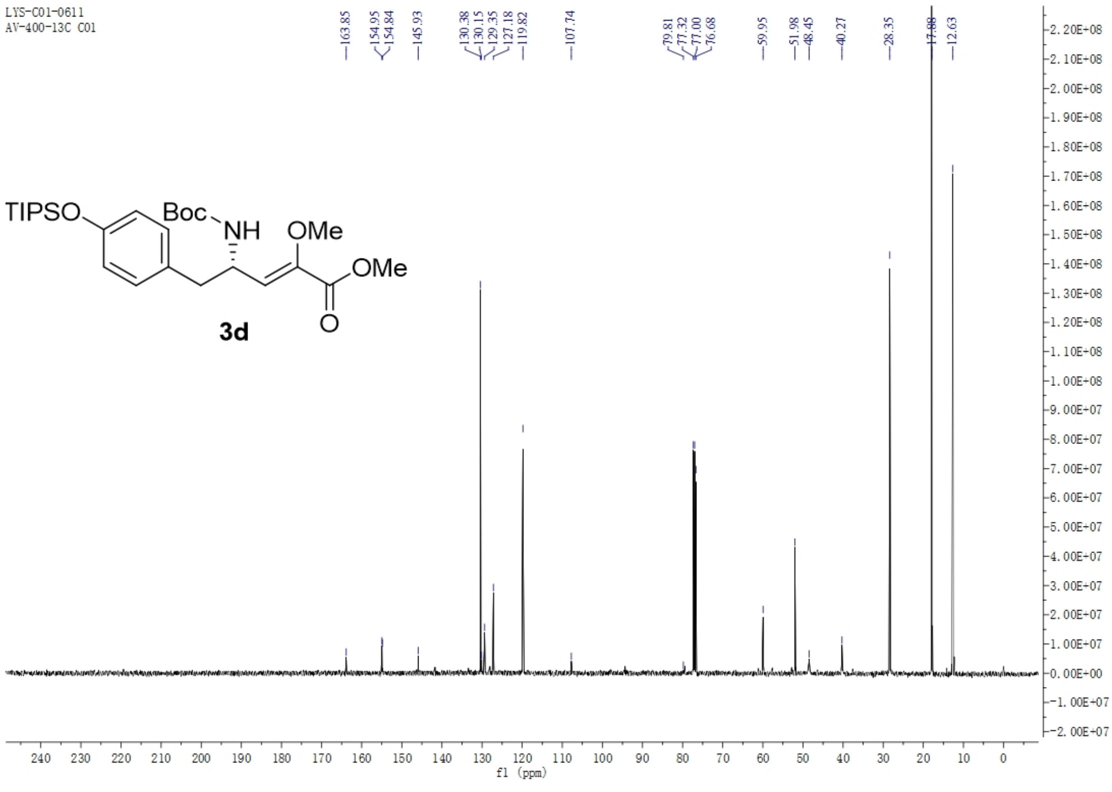

Figure S20. ^{13}C NMR of **3d** ($CDCl_3$, 100 MHz)

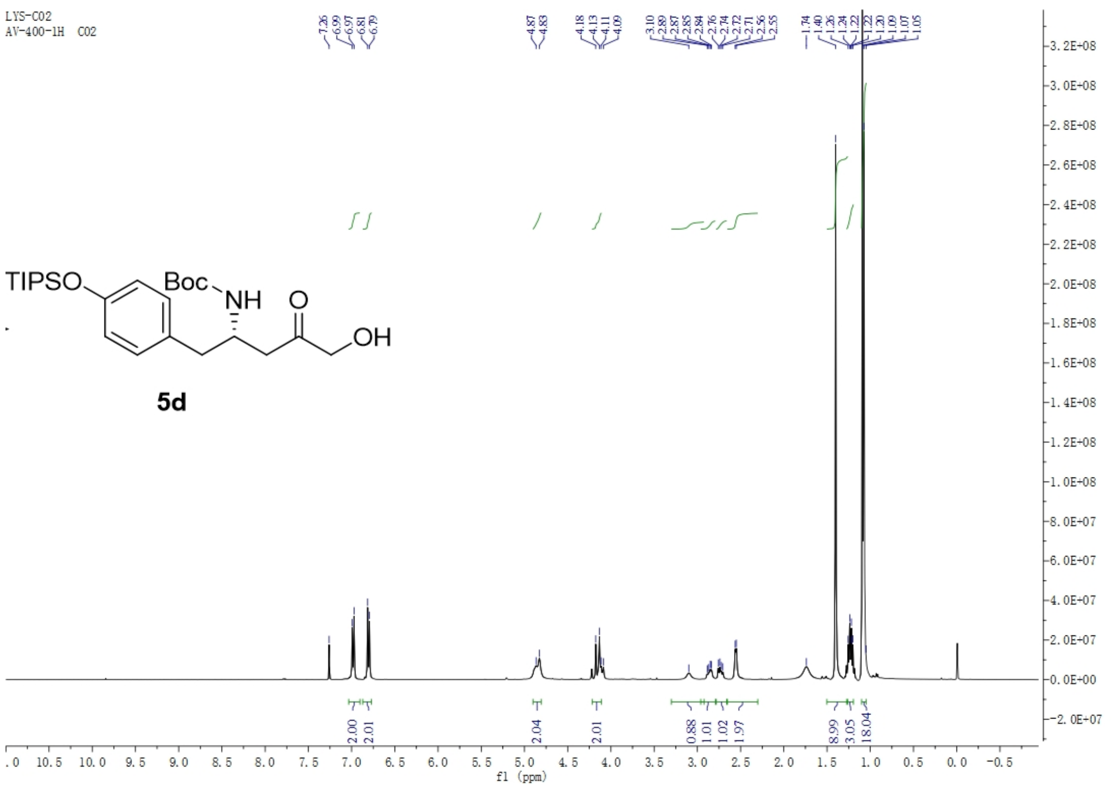

Figure S21. ^{1}H NMR of **5d** ($CDCl_3$, 400 MHz)

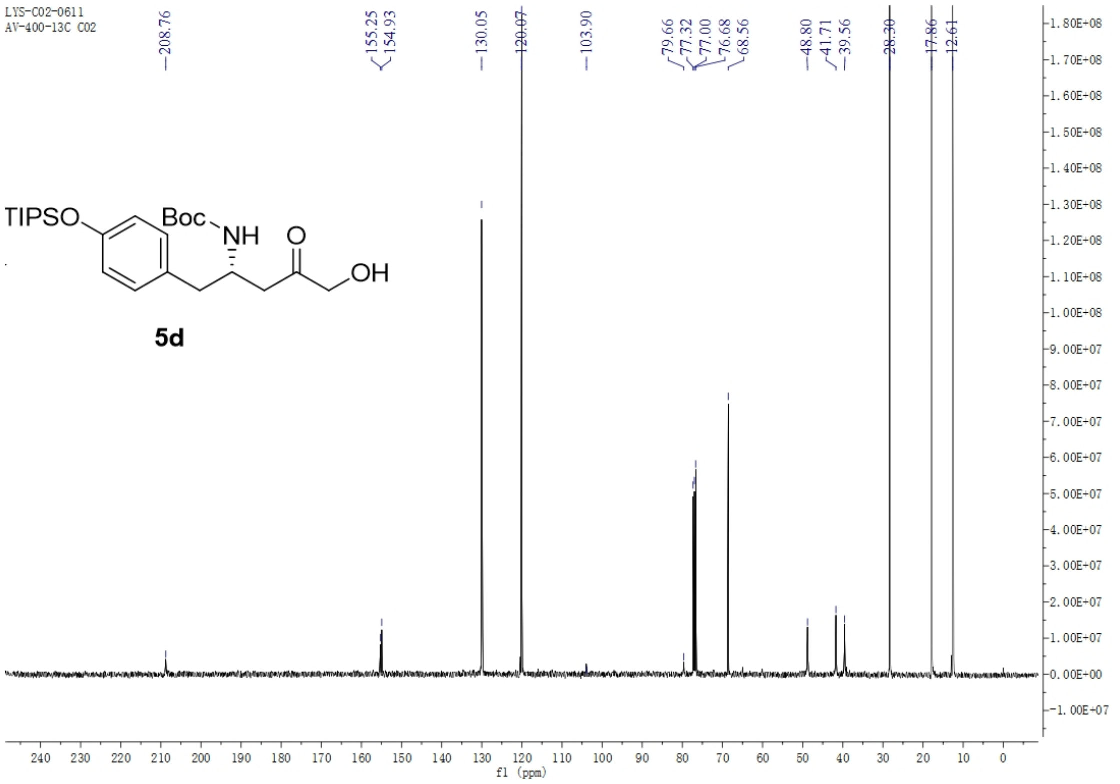

Figure S22. ^{13}C NMR of **5d** ($CDCl_3$, 100 MHz)

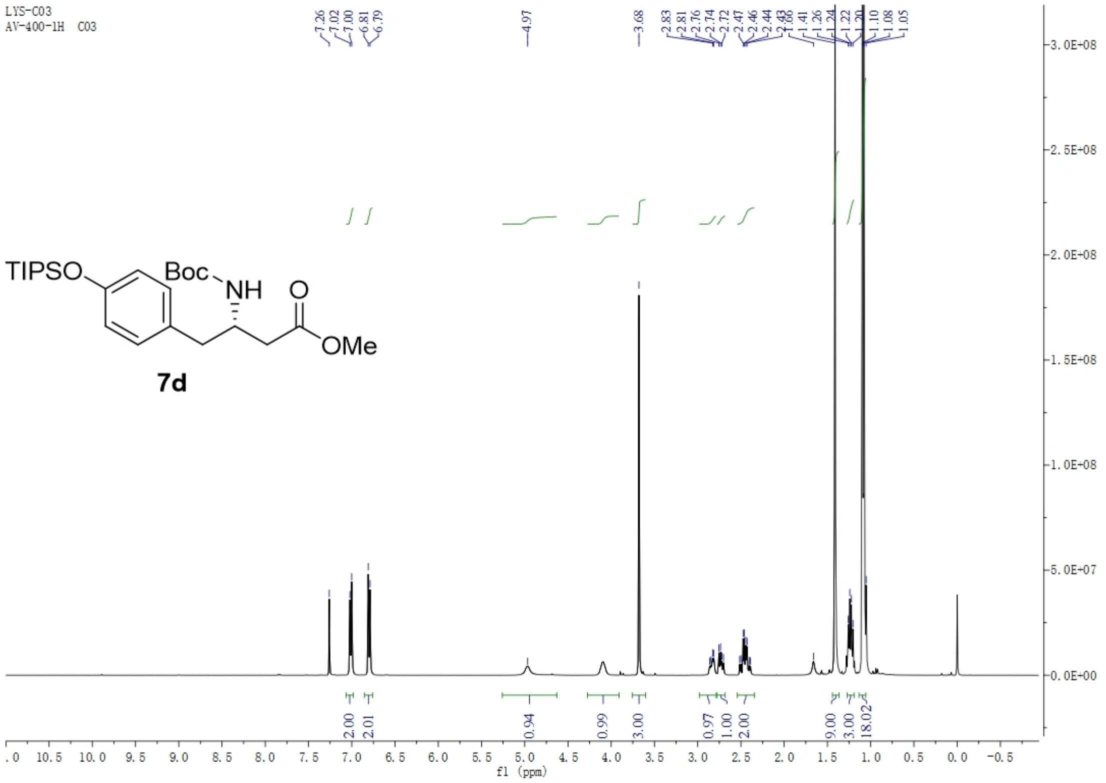

Figure S23. ^{1}H NMR of **7d** ($CDCl_3$, 400 MHz)

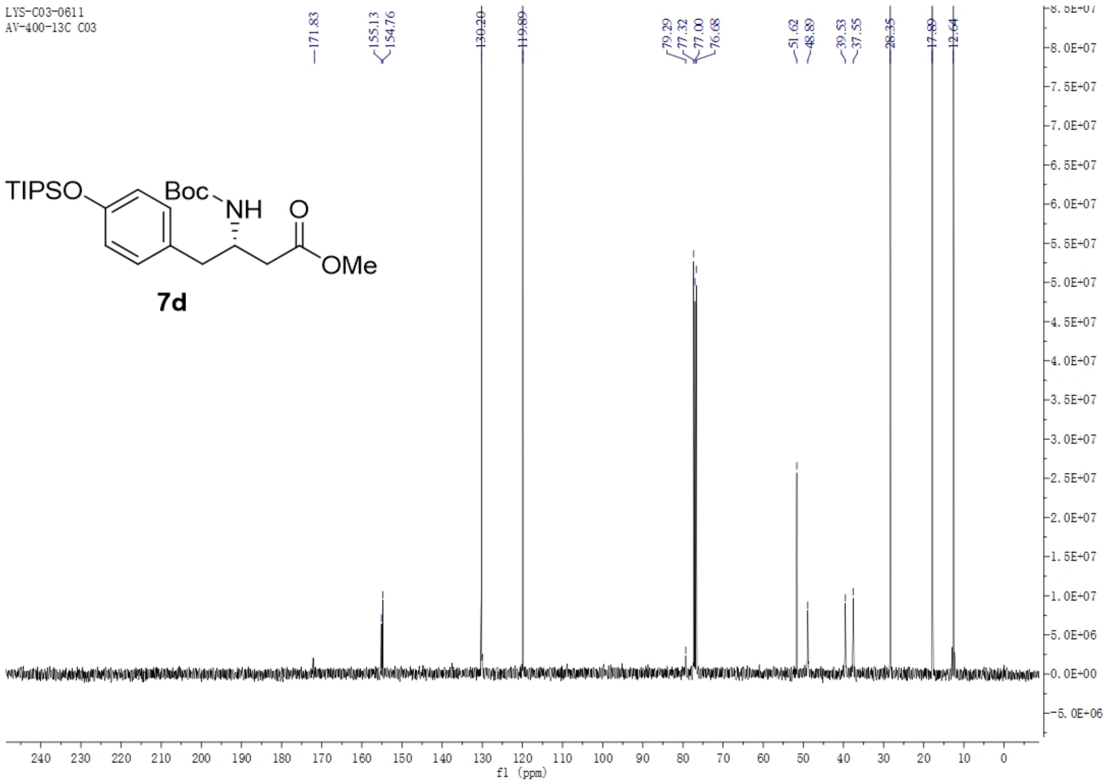

Figure S24. ^{13}C NMR of **7d** ($CDCl_3$, 100 MHz)

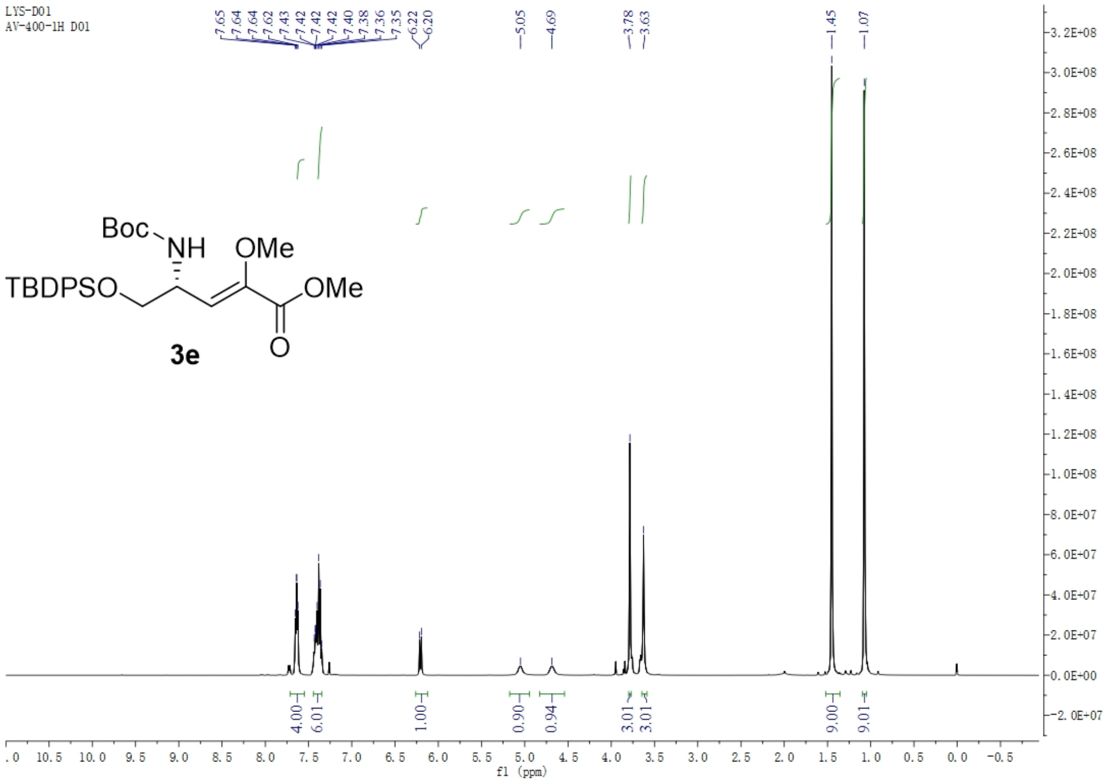

Figure S25. ^{1}H NMR of **3e** ($CDCl_3$, 400 MHz)

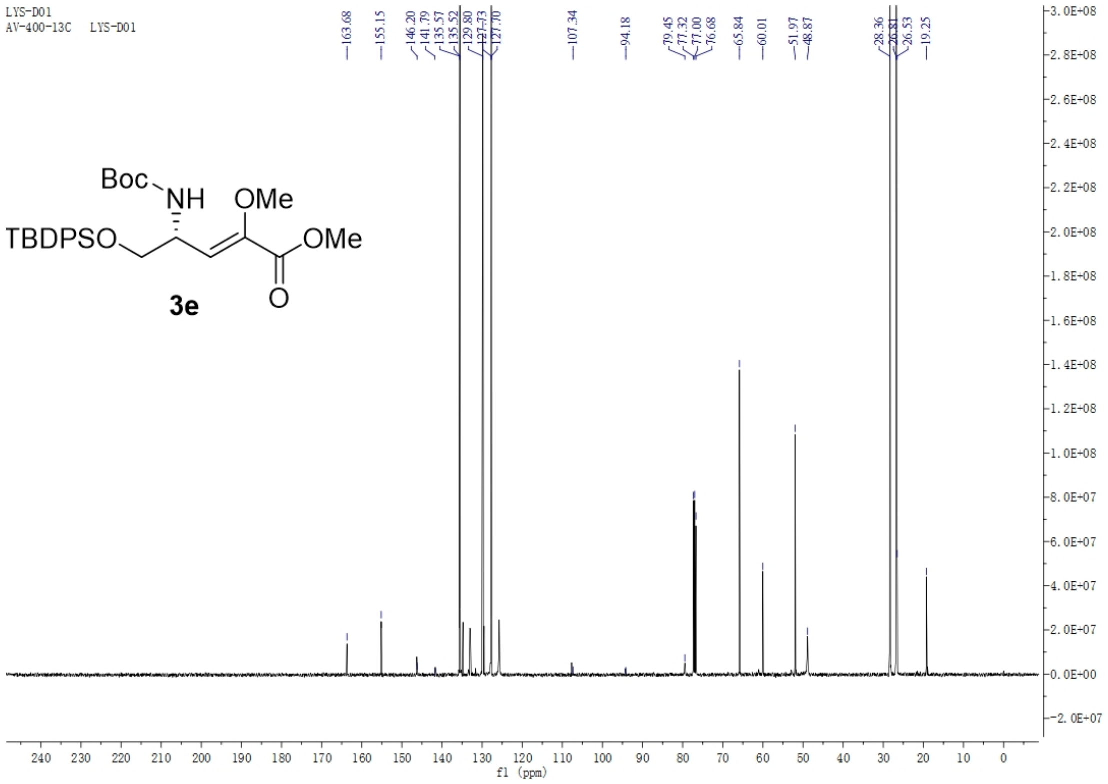

Figure S26. ^{13}C NMR of **3e** ($CDCl_3$, 100 MHz)

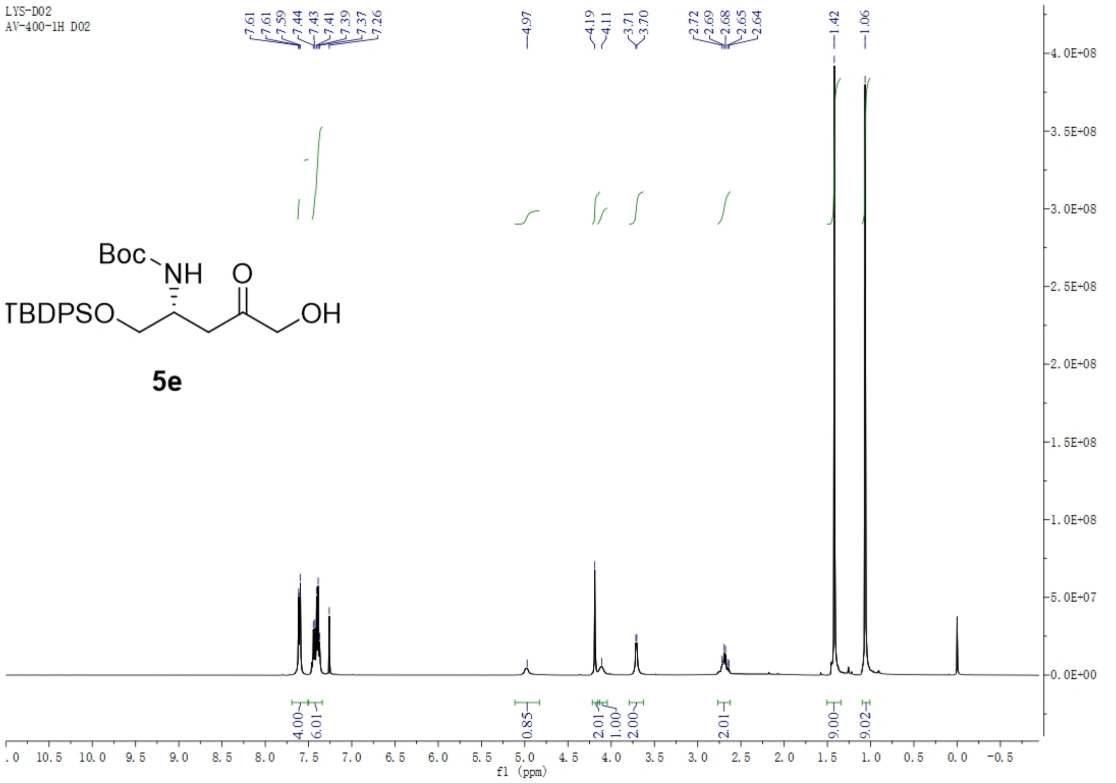

Figure S27. ^{1}H NMR of **5e** ($CDCl_3$, 400 MHz)

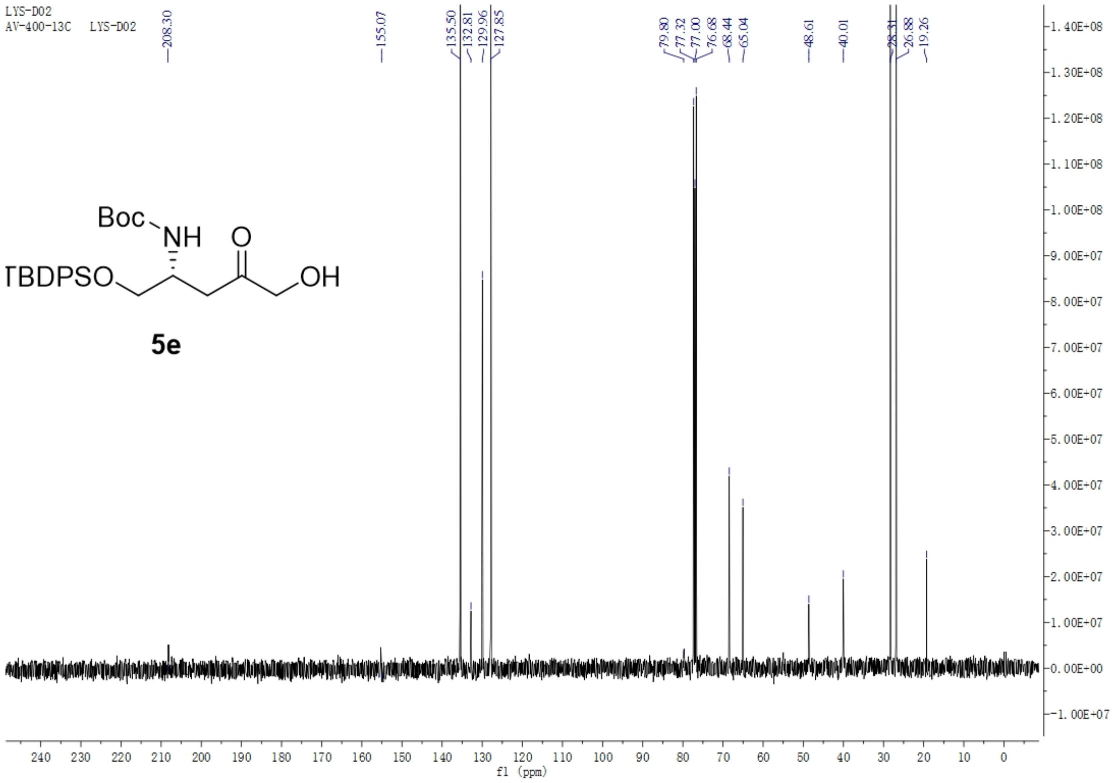

Figure S28. ^{13}C NMR of **5e** ($CDCl_3$, 100 MHz)

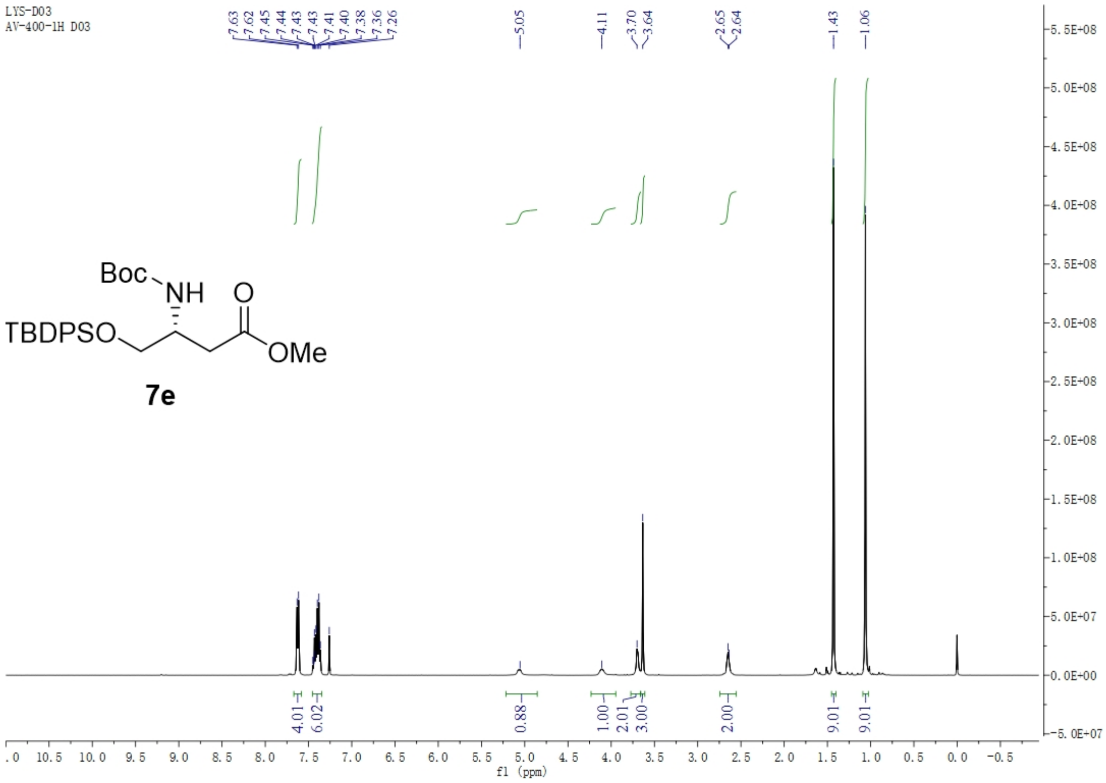

Figure S29. ^{1}H NMR of **7e** ($CDCl_3$, 400 MHz)

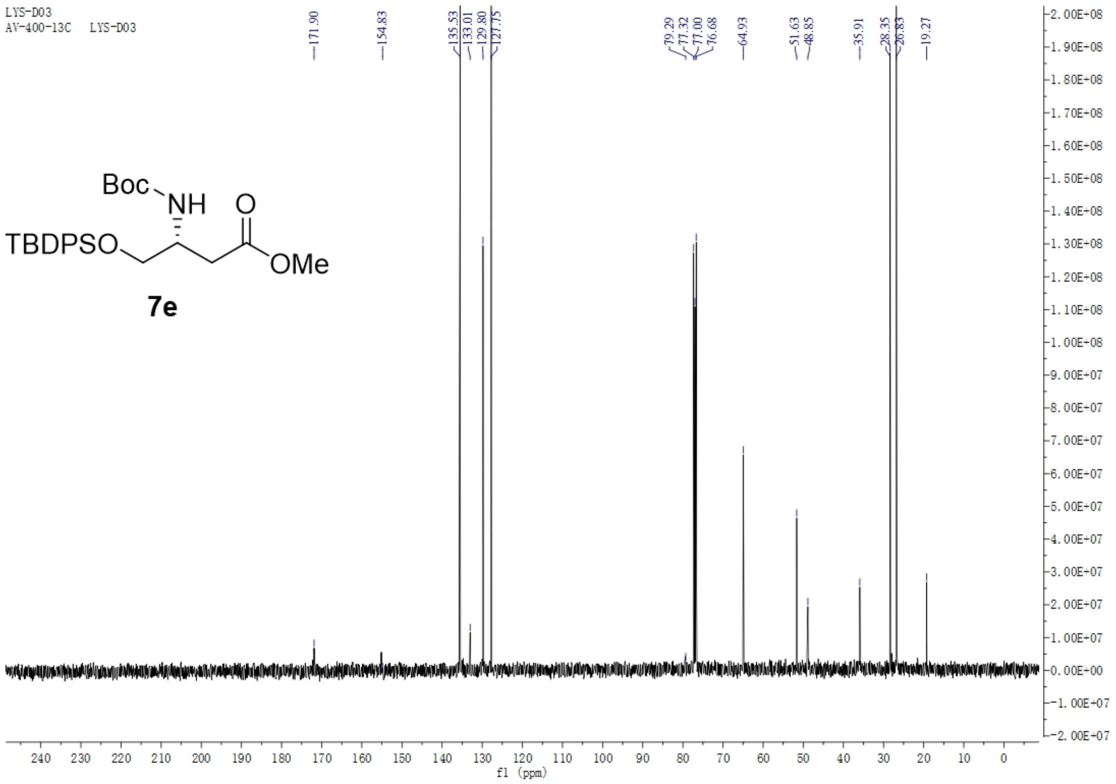

Figure S30. ^{13}C NMR of **7e** ($CDCl_3$, 100 MHz)

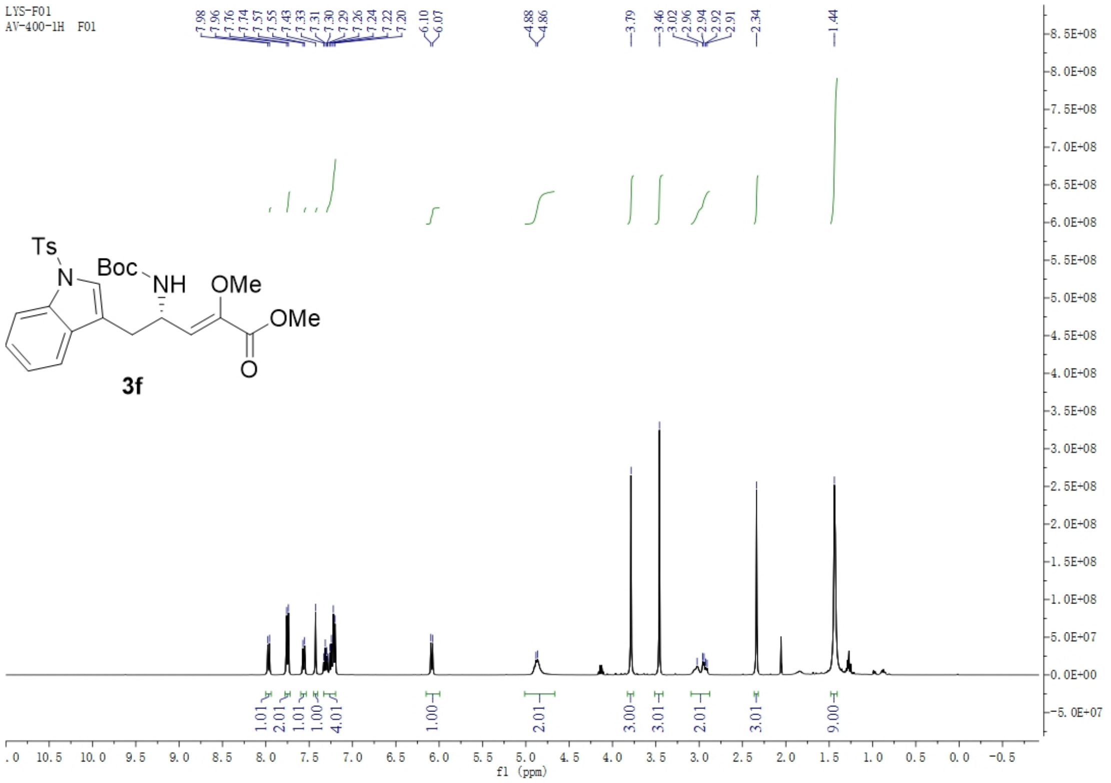

Figure S31. ^{1}H NMR of **3f** ($CDCl_3$, 400 MHz)

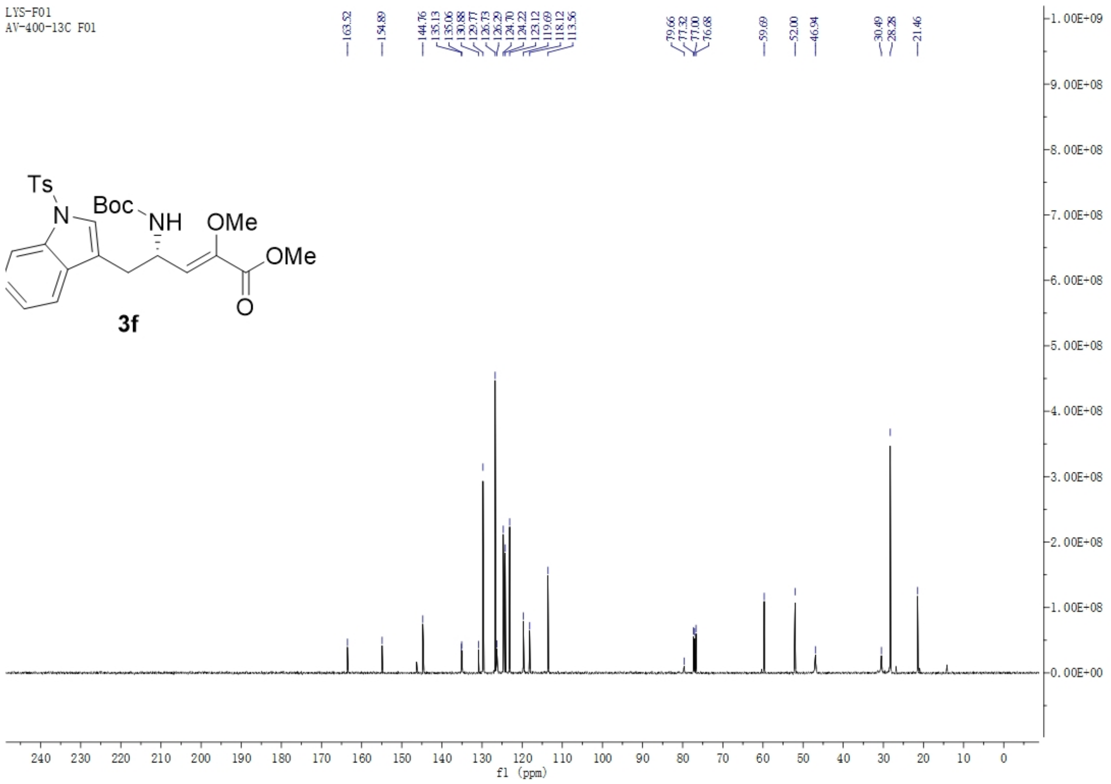

Figure S32. ^{13}C NMR of **3f** ($CDCl_3$, 100 MHz)

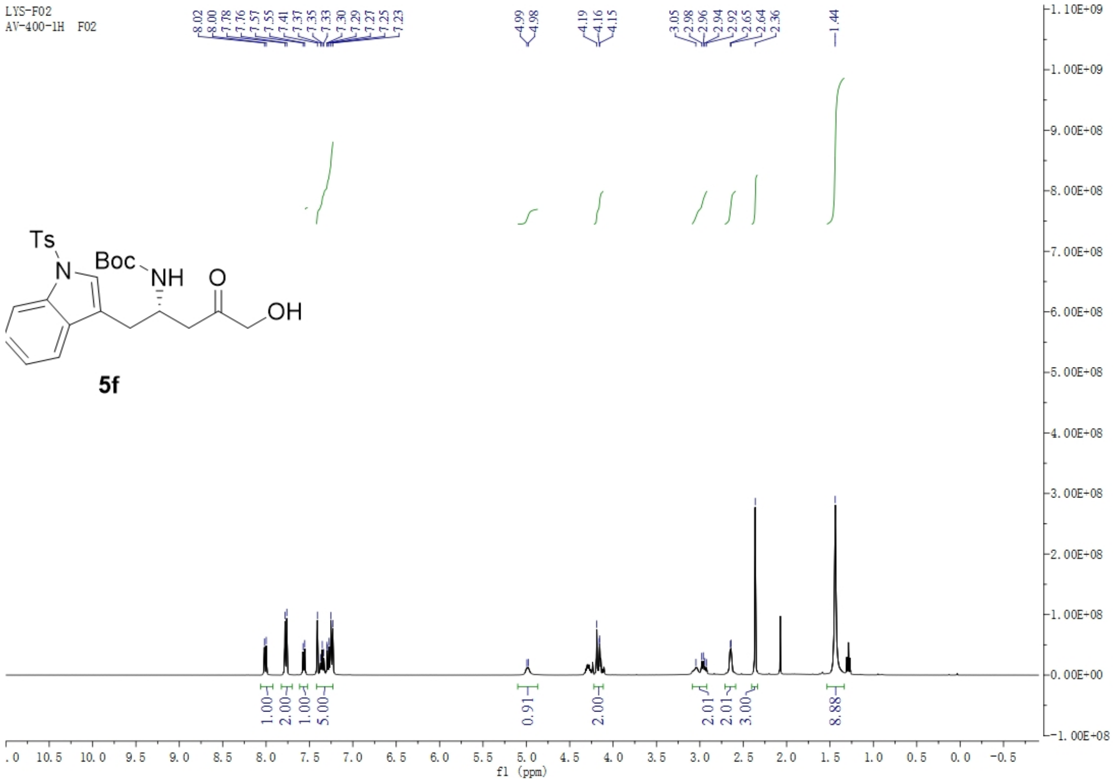

Figure S33. ^{1}H NMR of **5f** ($CDCl_3$, 400 MHz)

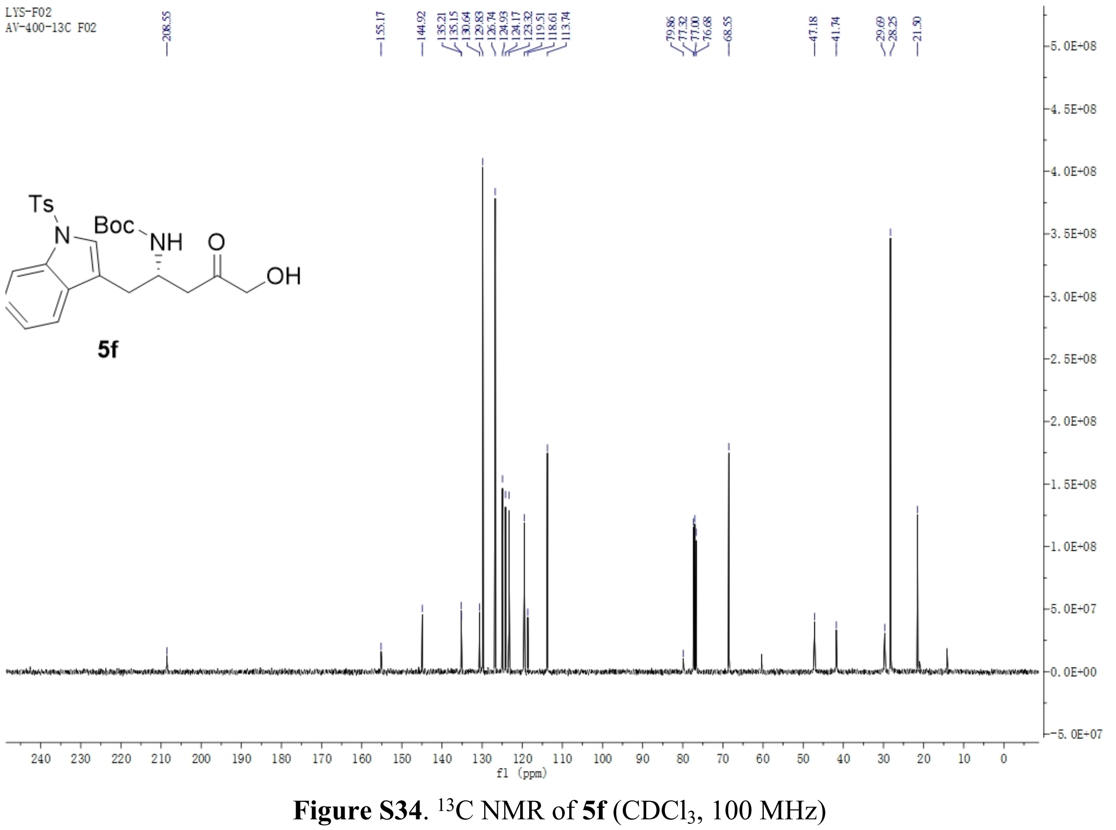

Figure S34. ^{13}C NMR of **5f** ($CDCl_3$, 100 MHz)

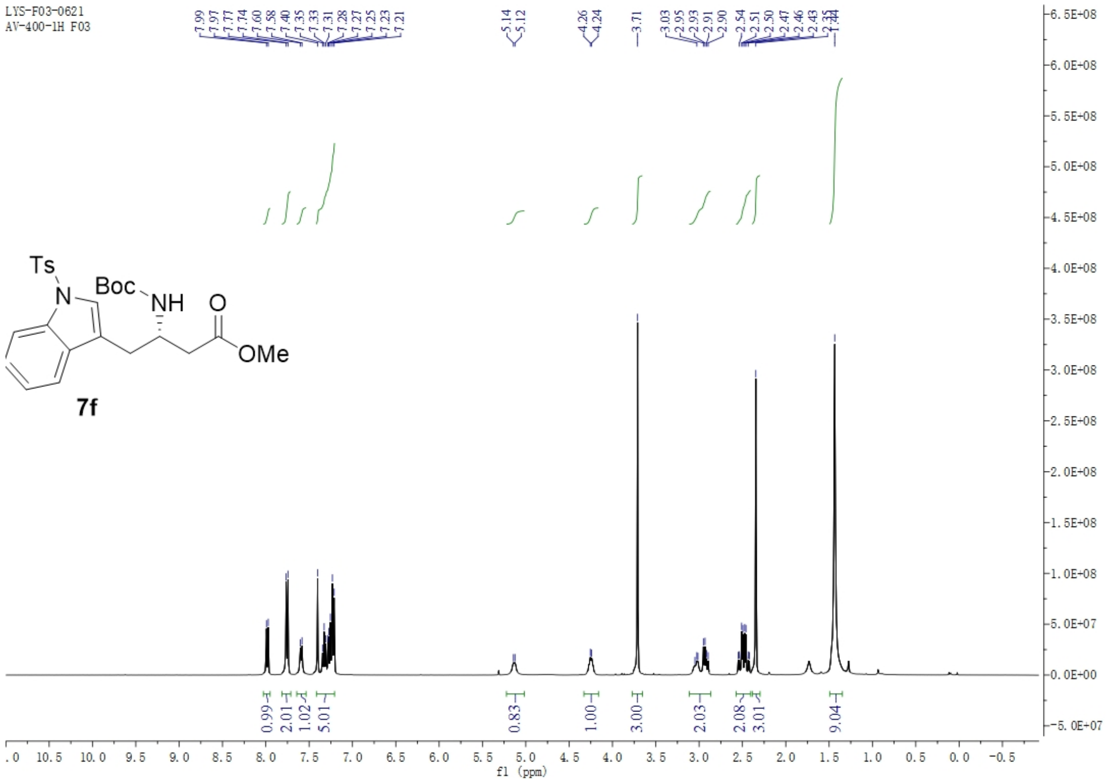

Figure S35. ^{1}H NMR of **7f** ($CDCl_3$, 400 MHz)

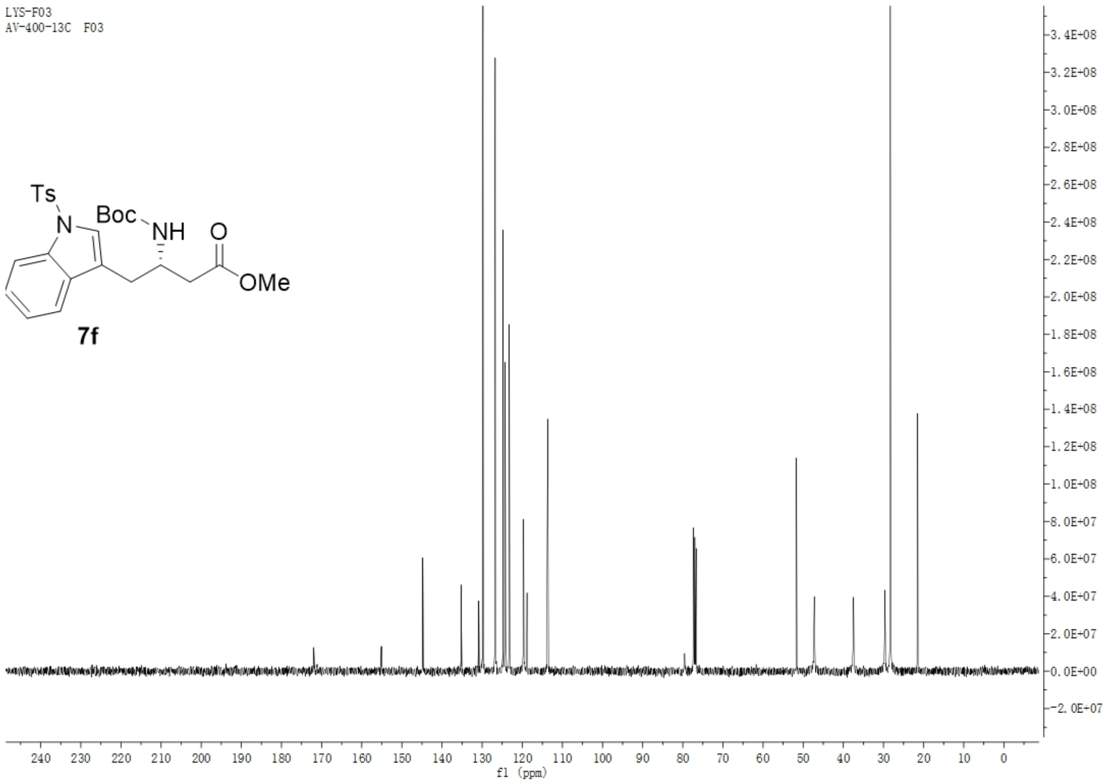

Figure S36. ^{13}C NMR of **7f** ($CDCl_3$, 100 MHz)

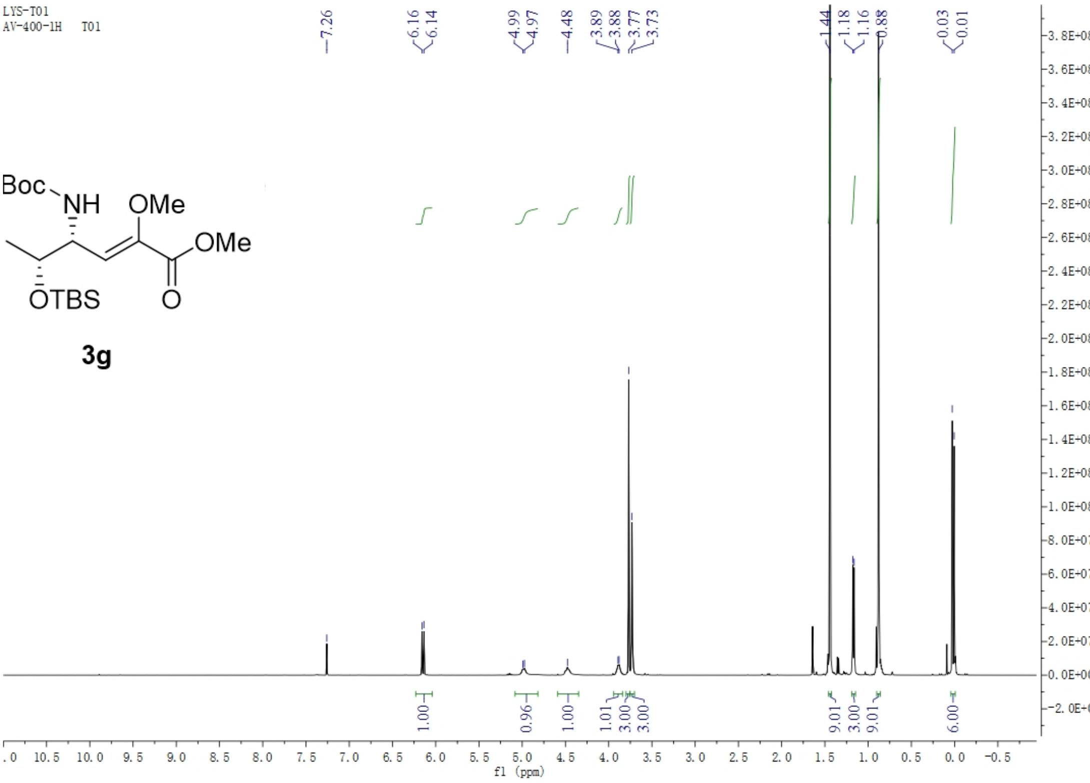

Figure S37. ^{1}H NMR of **3g** ($CDCl_3$, 400 MHz)

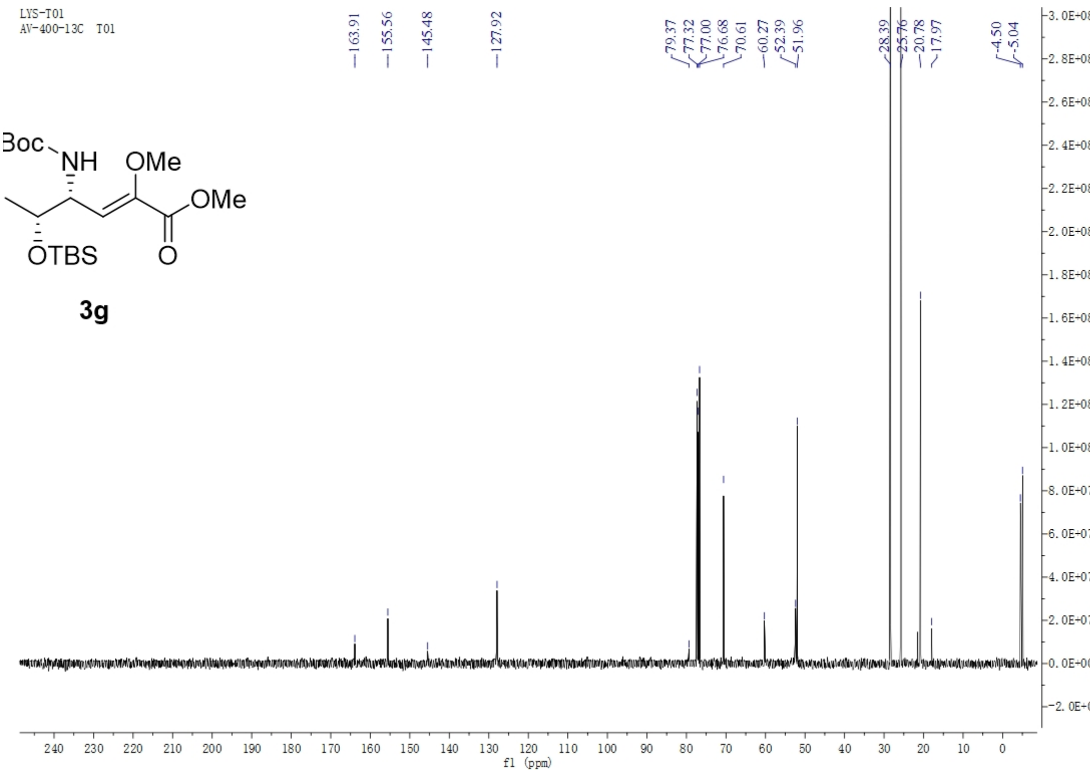

Figure S38. ^{13}C NMR of **3g** ($CDCl_3$, 100 MHz)

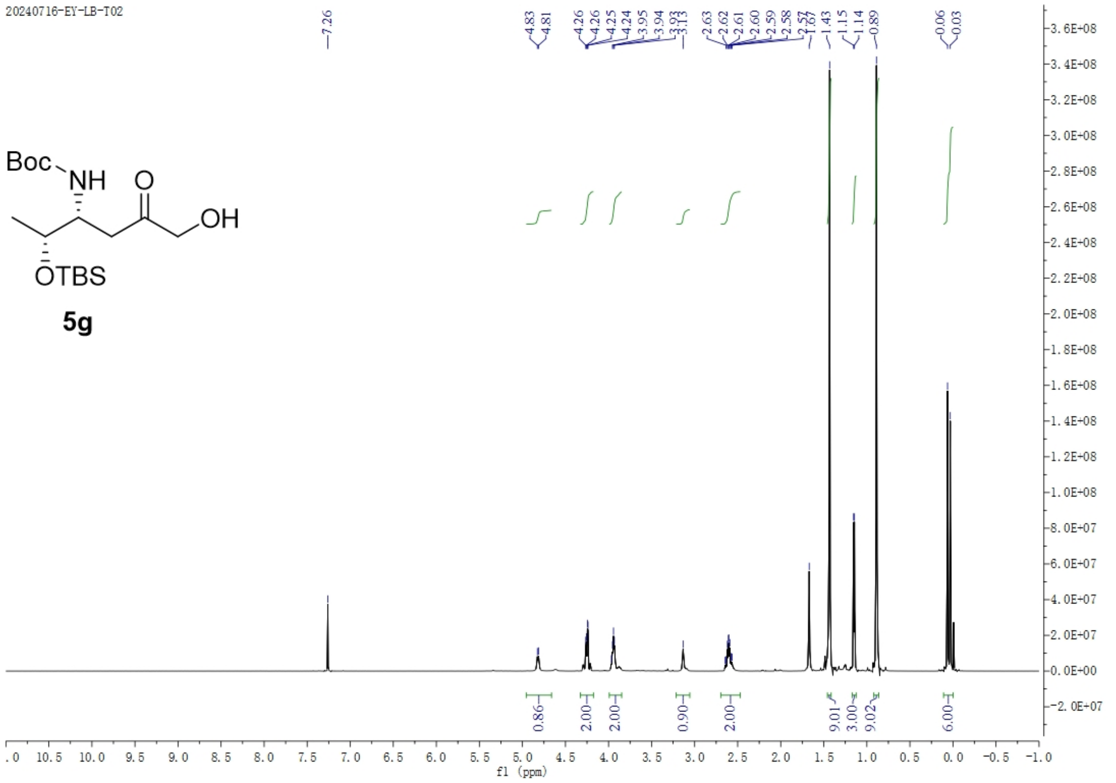

Figure S39. ^{1}H NMR of **5g** ($CDCl_3$, 600 MHz)

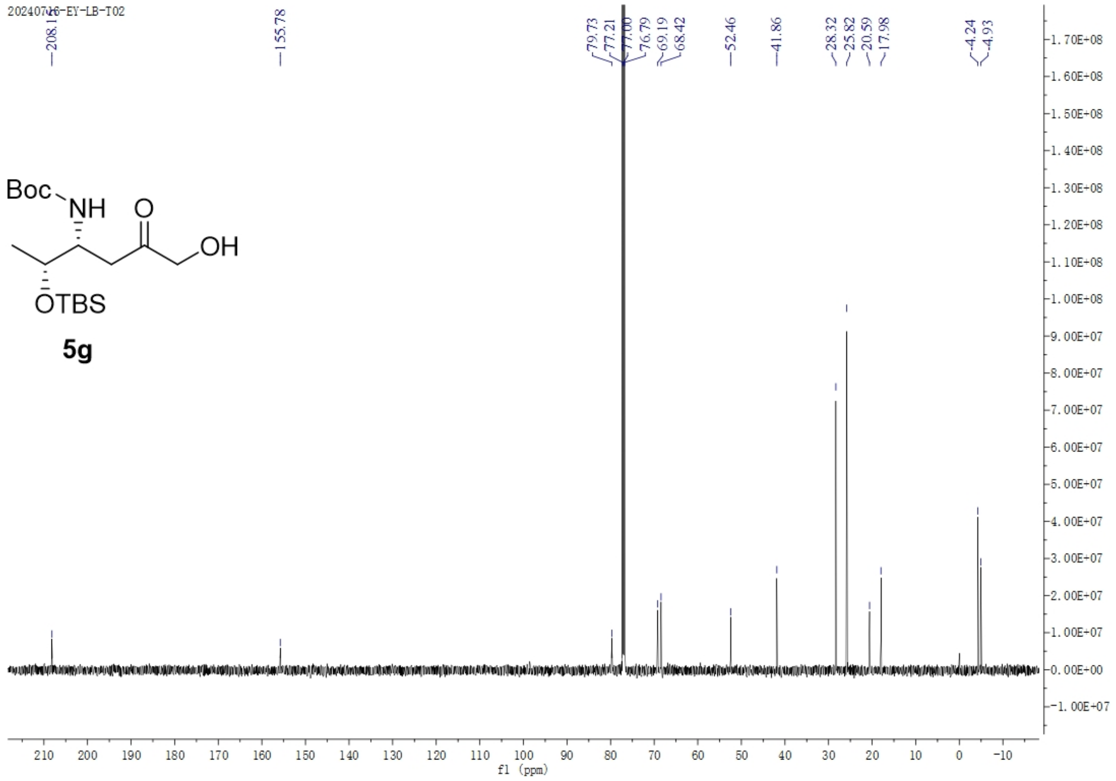

Figure S40. ^{13}C NMR of **5g** ($CDCl_3$, 150 MHz)

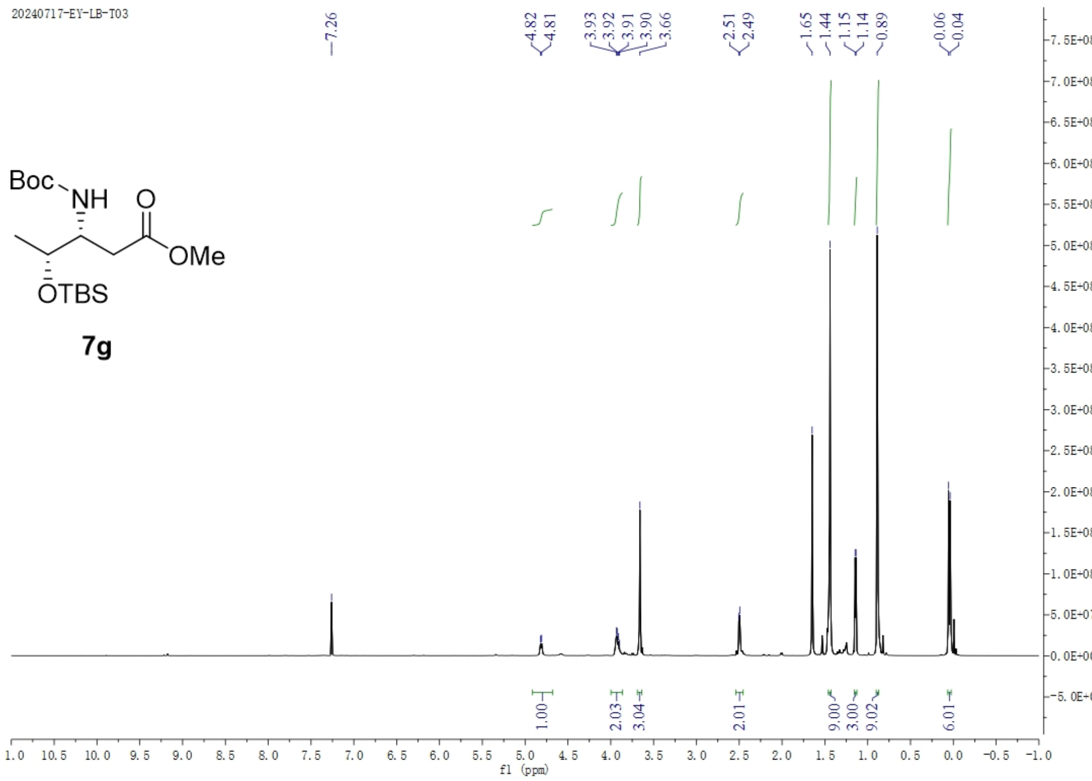

Figure S41. ^{1}H NMR of **7g** ($CDCl_3$, 600 MHz)

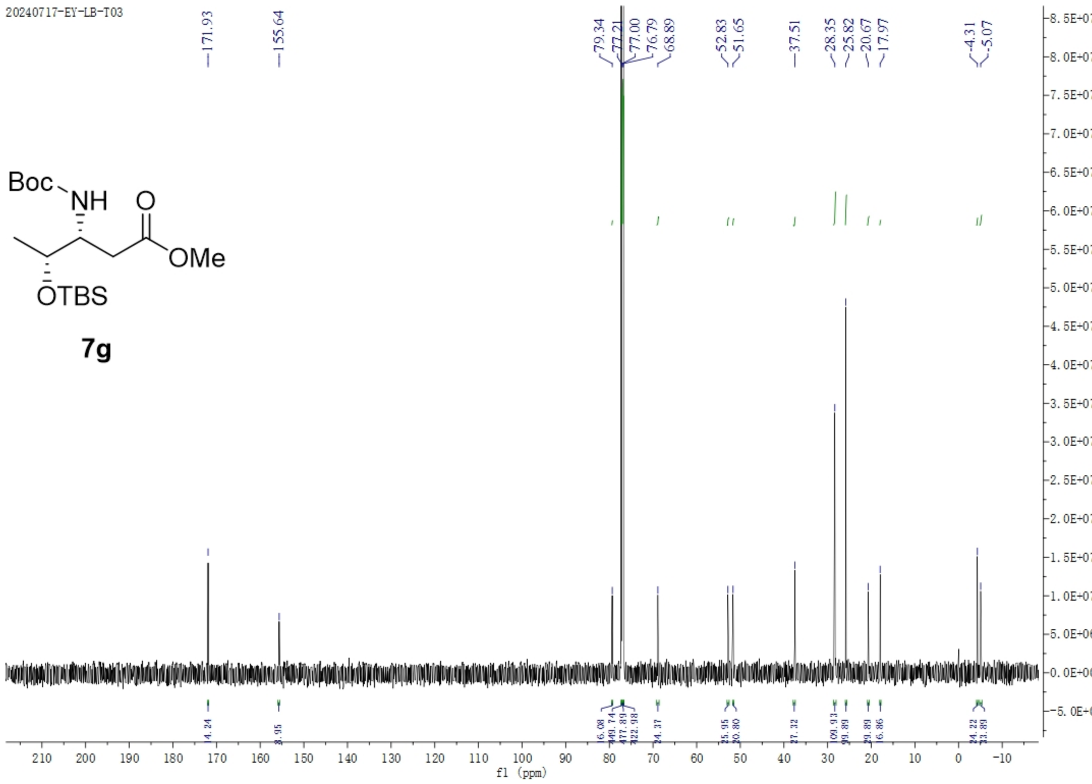

Figure S42. ^{13}C NMR of **7g** ($CDCl_3$, 150 MHz)

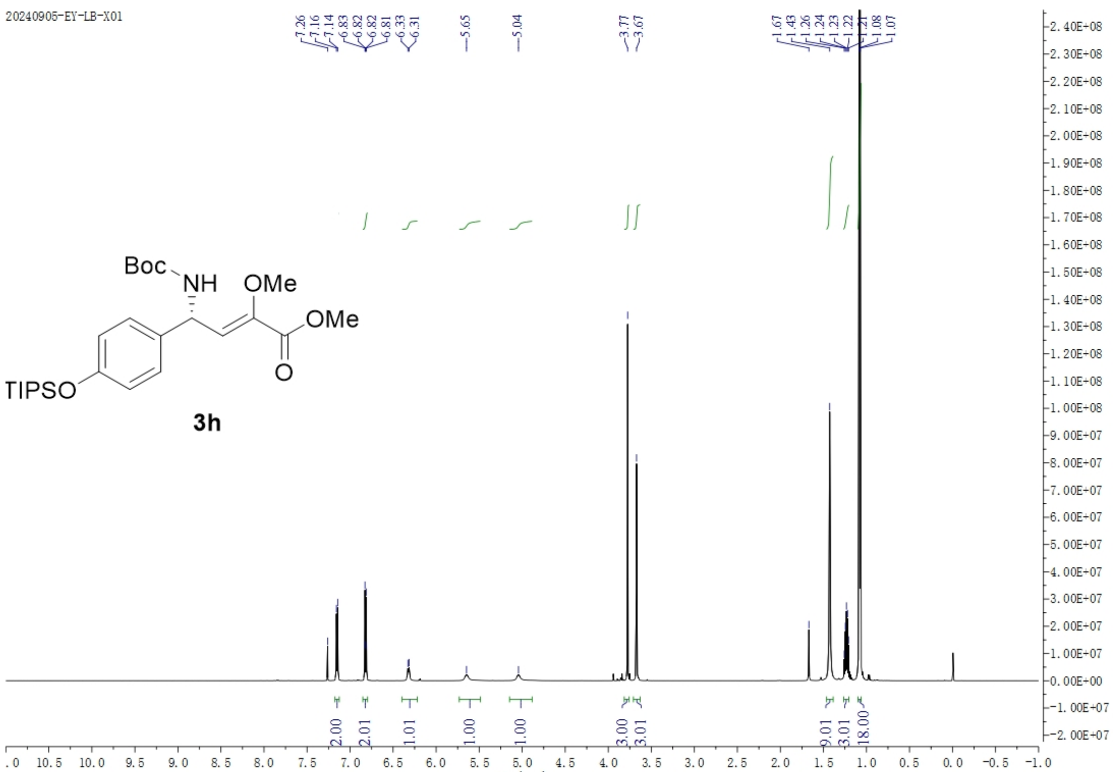

Figure S43. ^{1}H NMR of **3h** ($CDCl_3$, 600 MHz)

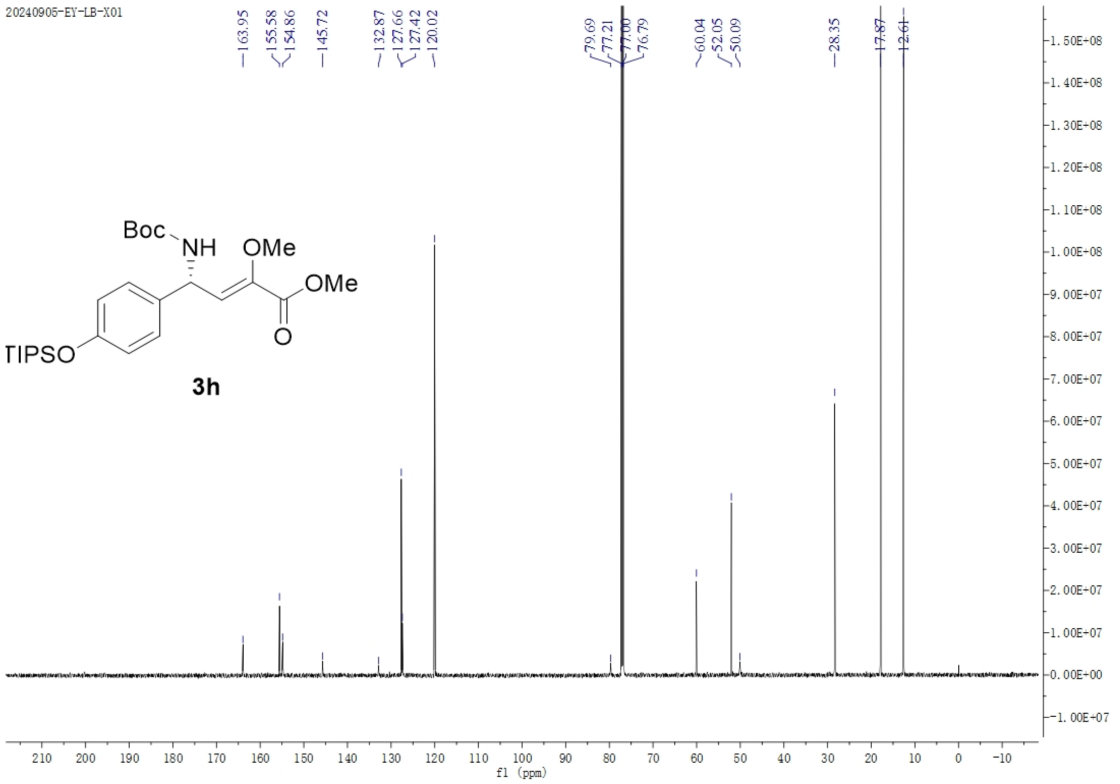

Figure S44. ^{13}C NMR of **3h** ($CDCl_3$, 150 MHz)

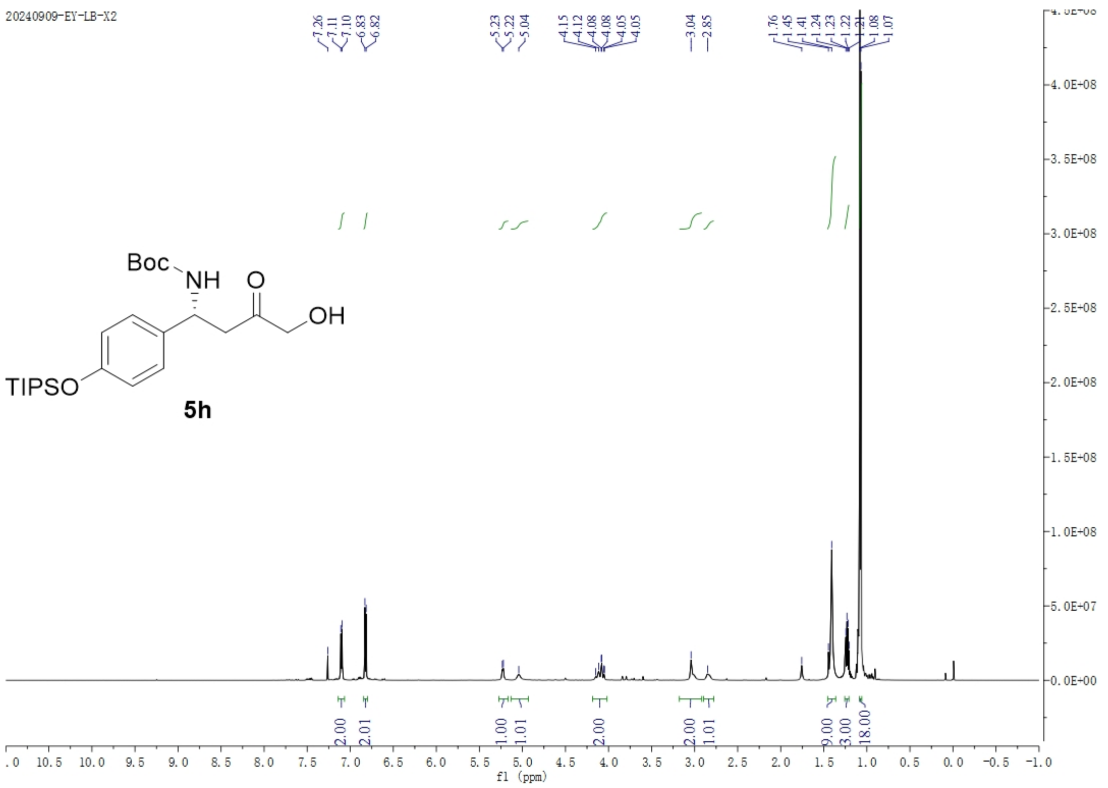

Figure S45. ^{1}H NMR of **5h** ($CDCl_3$, 600 MHz)

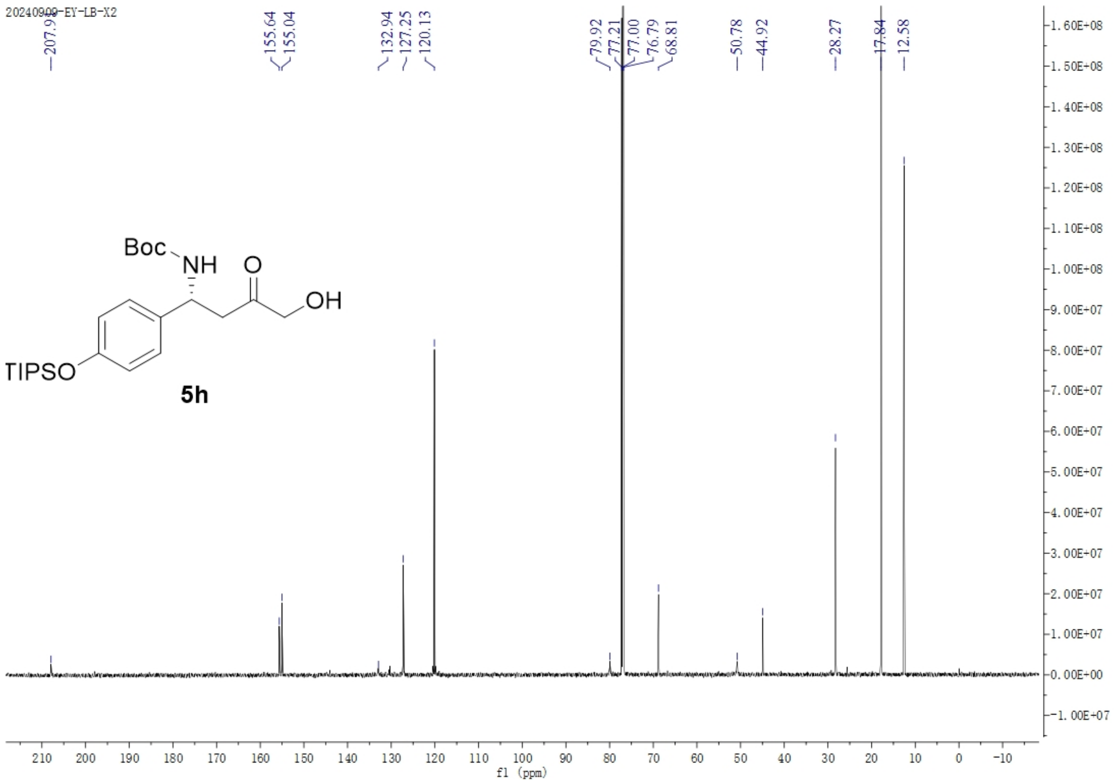

Figure S46. ^{13}C NMR of **5h** ($CDCl_3$, 150 MHz)

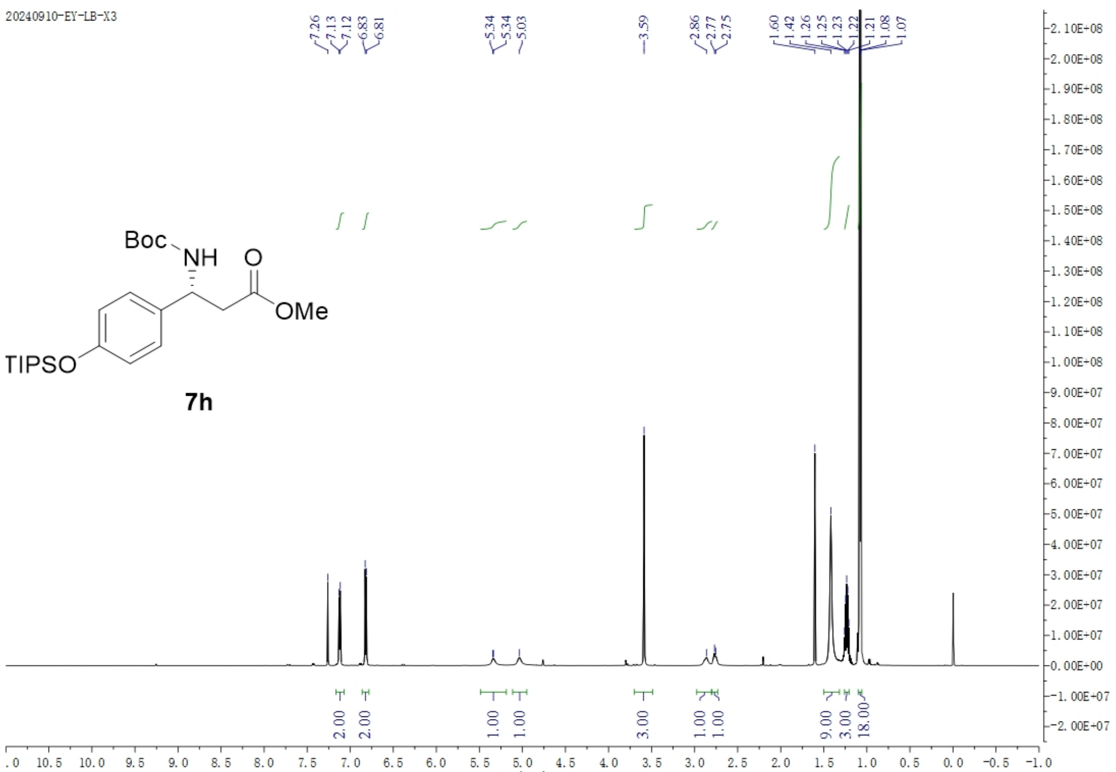

Figure S47. ^{1}H NMR of **7h** ($CDCl_3$, 600 MHz)

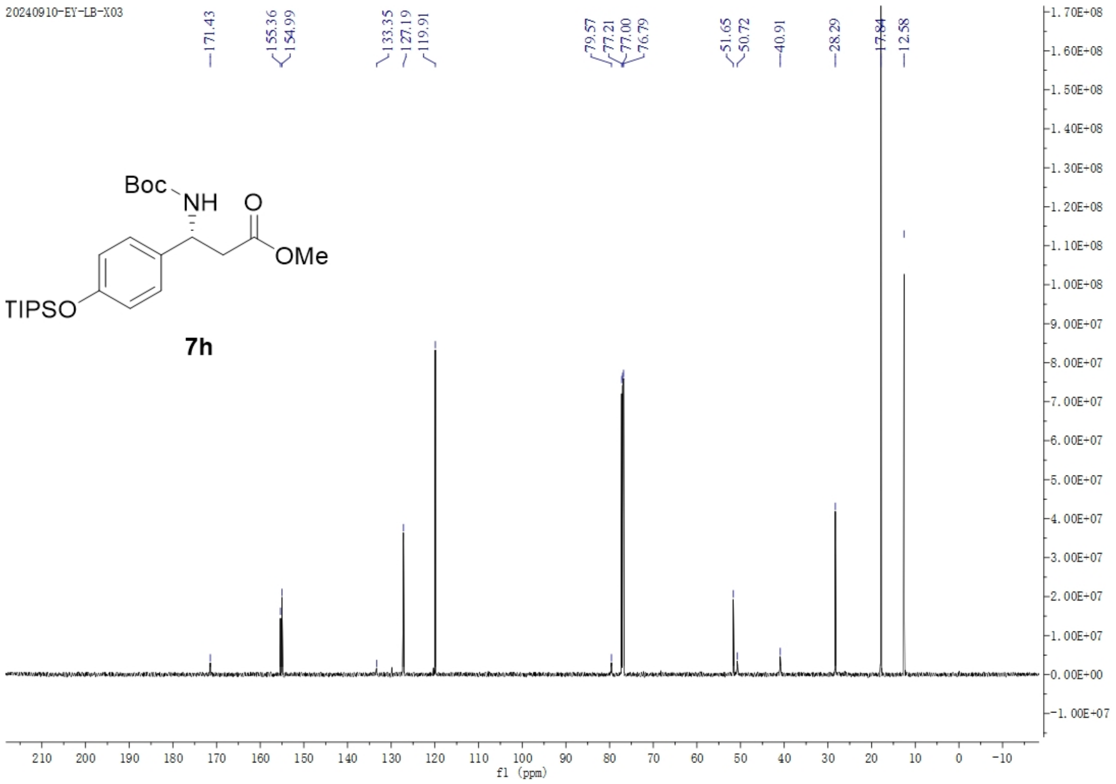

Figure S48. ^{13}C NMR of **7h** ($CDCl_3$, 150 MHz)

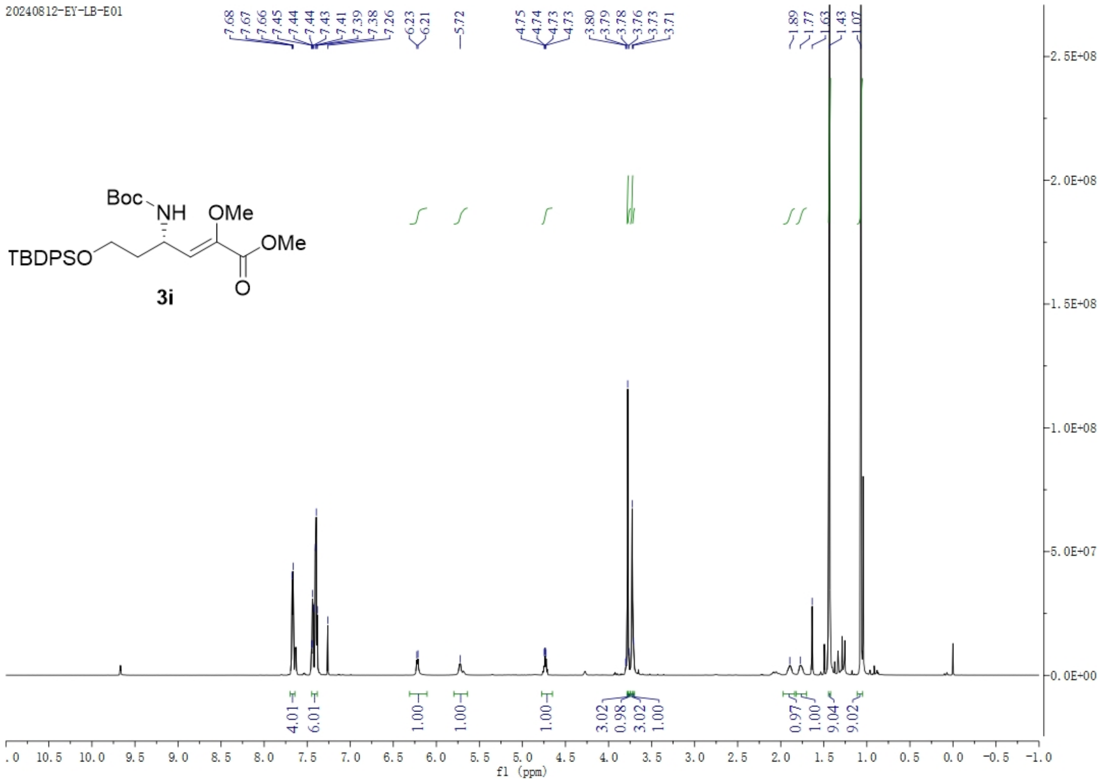

Figure S49. ^{1}H NMR of **3i** ($CDCl_3$, 600 MHz)

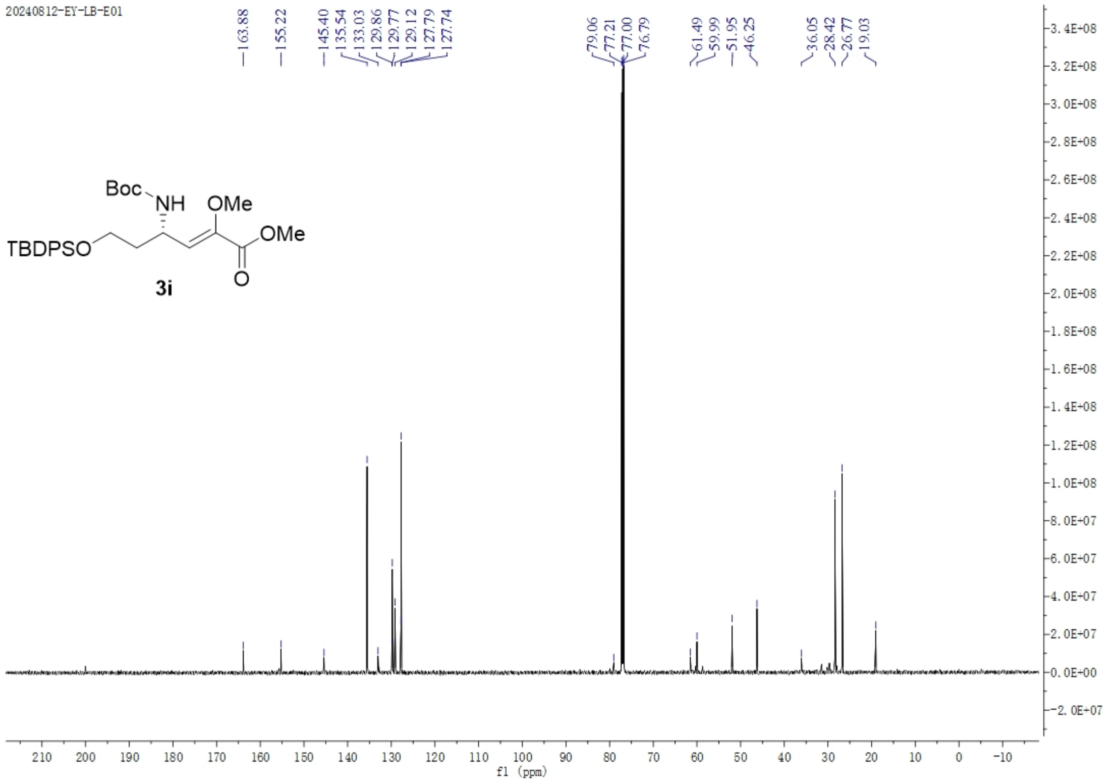

Figure S50. ^{13}C NMR of **3i** ($CDCl_3$, 150 MHz)

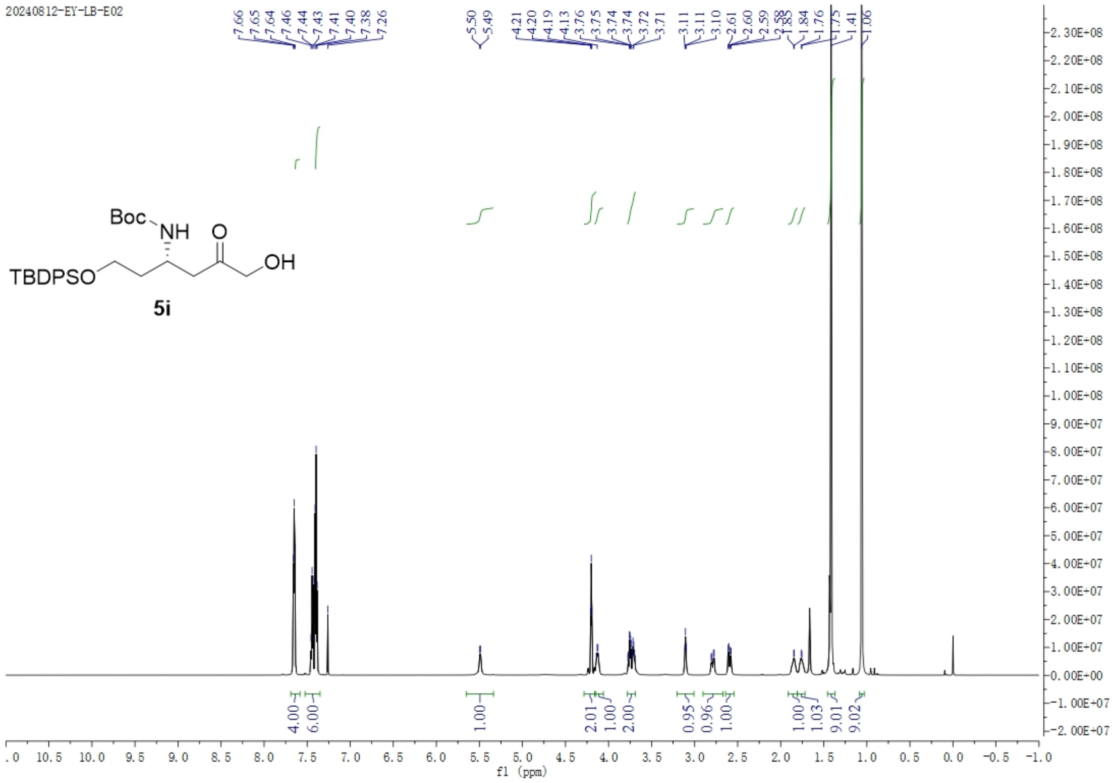

Figure S51. ^{1}H NMR of **5i** ($CDCl_3$, 600 MHz)

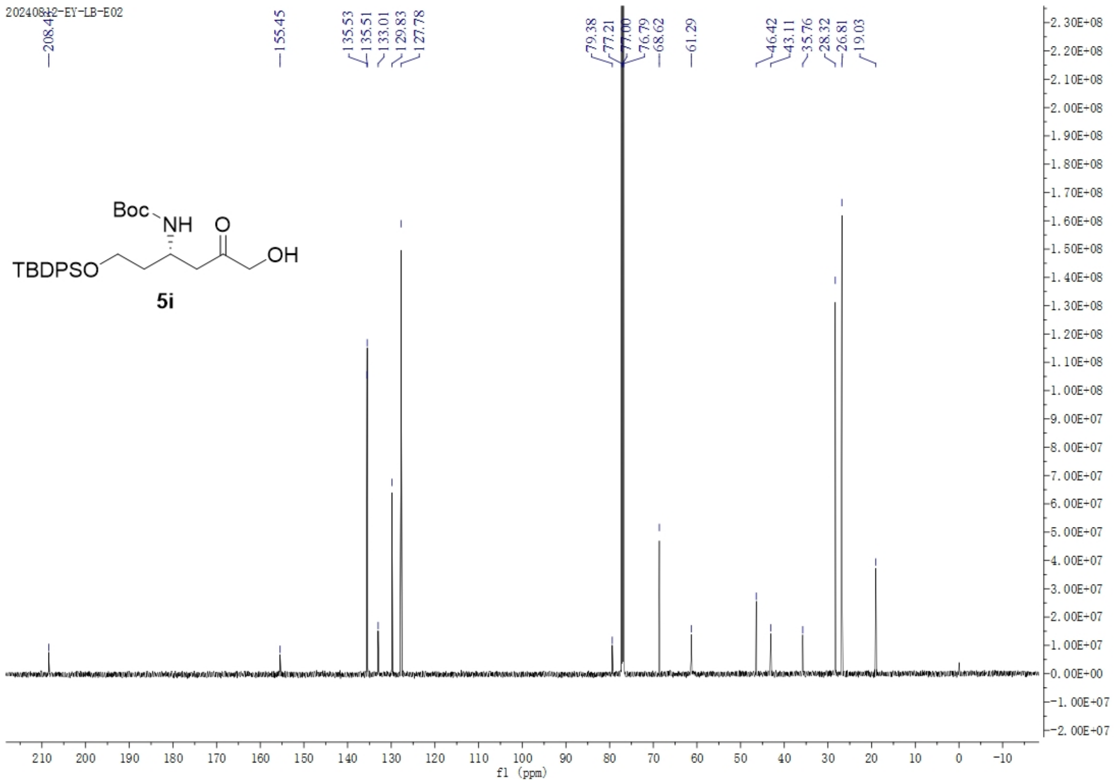

Figure S52. ^{13}C NMR of **5i** ($CDCl_3$, 150 MHz)

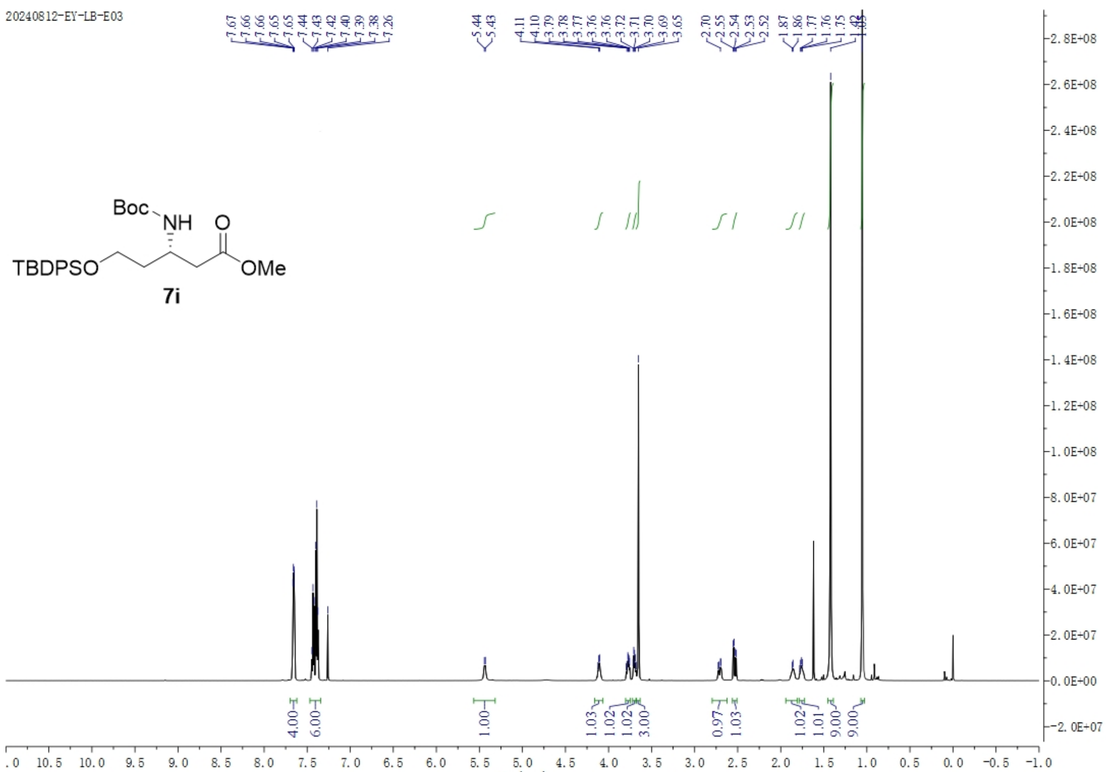

Figure S53. ^{1}H NMR of **7i** ($CDCl_3$, 600 MHz)

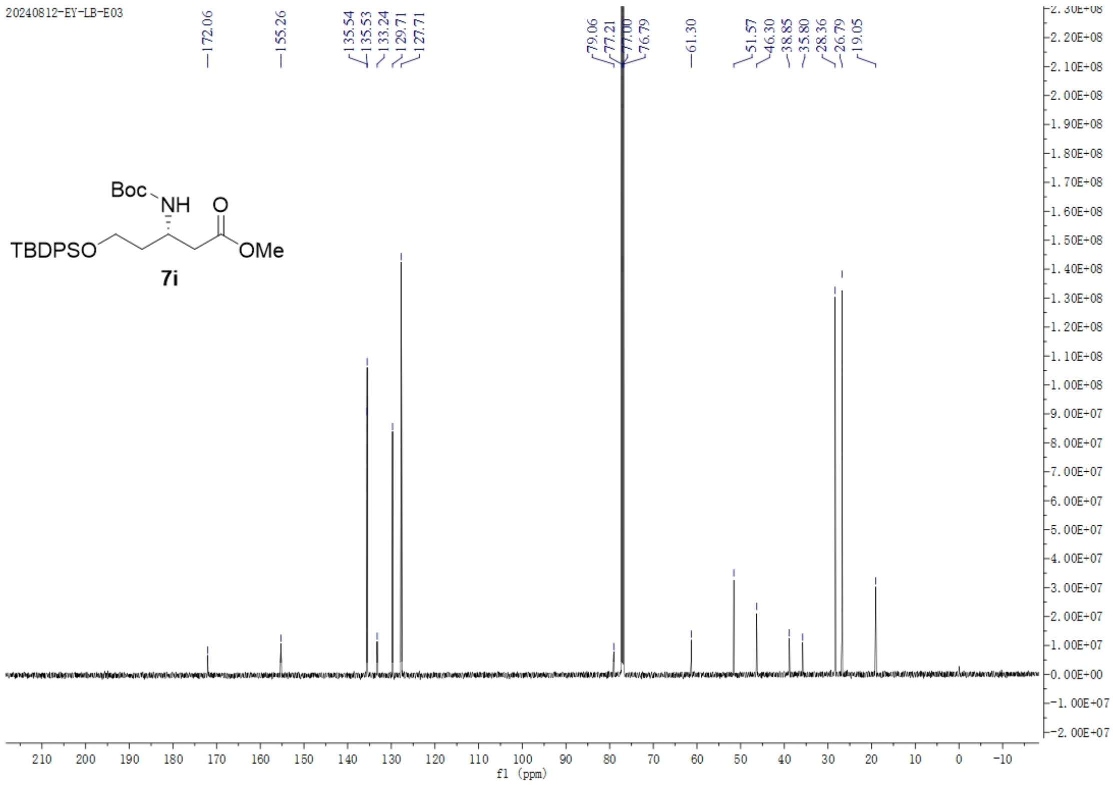

Figure S54. ^{13}C NMR of **7i** ($CDCl_3$, 150 MHz)

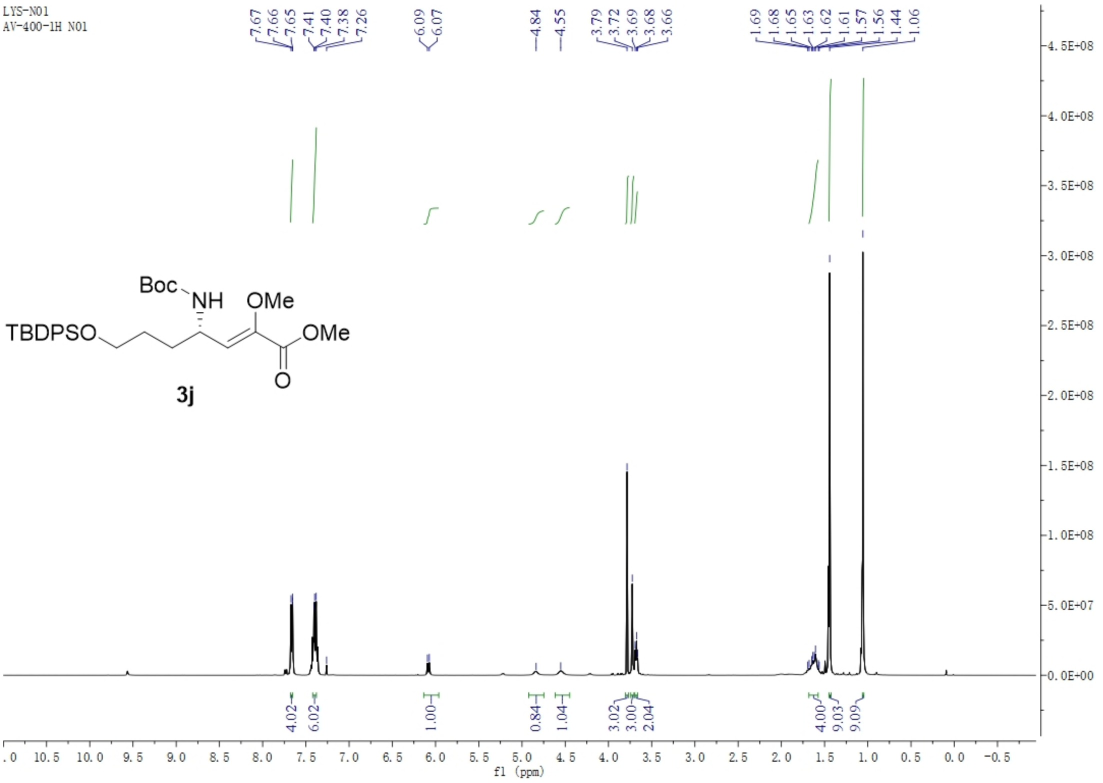

Figure S55. ^{1}H NMR of **3j** ($CDCl_3$, 600 MHz)

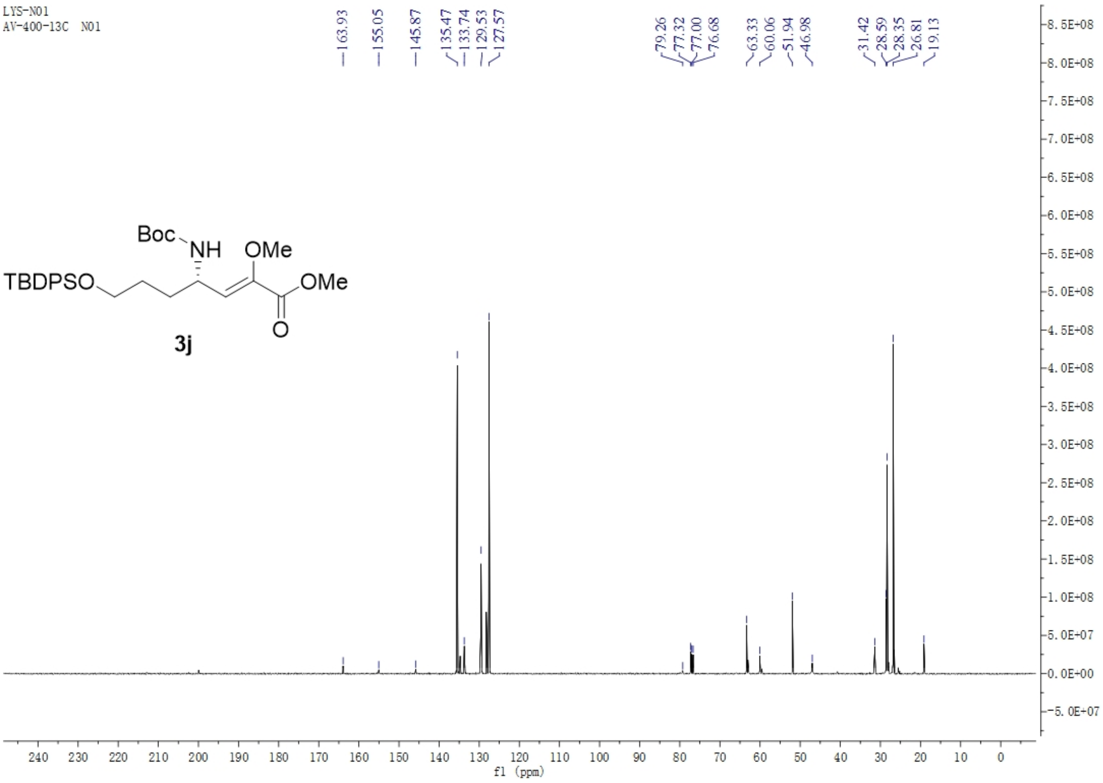

Figure S56. ^{13}C NMR of **3j** ($CDCl_3$, 150 MHz)

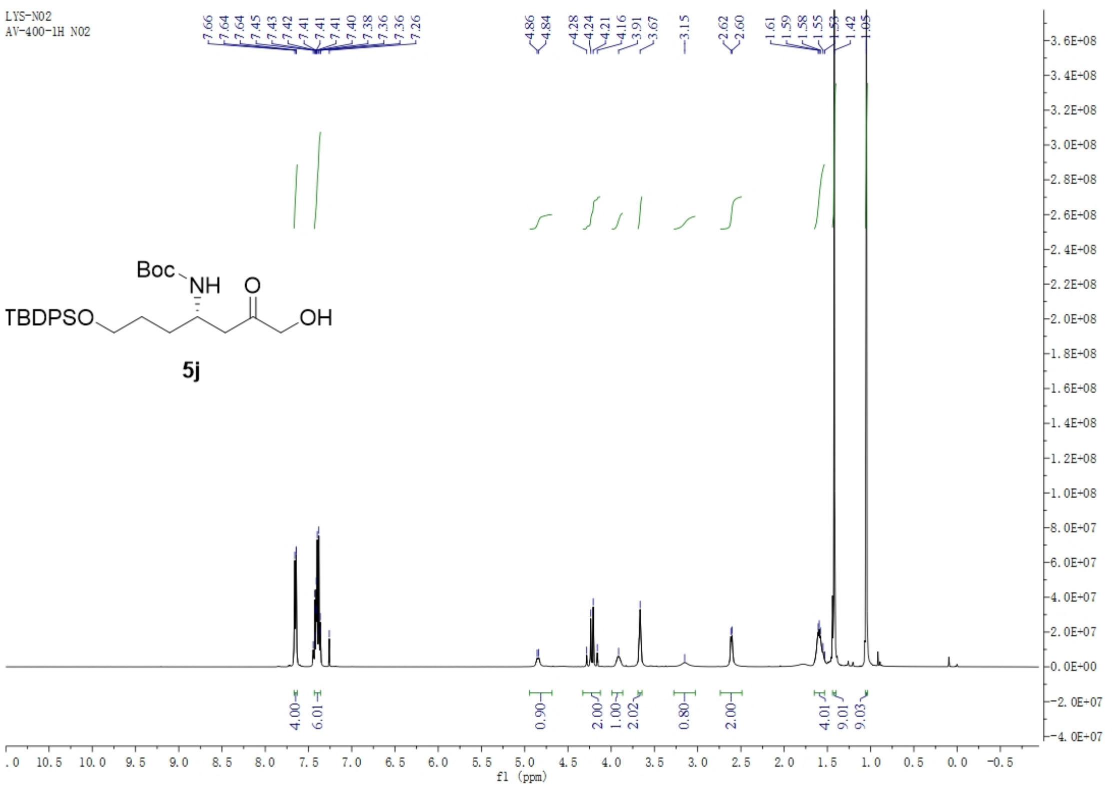

Figure S57. ^{1}H NMR of **5j** ($CDCl_3$, 600 MHz)

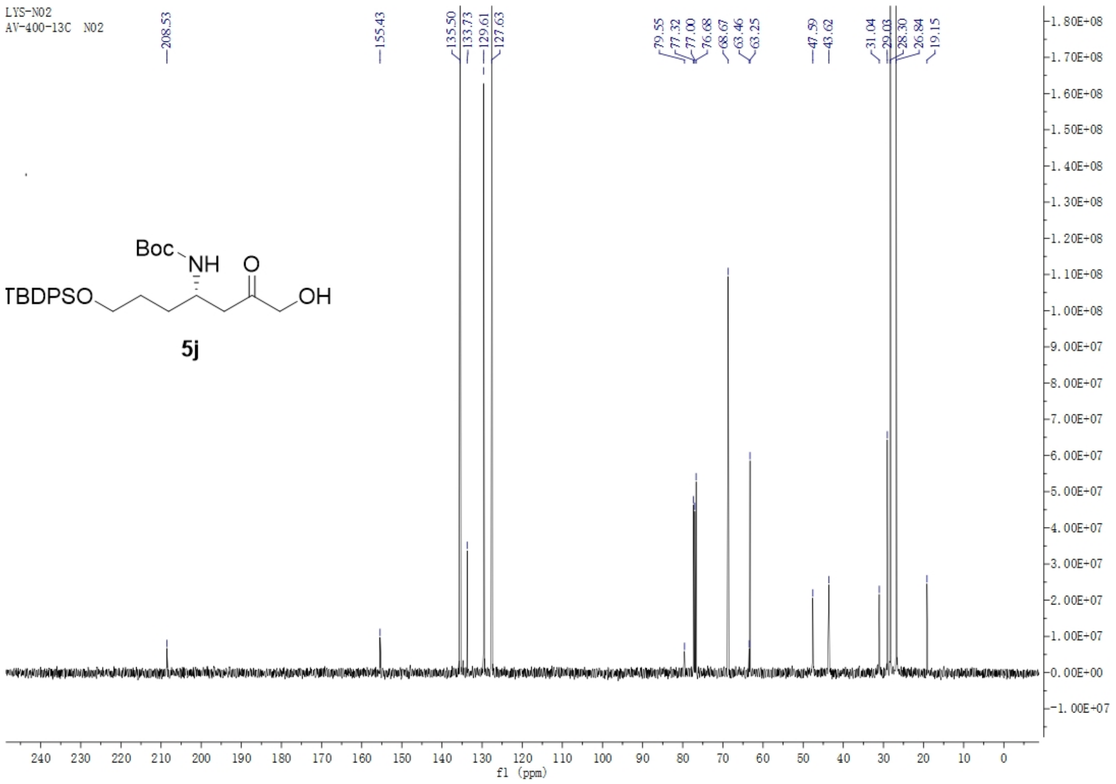

Figure S58. ^{13}C NMR of **5j** ($CDCl_3$, 150 MHz)

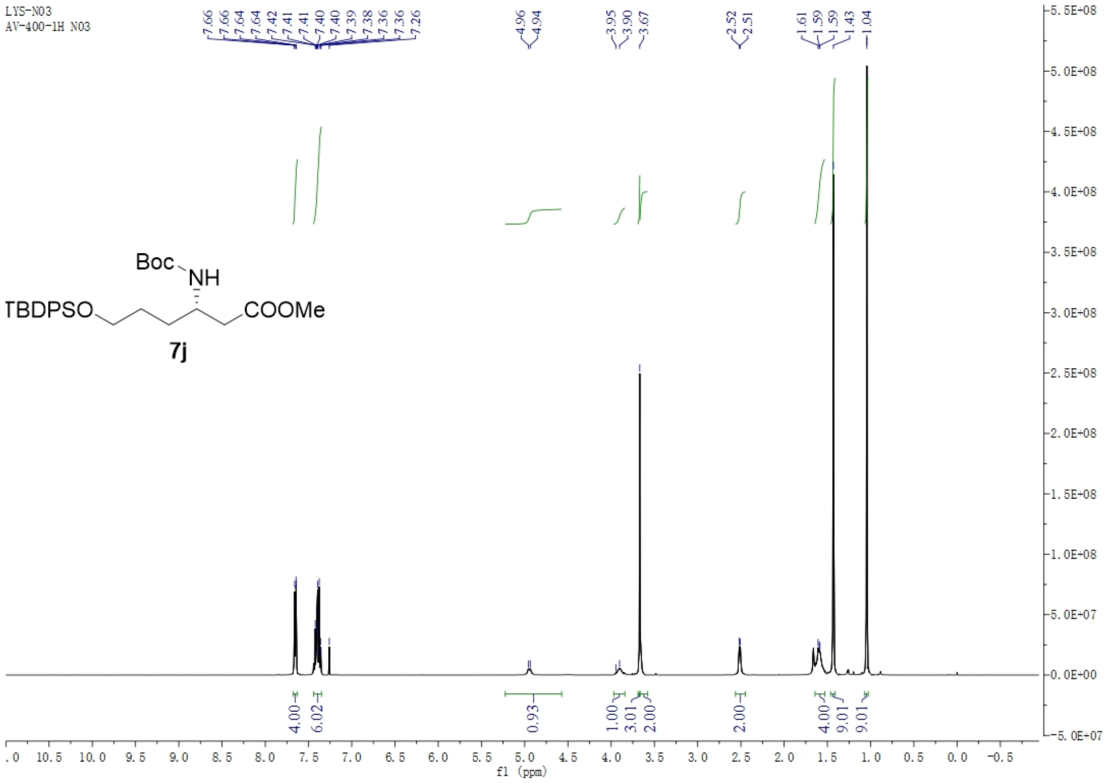

Figure S59. ^{1}H NMR of **7j** ($CDCl_3$, 600 MHz)

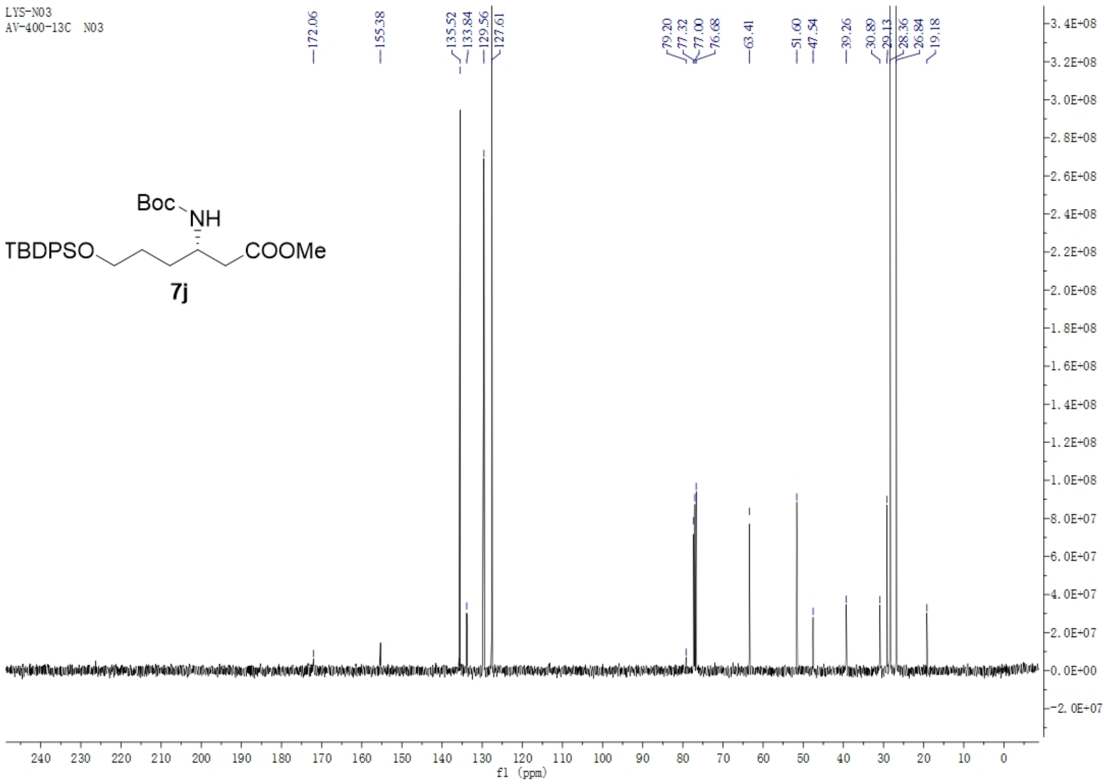

Figure S60. ^{13}C NMR of **7j** ($CDCl_3$, 150 MHz)

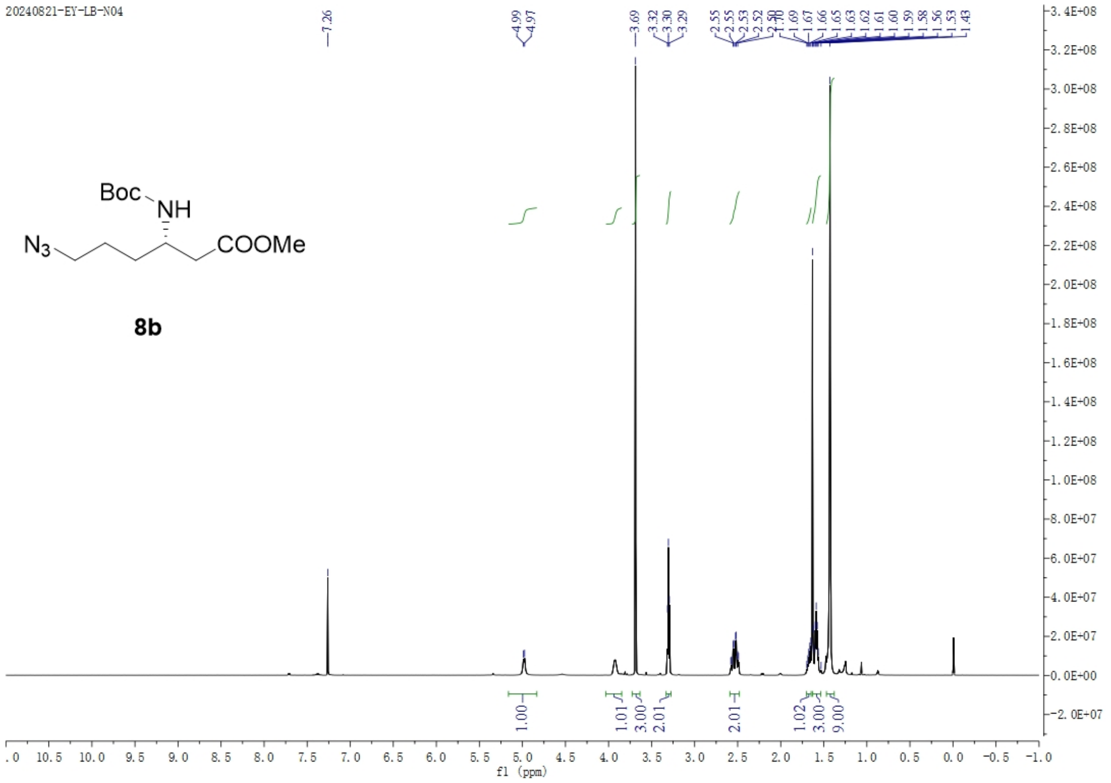

Figure S61. ^{1}H NMR of **8b** ($CDCl_3$, 600 MHz)

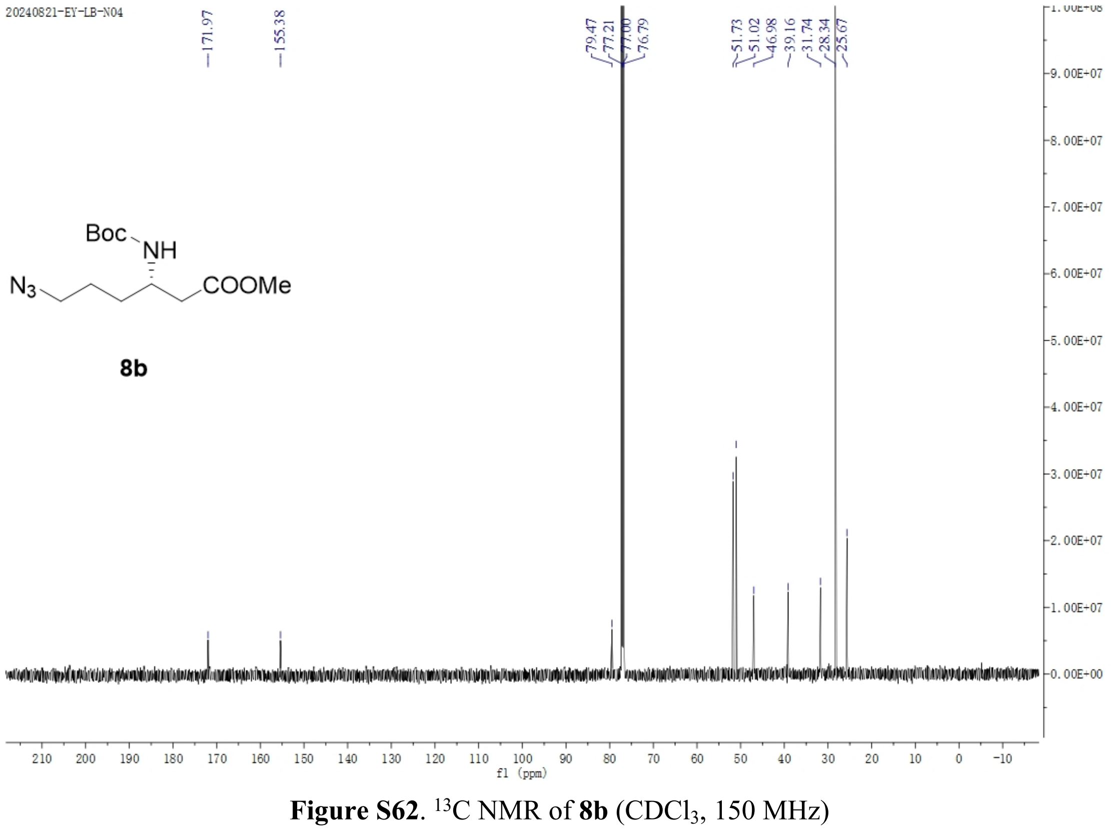

Figure S62. ^{13}C NMR of **8b** ($CDCl_3$, 150 MHz)

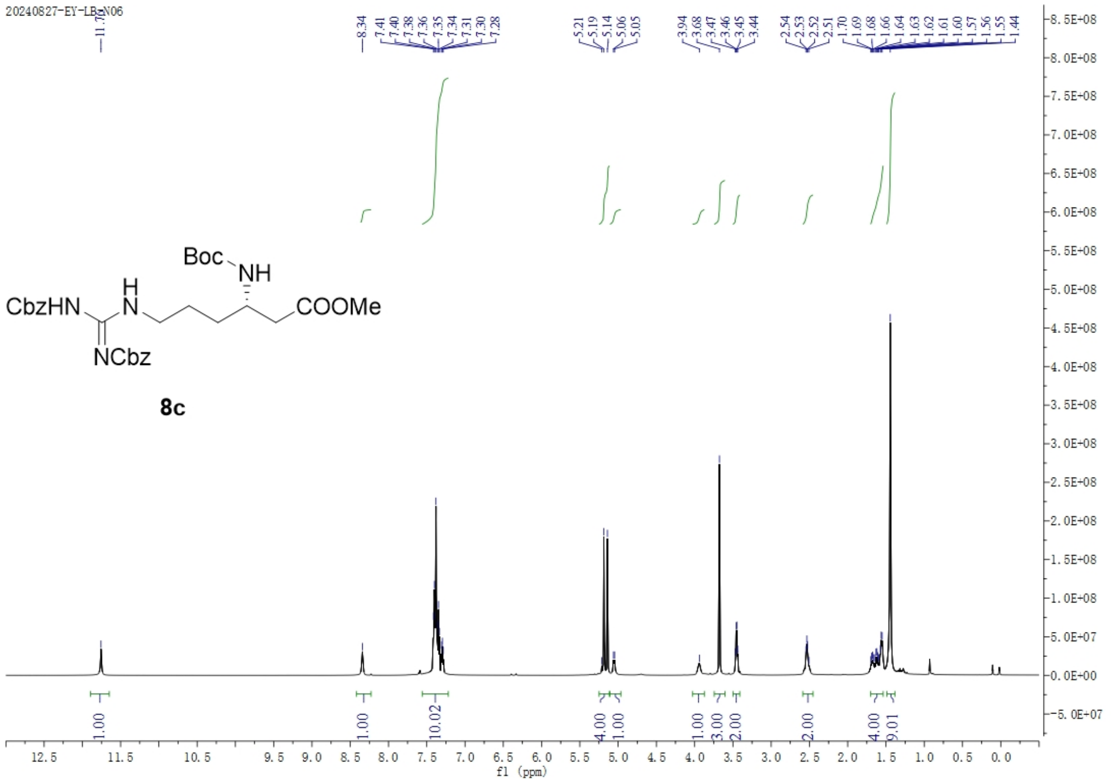

Figure S63. ^{1}H NMR of **8c** ($CDCl_3$, 600 MHz)

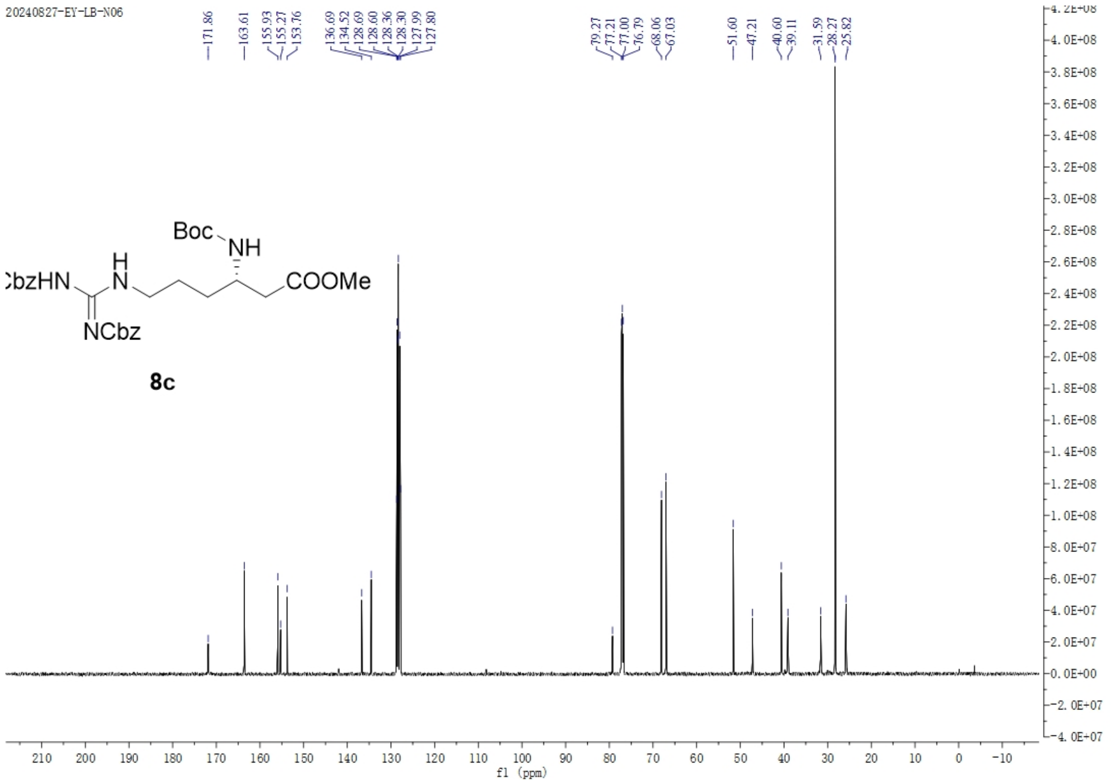

Figure S64. ^{13}C NMR of **8c** ($CDCl_3$, 150 MHz)

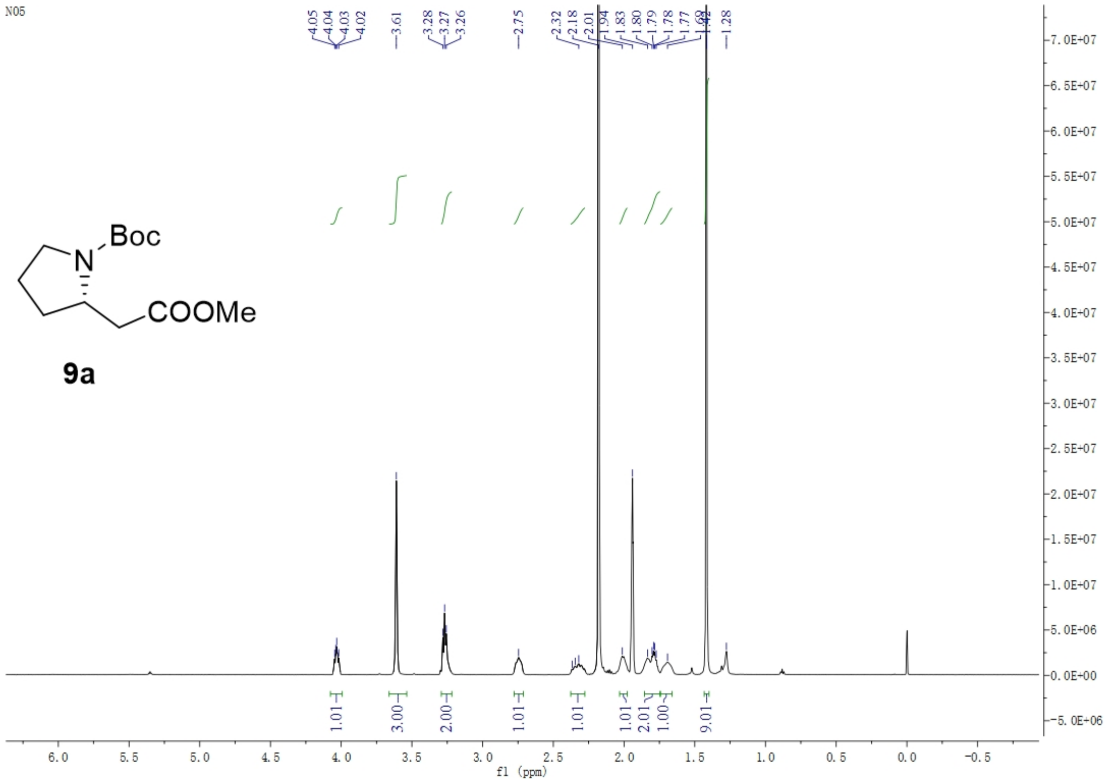

Figure S65. ^{1}H NMR of **9a** (*CD_3CN*, 600 MHz) (*exists as rotamers*)

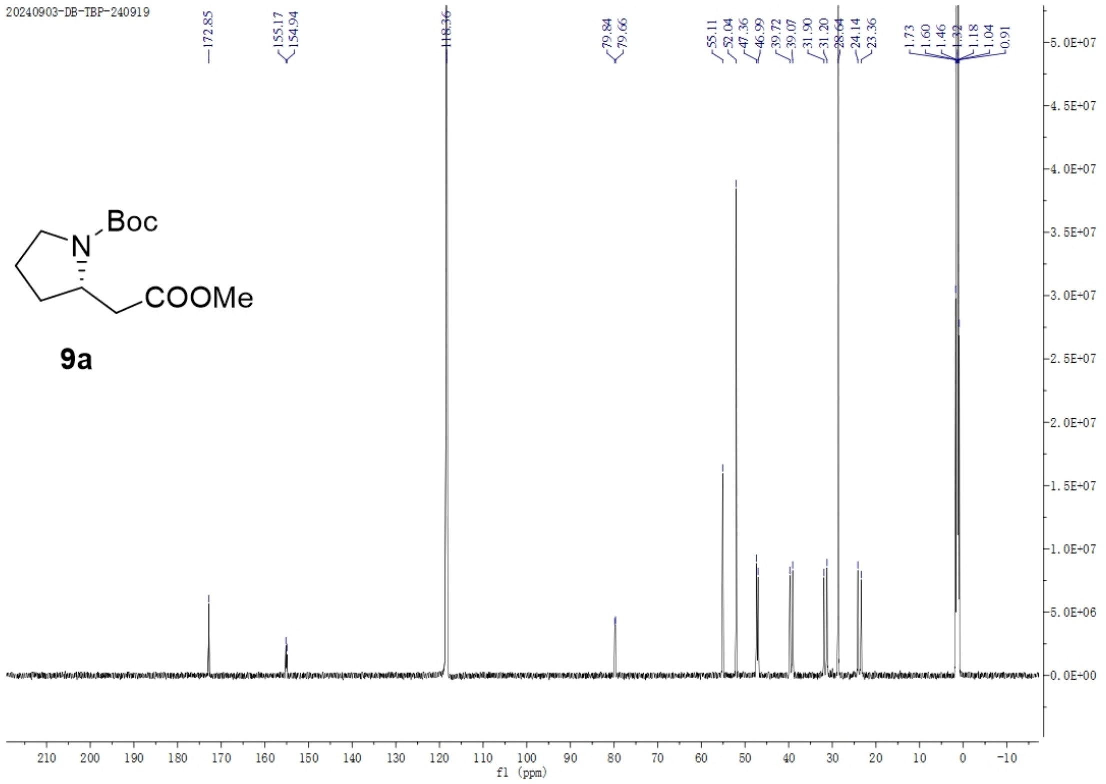

Figure S66. ^{13}C NMR of **9a** (*CD_3CN*, 150 MHz) (*exists as rotamers*)

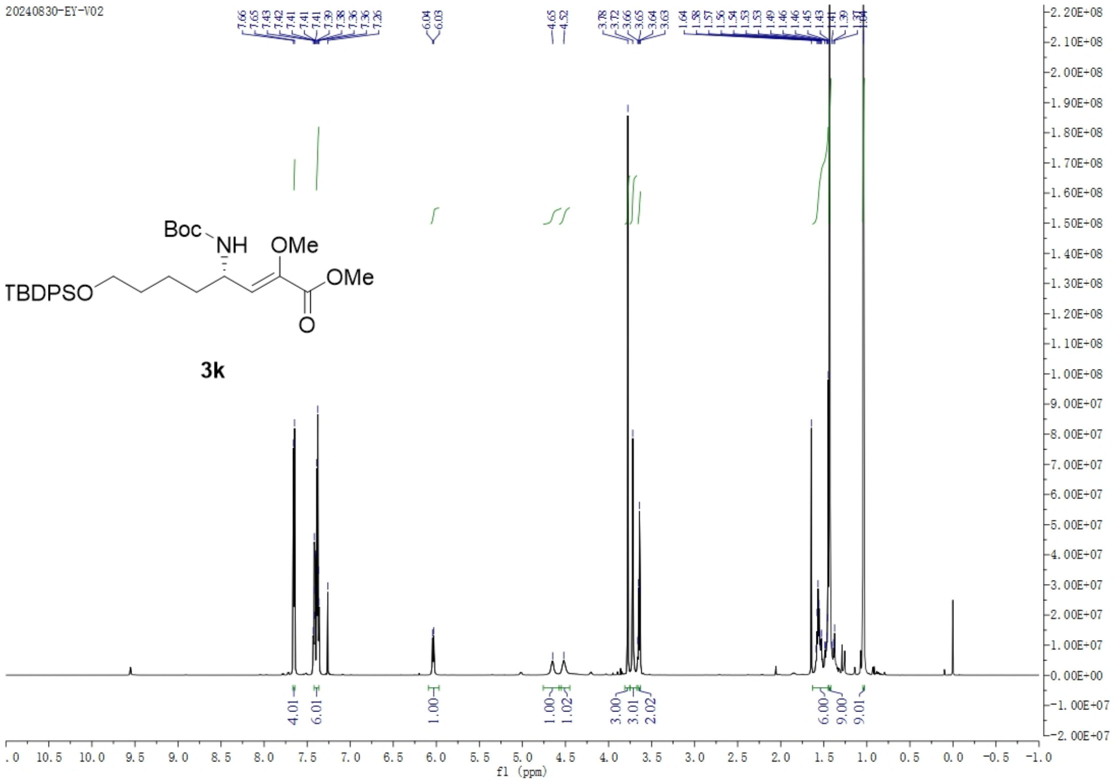

Figure S67. ^{1}H NMR of **3k** ($CDCl_3$, 600 MHz)

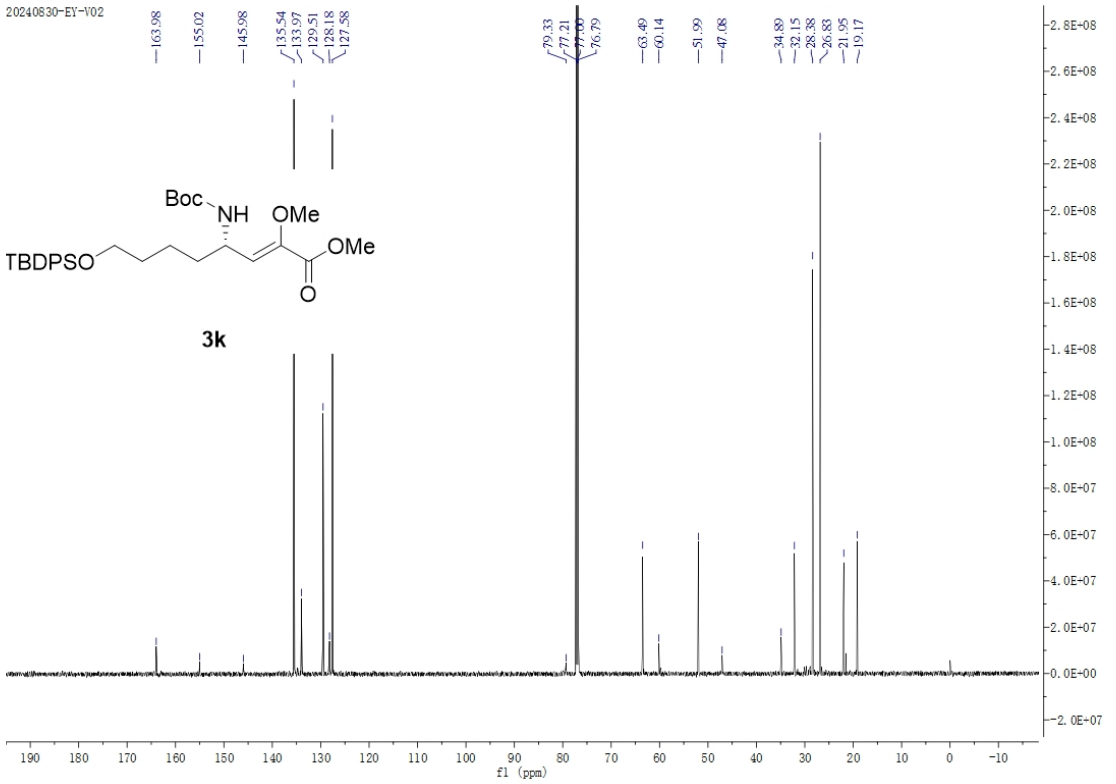

Figure S68. ^{13}C NMR of **3k** ($CDCl_3$, 150 MHz)

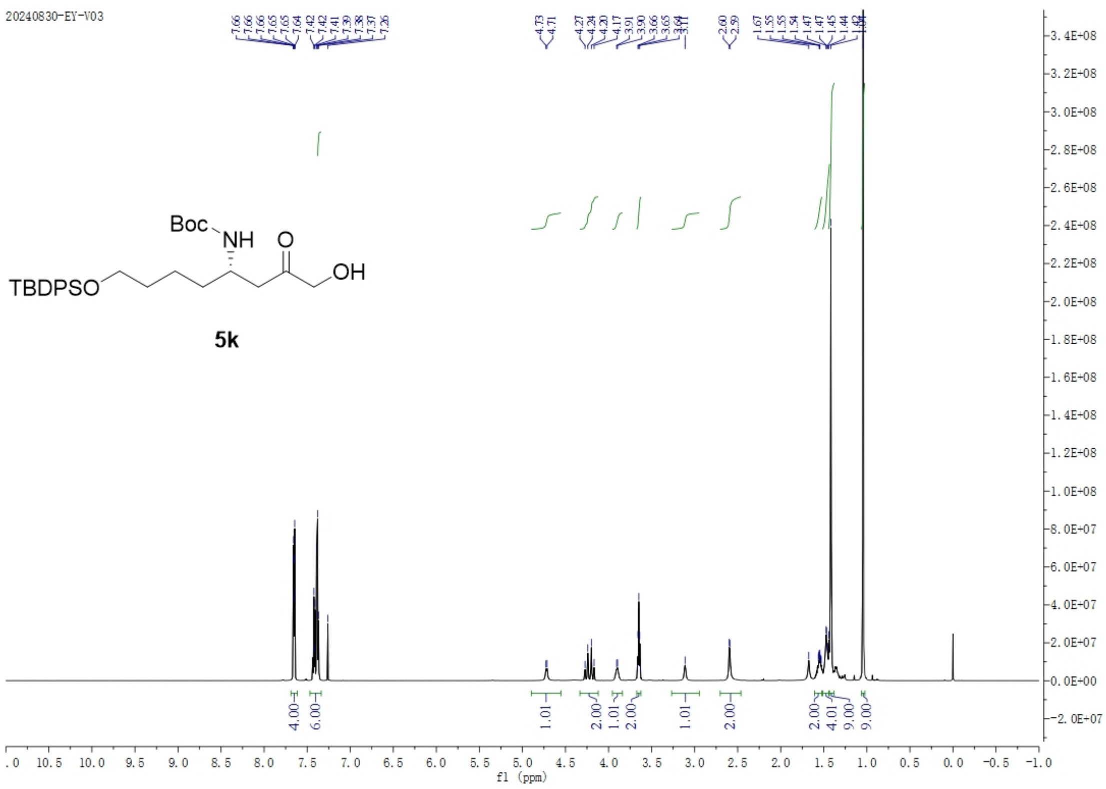

Figure S69. ^{1}H NMR of **5k** ($CDCl_3$, 600 MHz)

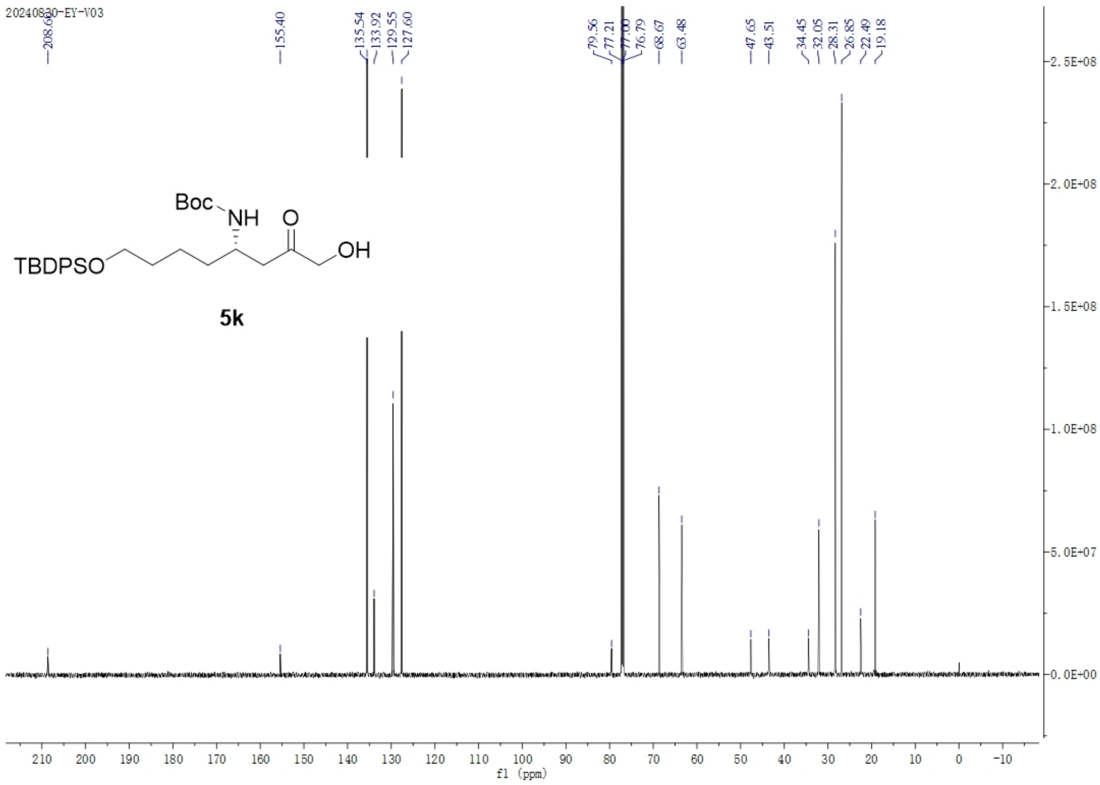

Figure S70. ^{13}C NMR of **5k** ($CDCl_3$, 150 MHz)

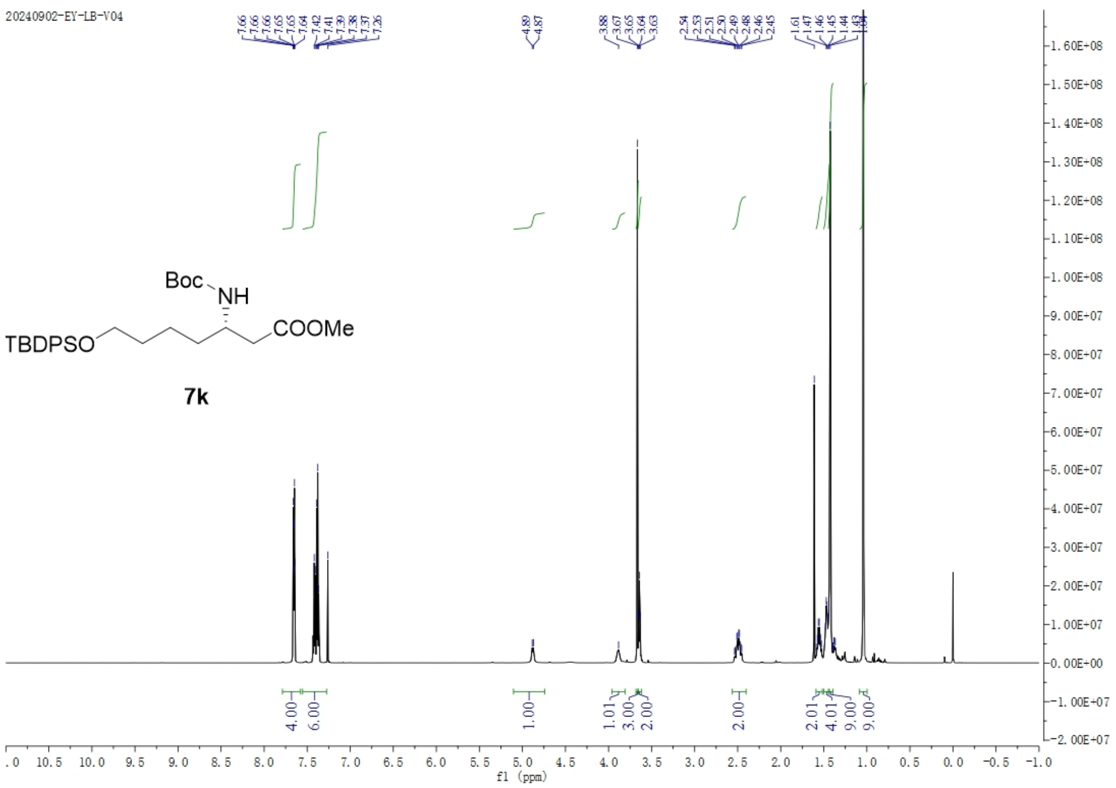

Figure S71. ^{1}H NMR of **7k** ($CDCl_3$, 600 MHz)

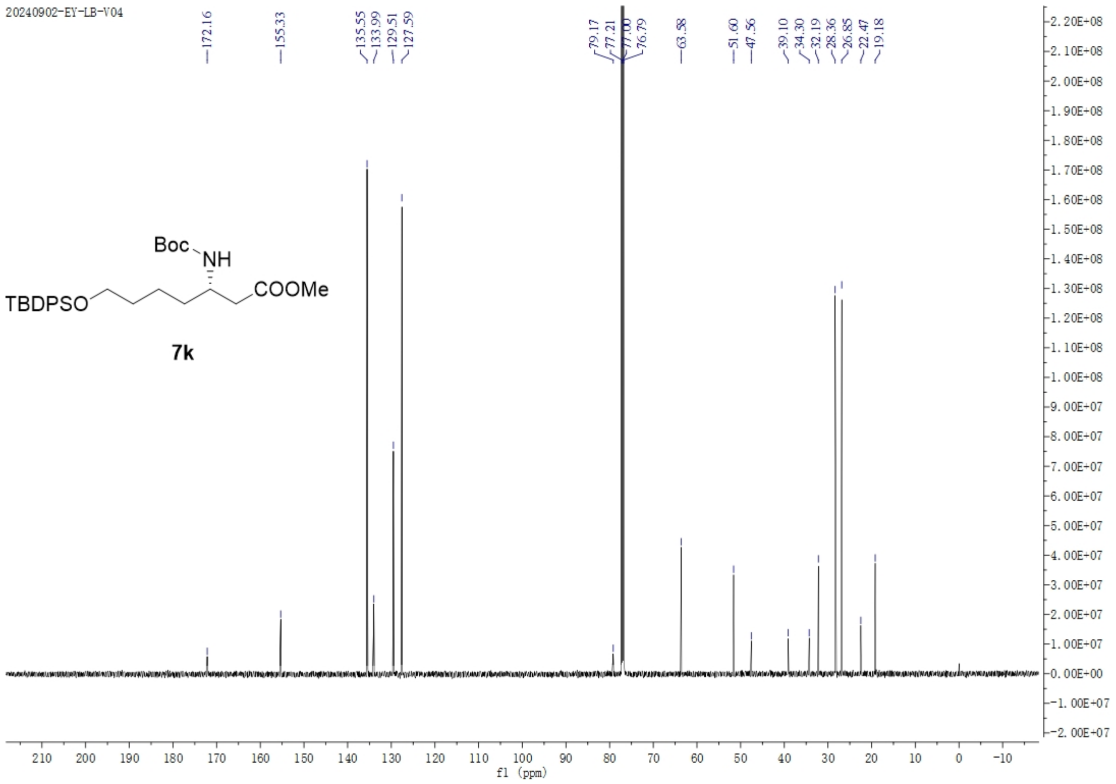

Figure S72. ^{13}C NMR of **7k** ($CDCl_3$, 150 MHz)

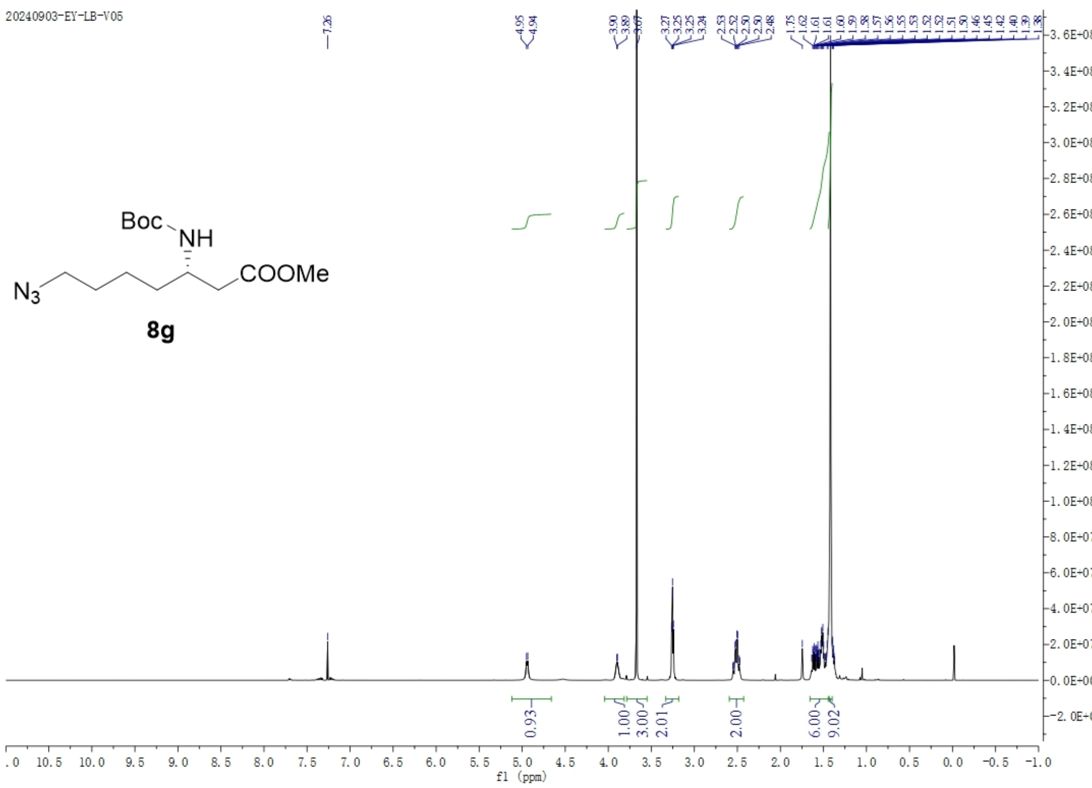

Figure S73. ^{1}H NMR of **8g** ($CDCl_3$, 600 MHz)

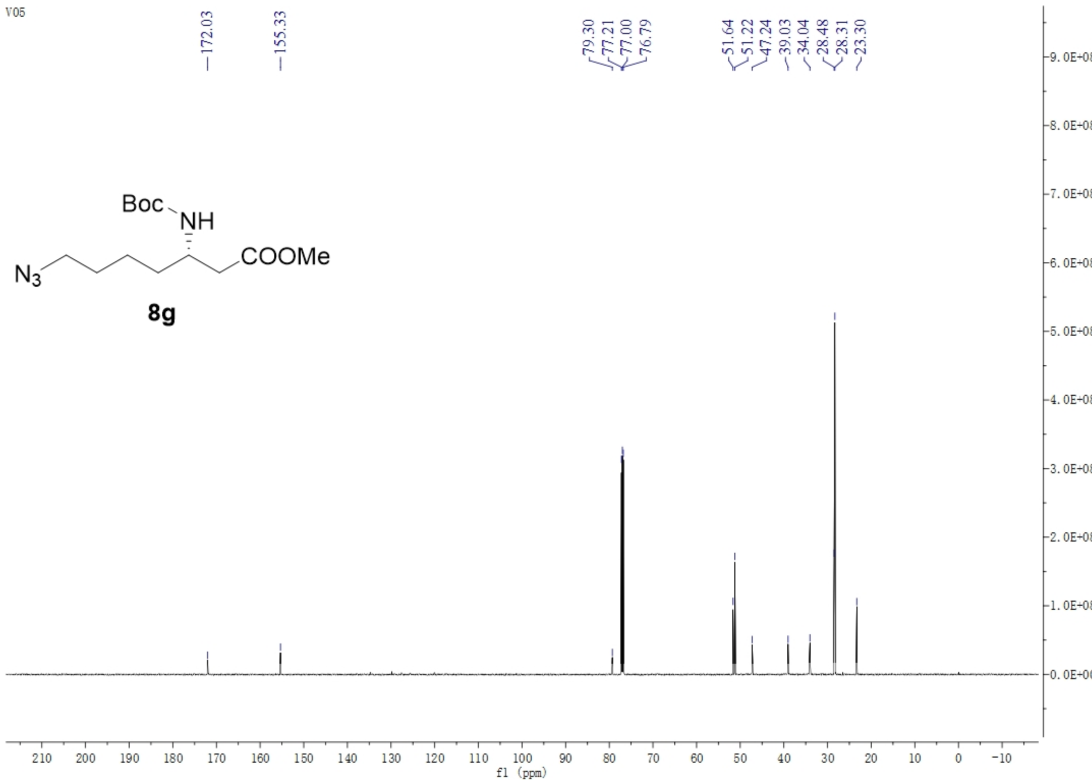

Figure S74. ^{13}C NMR of **8g** ($CDCl_3$, 150 MHz)

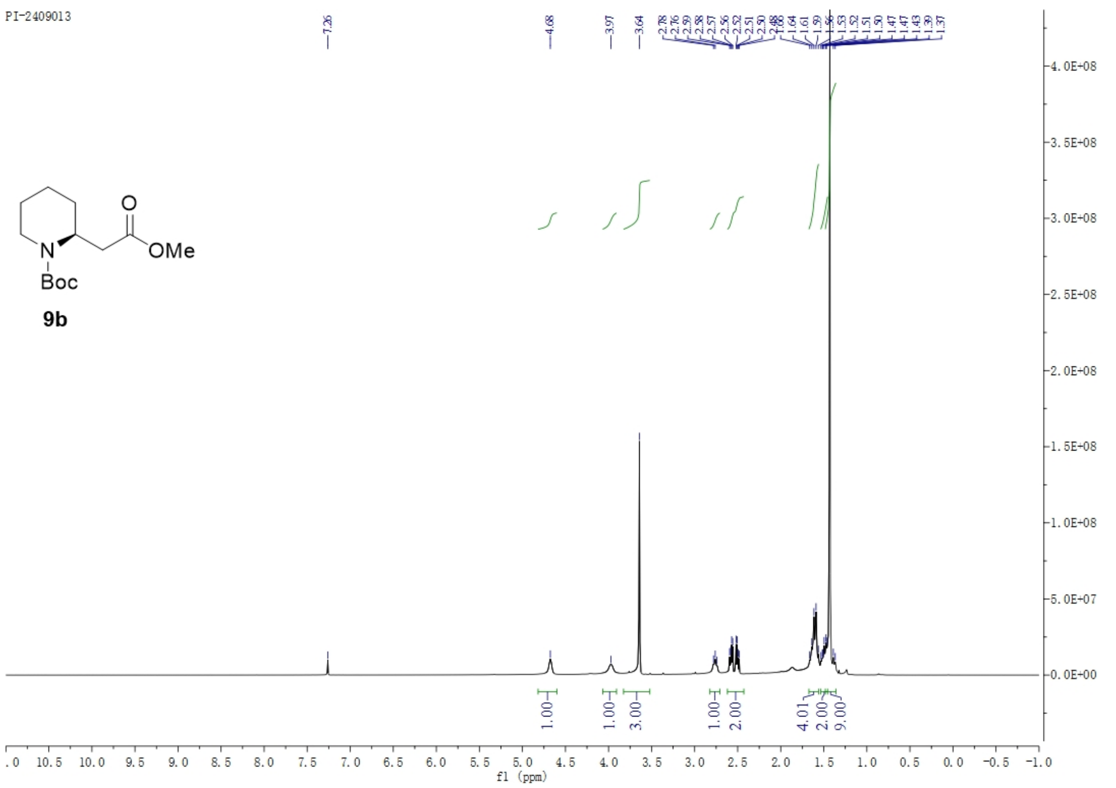

Figure S75. ^{1}H NMR of **9b** ($CDCl_3$, 600 MHz)

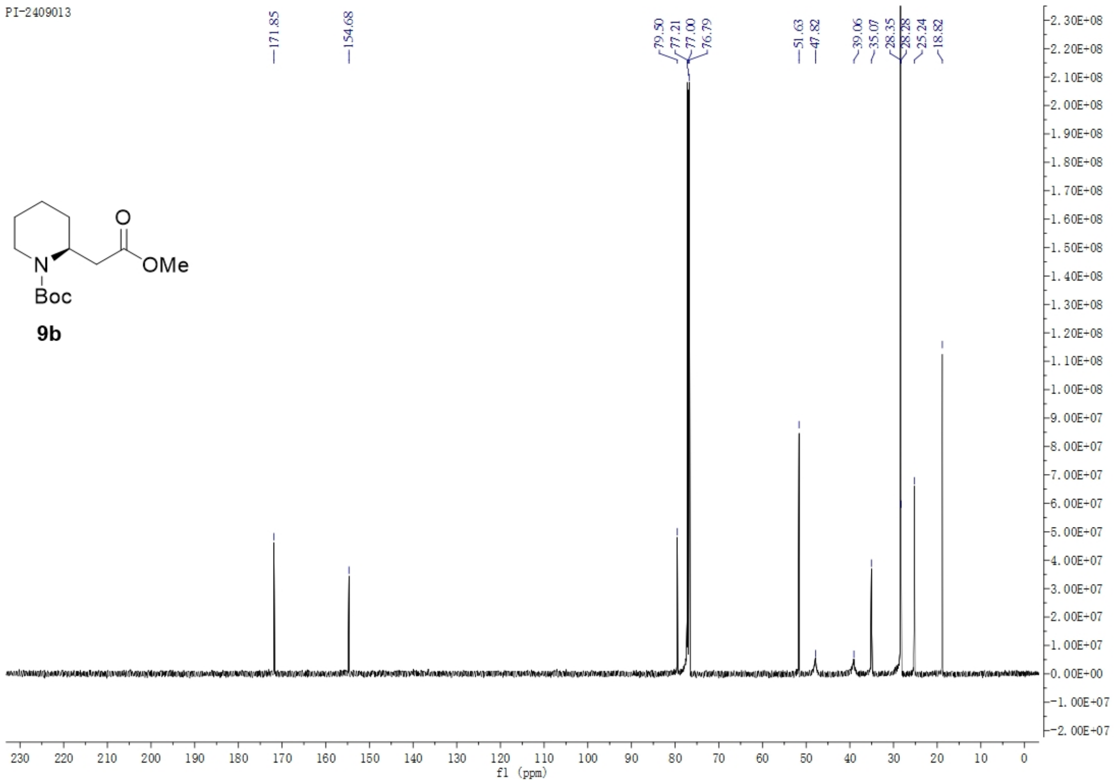

Figure S76. ^{13}C NMR of **9b** ($CDCl_3$, 150 MHz)
